# Supplementary material for: Selective hydrogenation of nitro compounds to amines by coupled redox reactions over a heterogeneous biocatalyst
Source: Nat Commun. 2024 Aug 24;15:7297. doi: 10.1038/s41467-024-51531-2 (PMC11344822; doi:10.1038/s41467-024-51531-2)
Supplement: Supplementary file 1 — Supplementary Information [file 41467_2024_51531_MOESM1_ESM.pdf]

Supplementary Information for

Selective hydrogenation of nitro compounds to amines by coupled redox reactions over a heterogeneous biocatalyst

Daria Sokolova, Tara C. Lurshay, Jack S. Rowbotham[†], Georgia Stonadge, Holly A. Reeve[‡], Sarah E. Cleary[‡]*, Tim Sudmeier*, Kylie A. Vincent*

Corresponding authors: tim.sudmeier@gmail.com; sarah@hydregenoxford.com; kylie.vincent@chem.ox.ac.uk

This file contains:

Supplementary Figures 1-78

Supplementary Tables 1-7

Supplementary Methods

Supplementary References 1-25

Table of Contents

A. Supplementary Methods **3**

I. Materials and Methods 3

II. Catalyst Preparation 5

III. Control Experiments 7

IV. Small-Scale Hydrogenation Experiments 8

V. Preparative Hydrogenation Experiments 39

VI. Preparative Gram-Scale Hydrogenation 44

VII. Exploration of the Catalyst and its Operating Conditions 46

1. Influence of the catalyst loading 47
2. Influence of MeCN as a co-solvent 48
3. Influence of pH 49
4. Influence of buffer concentration 50
5. Influence of substrate concentration 51

VIII. Catalyst Recycling 52

IX. Electrochemical Procedures 53

1. Voltammetry of Hyd-1 and nitrobenzene 54
2. Enzyme film voltammograms 56
3. Determination of substrate reduction onset potentials 58

X. Extension of Small-Scale Hydrogenation to Aliphatic Substrate 87

B. Supplementary References **92**

A. Supplementary Methods

I. Materials and Methods

Buffer salts and HPLC grade solvents were purchased from SIGMA-ALDRICH. Deuterated solvents were purchased from THERMO SCIENTIFIC ($CDCl_3$, 99.8% D; MeOH-d_4, ≥99.8% D), SIGMA-ALDRICH (D_2O, 99.9% D), and VWR CHEMICALS (DMSO-d_6, 99.8% D).
Nitrobenzene (99%) was purchased from ALFA AESAR; *N*-phenylhydroxylamine (≥95%), nitrosobenzene (≥97%), 4-nitrotoluene (99%), 4-nitrophenol (≥99%), 1,2-dinitrobenzene (97%), 1,3-dinitrobenzene (97%), 1,4-dinitrobenzene (98%), 1-fluoro-4-nitrobenzene (99%), 1-chloro-4-nitrobenzene (99%), 1-nitronaphtalene (99%), benzyl viologen dichloride (97%), *N,N*-diethylethylenediamine (≥99%), triethylamine (≥99%), 3-chloroperbenzoic acid (≤77%), 1-nitrohexane (98%), hexylamine (99%), and 4-nitrobenzoyl chloride (98%) were purchased from SIGMA-ALDRICH; 2-nitrotoluene (99%), 3-nitrotoluene (95%), 2-nitrophenol (95%), 3-nitrophenol (99%), 1-bromo-2-nitrobenzene (98%), 1-bromo-3-nitrobenzene (99%), 1-bromo-4-nitrobenzene (95%), 1-iodo-4-nitrobenzene (95%), 4-nitrobenzyl alcohol (99%), 4-nitrobenzaldehyde (98%), 4-nitrobenzoic acid (98%), ethyl 4-nitrobenzoate (95%), 4'-nitroacetophenone (95%), 4-nitrobenzotrifluoride (98%), 1-*tert*-butyl-2-nitrobenzene (95%), 1-*tert*-butyl-nitrobenzene (95%), 4-nitrothiophenol (90%), 4-nitrobenzonitrile (97%), 1-ethynyl-4-nitrobenzene (95%), 5-nitrosalicylic acid (95%) were purchased from FLUOROCHEM; 4-nitrostyrene (98%) was purchased from THERMO SCIENTIFIC. Product standards 4-aminostyrene (96%) and 4-aminothiophenol (97%) were purchased from FLUOROCHEM. Carbon black (carbon, C) BLACK PEARLS® 2000 was purchased from CABOT.

All the chemicals and solvents were used as received without prior purification, except for 4-aminothiophenol, which was purified by column chromatography (silica gel, hexane/EtOAc = 5:1, R_f = 0.31).

All aqueous solutions were prepared with deoxygenated MilliQ water (Millipore, 18 MΩcm).

^{1}H-NMR spectra were recorded at 400 MHz on a BRUKER AVANCE III HD NANOBAY NMR spectrometer equipped with a 9.4T magnet. Chemical shifts of ^{1}H-NMR spectra (measured at 298 K) are given in ppm by using residual solvent signals as references ($CDCl_3$: 7.26 ppm; D_2O: 4.79 ppm; DMSO-d_6: 2.50 ppm; methanol-d_4: 4.78 ppm and 3.31 ppm). Coupling constants (J) are reported in Hertz (Hz). Standard abbreviations indicating multiplicity were used as follows: s (singlet), brs (broad singlet), d (doublet), dd (doublet of doublets), t (triplet), q (quadruplet), m (multiplet). ^{1}H-NMR signals for samples in 10% D_2O in sodium phosphate buffer (PB, concentration and pH are specified in each case) were measured with the water suppression method. NMR-based yields reported by analysis of ^{1}H-NMR signals measured in 10% D_2O in PB with the quantitative water suppression method in the presence of internal standard (4-nitrophenol).

Analytical thin-layer chromatography (TLC) was performed on MERCK silica gel 60 F254 aluminum plates, which were analysed by fluorescence detection with UV-light (λ = 254 nm, [UV]) and after exposure to standard staining reagents and subsequent heat treatment. The following staining solution was used: potassium permanganate [$KMnO_4$] (1.5 g potassium permanganate, 10 g potassium carbonate, sodium hydroxide (10% aqueous solution) in 200 mL water). Purification by column chromatography was performed using Geduran® Si 60 (40-63 µm) purchased from SIGMA-ALDRICH.

The Hydrogenase-1 (Hyd-1) genes were produced according to the reported protocol and purified using a Ni-affinity column.[1] The yield of Hyd-1 was ~0.5 mg of protein per liter of culture. The activity of the overexpressed Hyd-1 was verified using a spectrophotometric assay measuring the hydrogenase-mediated reduction of benzyl viologen dichloride in an H_2-saturated solution. The specific activity of (32 ± 1) nmol·min^{-1}·mg^{-1} was measured on three independent hydrogenase-mediated benzyl viologen dichloride reduction assays. Another batch of Hyd-1 (1.9 mg of protein per liter of culture, similar activity to the previous batch) was prepared according to the same procedure to run a large-scale experiment.

The Hydrogenase-2 (Hyd-2) enzyme was produced according to the reported protocol and purified using a Ni-affinity column.[2] The yield of Hyd-2 was ~0.2 mg of protein per liter of culture. The activity of the overexpressed Hyd-2 was verified using a spectrophotometric assay measuring the hydrogenase-mediated reduction of benzyl viologen dichloride in an H_2-saturated solution. The specific activity of (4.18 ± 0.2) µmol·min^{-1}·mg^{-1} was measured with three independent hydrogenase-mediated benzyl viologen dichloride reduction assays.

II. Catalyst Preparation

Catalyst preparation was carried out in a glove box (GLOVE BOX TECHNOLOGY LTD.) under a protective N_2 atmosphere (O_2 < 3 ppm). A 20 mg/mL carbon black suspension in PB (50 mM, pH 6.0 unless stated otherwise) was sonicated for 1 hour. For the preparation of the catalyst for one 2 mL scale reaction with 10 mM concentration of substrate, 52.8 µL of this suspension was transferred to an Eppendorf tube, 15.4 µL of Hyd-1 solution (1.71 mg/mL) was added (C:Hyd-1 = 40:1 mass ratio), the mixture was gently mixed and left in the fridge (4 °C) for 1 hour. After that, the suspension of the catalyst was centrifuged (3 min, 14100 × g), the supernatant was removed by pipetting, and the catalyst was resuspended in 100 µL of PB (50 mM, pH 6.0 unless stated otherwise). Resuspension-centrifugation-pipetting steps were repeated 3 times, and then the catalyst was resuspended in 100 µL of PB and then directly used for the reaction or frozen in liquid N_2 and stored at -80 °C.

<u>*Note:*</u> after numerous preliminary screenings the optimal ratio of carbon to Hyd-1 (C:Hyd-1) to reach full conversion of various substrates to corresponding amines was found to be **40:1 mass ratio** with corresponding catalyst loading of **1.32 mg of Hyd-1 per 1 mmol of substrate**. A further variation of C:Hyd-1 ratio in reactions with nitrobenzene (**1**) as the model substrate confirmed the determined ratio of 40:1 as the optimal one to reach full conversion of nitrobenzene (**1**) to aniline (**1a**) as other tested ratios of C:Hyd-1 gave mixtures of **1a** and *N*-phenylhydroxylamine (**1b**) (Supplementary Table 1, Supplementary Fig. 1).

Supplementary Fig. 2 illustrates reference ^{1}H-NMR spectra of all possible major components of the reaction mixture, including **1** as a starting material, nitrosobenzene (**1c**) and **1b** as possible intermediates, and final product **1a**. Careful analysis of the reaction mixtures at different time points suggests that **1c** is not being formed during hydrogenation of **1** using Hyd-1/C catalyst, and reaction proceeds *via* rapid formation of **1b** followed by its reduction to **1a** (Fig. 4B of the Manuscript). According to the ^{1}H-NMR spectra there are no other intermediates and side products formed.

Supplementary Table 1. Results on variation of C:Hyd-1 ratio for the catalyst preparation for hydrogenation of **1**. The optimal catalyst with C:Hyd-1 = 40:1 mass ratio is highlighted in blue. Conversions and ratios of products were determined by integration of ^{1}H-NMR spectra. All reactions were run for 24 hours at 10 mM concentration of **1** in PB (50 mM, pH 6.0) using 1.32 mg of Hyd-1 immobilised on carbon per 1 mmol of substrate (see Section IV).

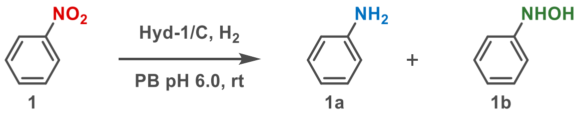

Entry	C:Hyd-1 ratio	Conversion of 1 (%)	1b:1a
1	5:1	18	100:0
2	10:1	48	100:0
3	20:1	65	100:0
4	30:1	93	70:30
5	40:1	100	0:100
6	50:1	100	11:89

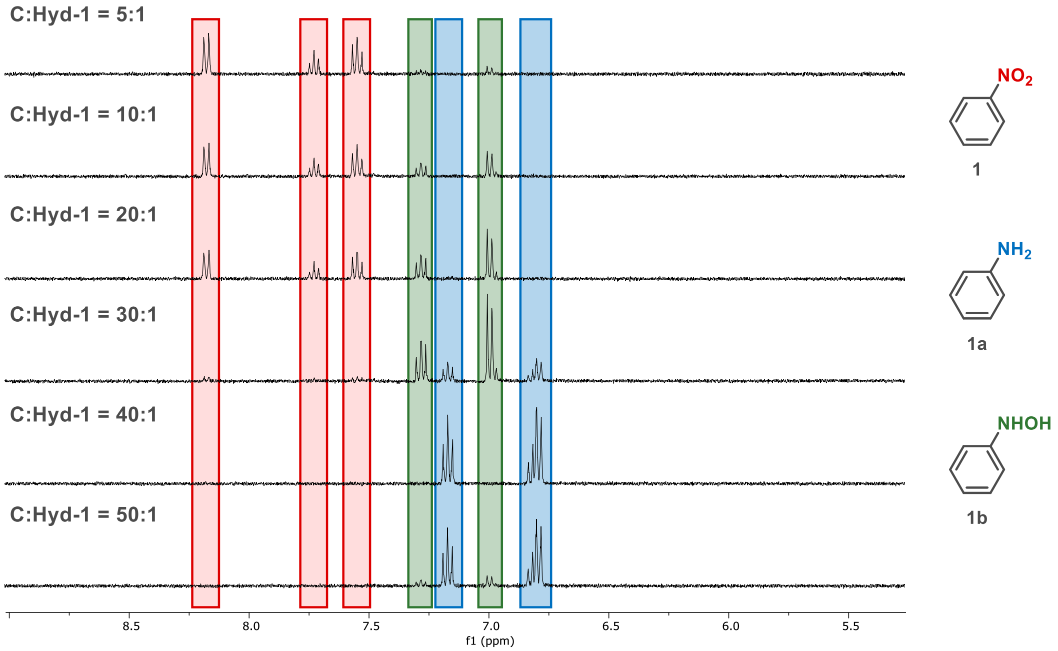

Supplementary Figure 1. ^{1}H-NMR spectra (400 MHz, 298 K, 10% D_2O in PB, 50 mM, pH 6.0) of nitrobenzene (**1**, red markers) hydrogenation reaction mixtures after 24 hours at 10 mM concentration of **1** in PB (50 mM, pH 6.0) using 1.32 mg of Hyd-1 immobilised on carbon per 1 mmol of substrate with variation of C:Hyd-1 ratio for the catalyst preparation (indicated on the left side of each spectrum). *N*-Phenylhydroxylamine (**1b**) is labelled with green markers, aniline (**1a**) is labelled with blue markers.

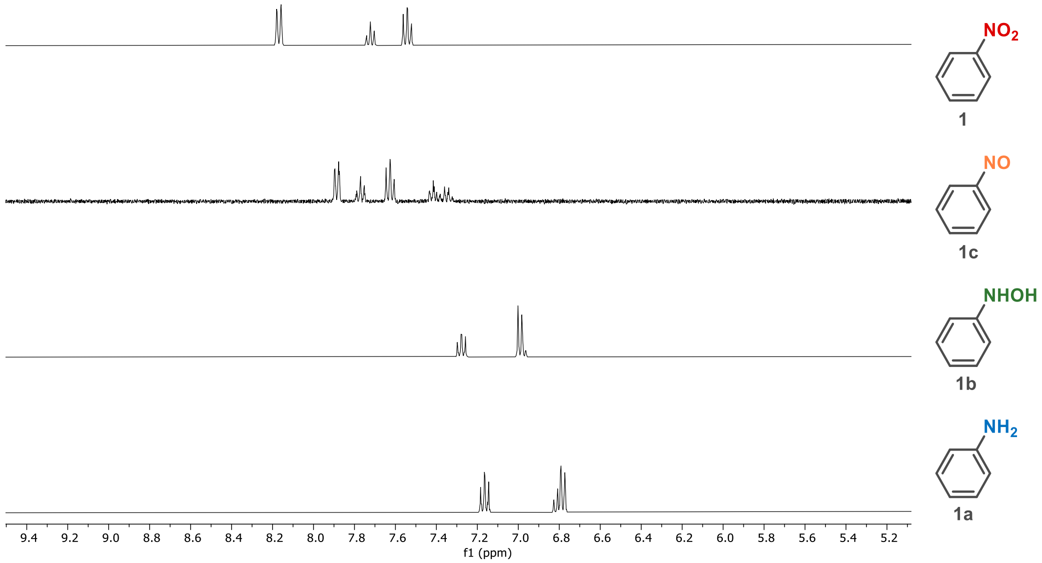

Supplementary Figure 2. ^{1}H-NMR spectra (400 MHz, 298 K, 10% D_2O in PB, 50mM, pH 6.0) of standards of nitrobenzene (**1**), nitrosobenzene (**1c**), *N*-phenylhydroxylamine (**1b**), and aniline (**1a**).

III. Control Experiments

Control experiments in presence of Hyd-1/C catalyst, Hyd-1 alone, and carbon black alone were carried out according to Section IV of the SI at 10 mM nitrobenzene concentration in PB (50 mM, pH 6.0) and standard catalyst loading (1.32 mg of Hyd-1 per 1 mmol of substrate, C:Hyd-1 = 40:1 mass ratio), using nitrobenzene (**1**) as a model substrate. The same amounts of Hyd-1 and carbon black alone were used as for the preparation of the catalyst. Results of this screening show no evidence of hydrogenation of **1** to aniline (**1a**) in presence of Hyd-1 or carbon black alone as conversion of starting material was observed in any of these cases. Full conversion to **1a** was only observed in presence of Hyd-1/C catalyst (Supplementary Fig. 3).

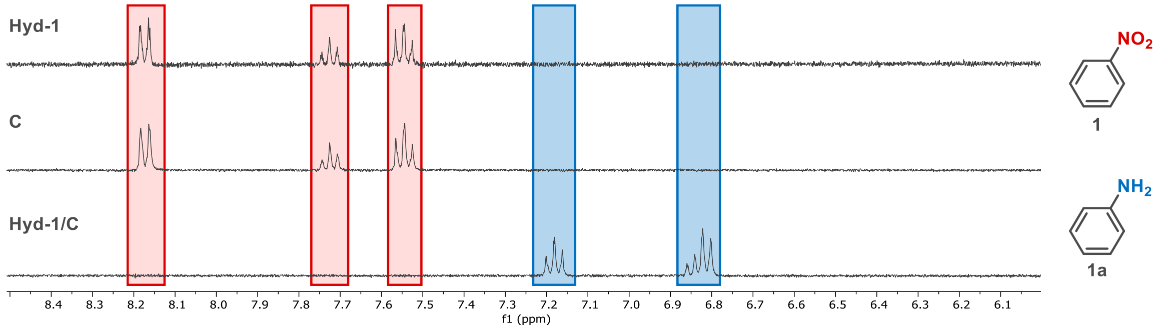

Supplementary Figure 3. ^{1}H-NMR spectra (400 MHz, 298 K, 10% D_2O in PB, 50 mM, pH 6.0) of **1** (red markers) hydrogenation reaction mixtures after 24 hours with either Hyd-1 enzyme, carbon black (C), or Hyd-1/C catalyst. Full conversion to aniline (**1a**, blue markers) was only observed in presence of Hyd-1/C catalyst.

To confirm that theoretically possible intermediates, nitrosobenzene (**1c**) and *N*-phenylhydroxylamine (**1b**), can be reduced under the reaction conditions, control experiments were carried out according to as described in Section IV at 10 mM substrate concentration in PB (50 mM, pH 6.0) and standard catalyst loading (1.32 mg of Hyd-1 per 1 mmol of substrate, C:Hyd-1 = 40:1 mass ratio), using **1b** or **1c** as substrates (Supplementary Fig. 4). After 24 hours of reaction time **1c** was fully converted to 30:70 mixture of **1b** and aniline (**1a**). At the same time, **1b** was fully converted to **1a**, confirming that **1b** formed during hydrogenation of nitrobenzene (**1**) can be further reduced to **1a**.

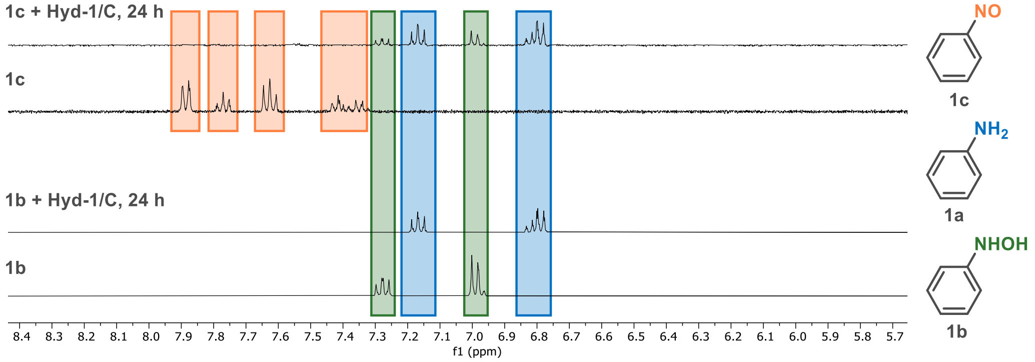

Supplementary Figure 4. ^{1}H-NMR spectra (400 MHz, 298 K, 10% D_2O in PB, 50 mM, pH 6.0) of nitrosobenzene **1c** (orange markers) and *N*-phenylhydroxylamine **1b** (green markers) hydrogenation reaction mixtures after 24 hours with Hyd-1/C catalyst. Conversion to aniline (**1a**, blue markers) was observed in both cases.

IV. Small-Scale Hydrogenation Experiments

General Procedure: Reaction set-up was carried out in a glove box (Glove Box Technology Ltd.) under a protective N_2 atmosphere (O_2 < 3 ppm). Reactions were run on a 2 mL scale with a 10 mM concentration of substrate in PB (50 mM, pH 6.0 unless stated otherwise) or with 10% v/v of MeCN at room temperature under a gentle H_2 flow in an ASYNT OCTO MINI REACTOR, which allows running eight reactions in parallel (Supplementary Fig. 5). A stock solution of substrate in buffer or MeCN was transferred to a reaction vessel, 100 µL of catalyst was added, and the volume was adjusted with the corresponding buffer to a total volume of 2 mL. The reactor was closed and removed from the glove box. The hydrogen line was connected, and reactions were run at a 30-40 mL/min flow of H_2. Time points were taken at 24, 48, and 72 hours and analysed by ^{1}H-NMR spectroscopy. To prepare a sample for the ^{1}H-NMR analysis, an aliquot of 480 µL of the reaction mixture was centrifuged (3 min, 14100 × g), 450 µL of supernatant was placed in the NMR tube, and 50 µL of D_2O was added. ^{1}H-NMR spectra (Supplementary Fig. 6-35) were measured with water signal suppression and represent comparison of signals of each starting material with the corresponding amine product after completion of each reaction. The signal observed around 2 ppm for some of the ^{1}H-NMR spectra corresponds to MeCN.

Note: substrates **8**, **12**, **14**, **17**–**20**, **23**, **24**, **26**, and **30** required double catalyst loading; for substrate **27** quadruple catalyst loading was used; substrate **28** required double catalyst loading and pH 8.0 (PB, 50 mM). Stock solutions of substrates **4**, **8**–**13**, **15**–**18**, **20**–**24**, **26**–**30** were prepared in MeCN so that the total amount of MeCN in the reaction mixture was 10% v/v.

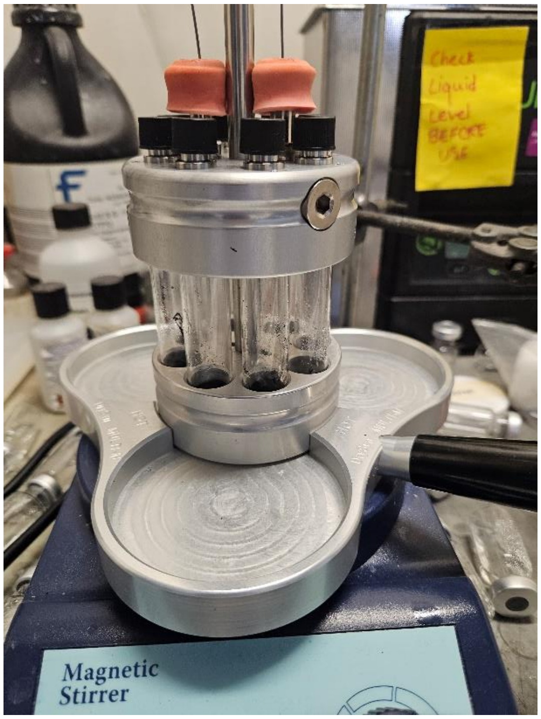

Supplementary Figure 5. Reaction set-up with ASYNT OCTO MINI REACTOR for running small-scale hydrogenations reactions.

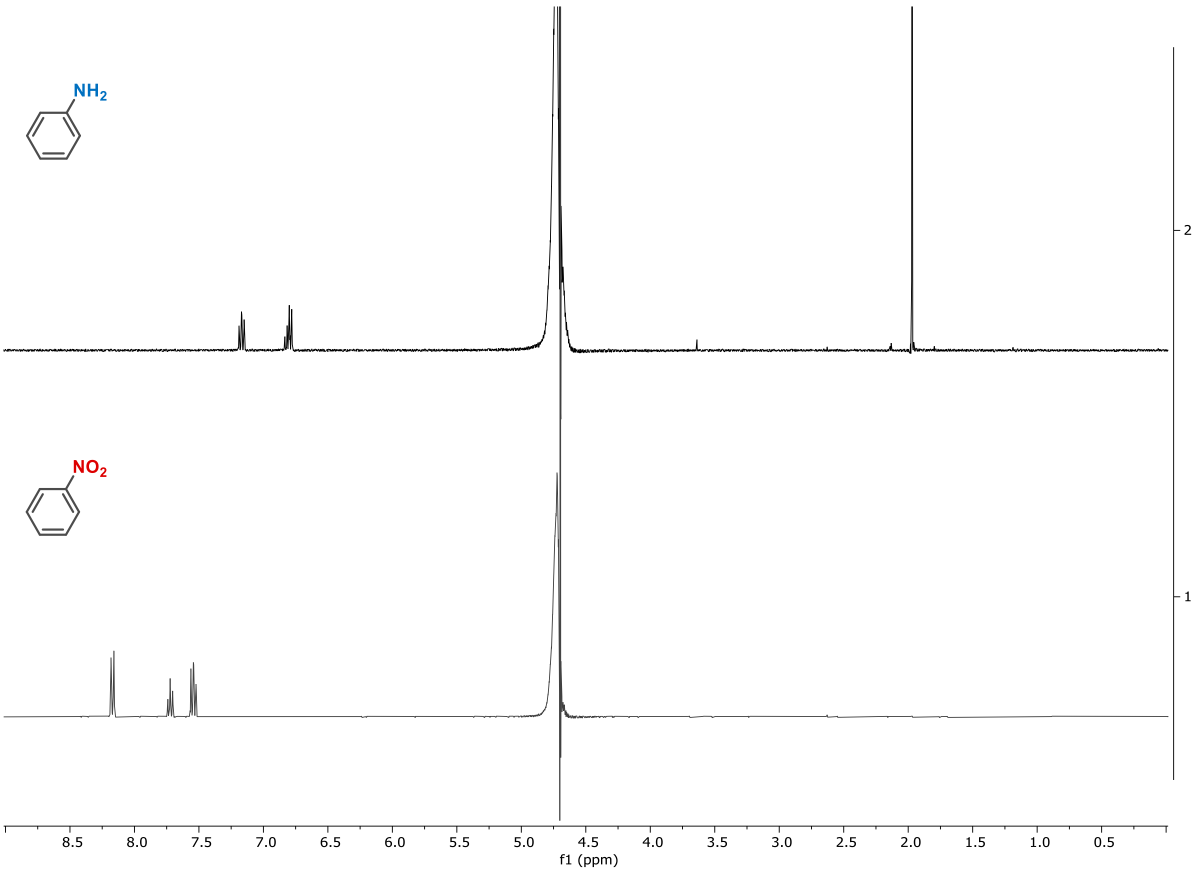

Supplementary Figure 6. ^{1}H-NMR spectra (400 MHz, 298 K, 10% D_2O in PB, 50 mM, pH 6.0) of substrate **1** and reaction mixture after 12 hours of reaction time.

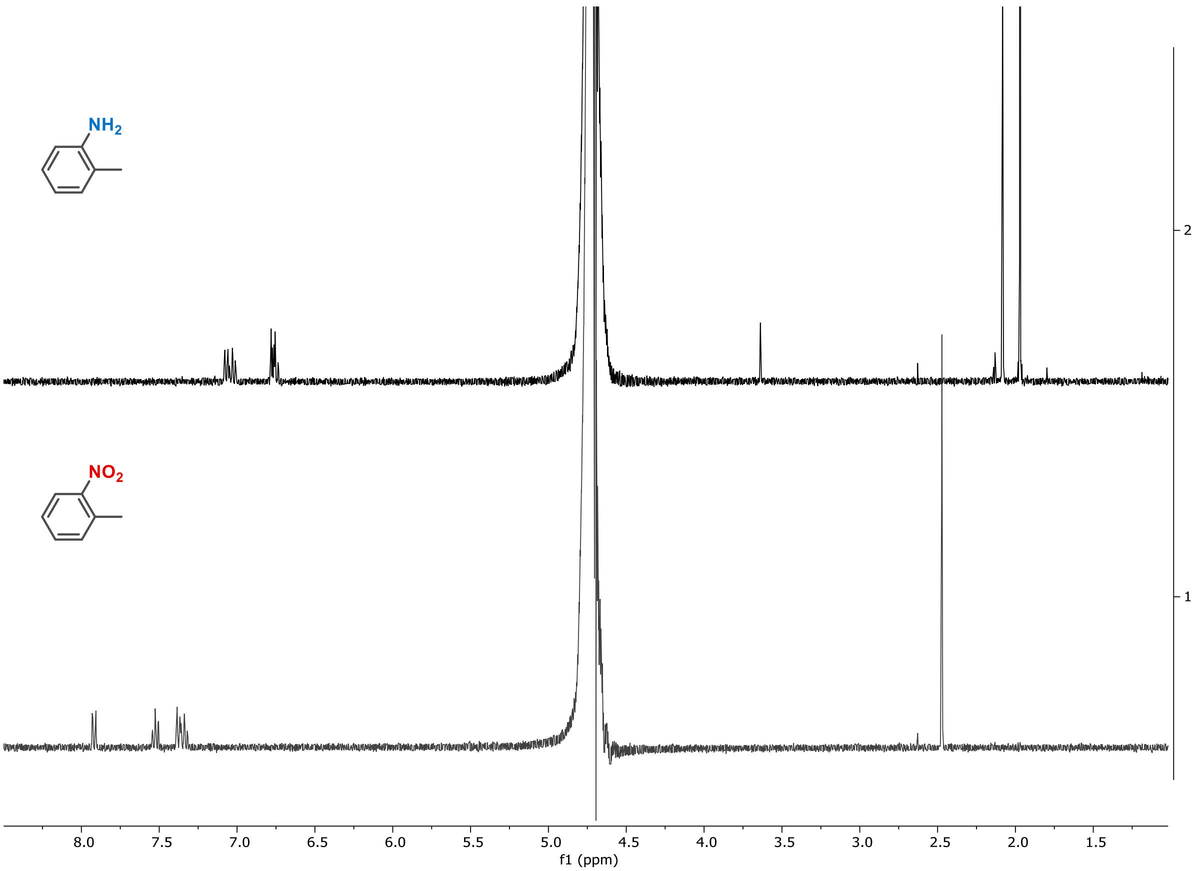

Supplementary Figure 7. ^{1}H-NMR spectra (400 MHz, 298 K, 10% D_2O in PB, 50 mM, pH 6.0) of substrate **2** and reaction mixture after 24 hours of reaction time.

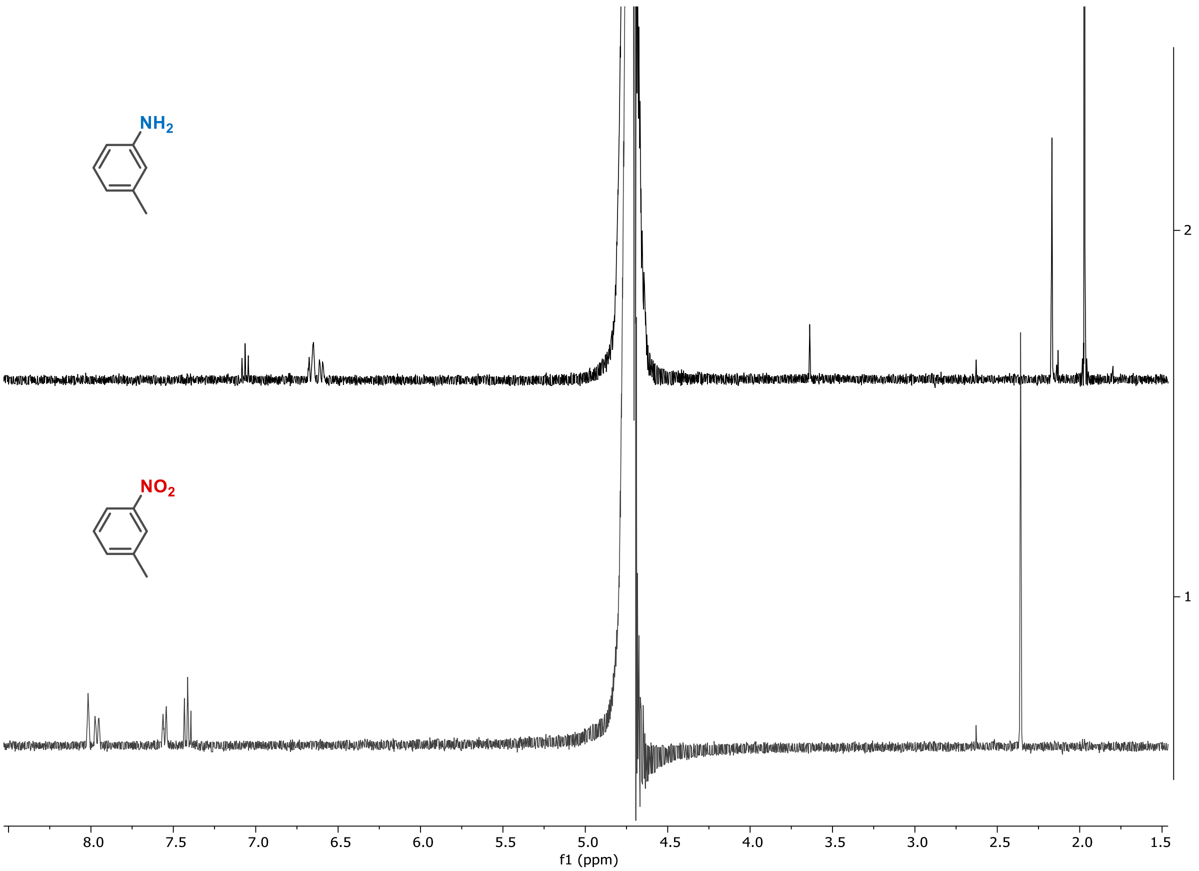

Supplementary Figure 8. ^{1}H-NMR spectra (400 MHz, 298 K, 10% D_2O in PB, 50 mM, pH 6.0) of substrate **3** and reaction mixture after 24 hours of reaction time.

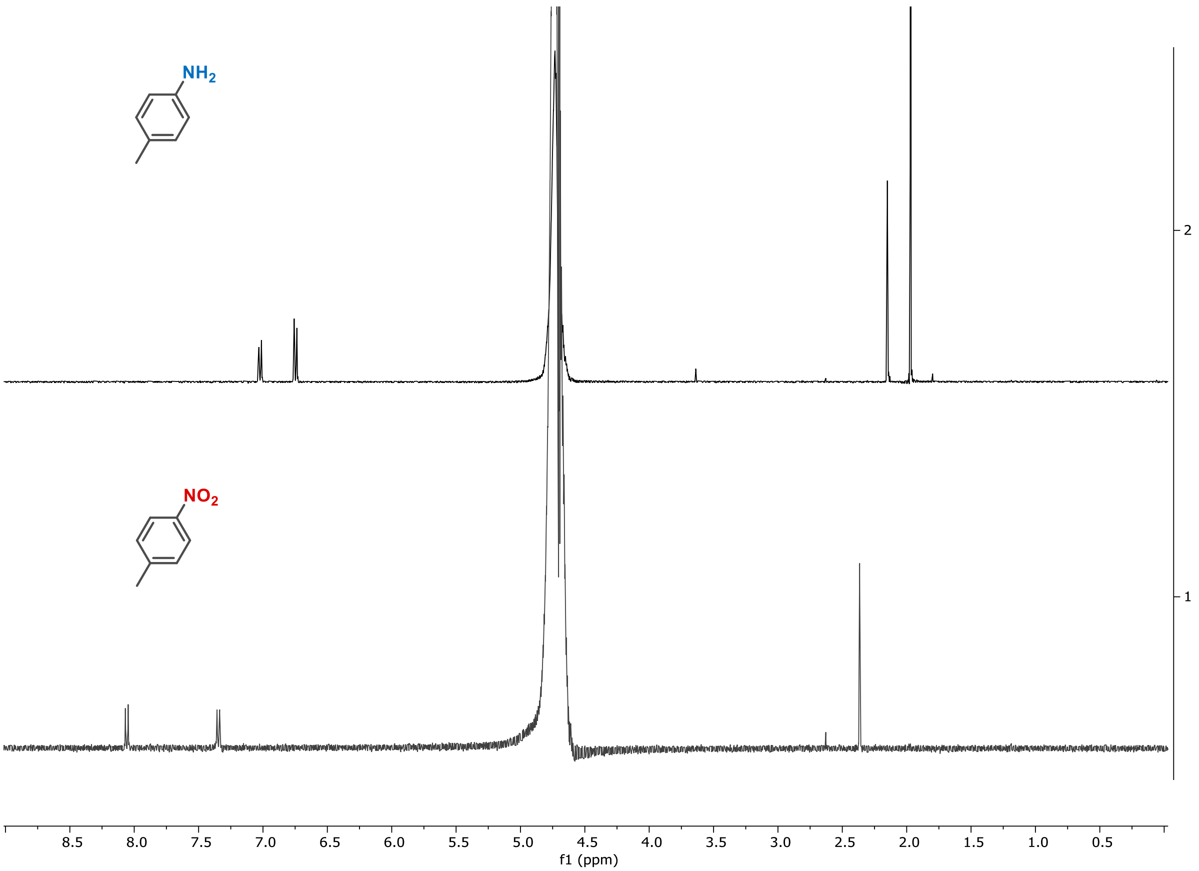

Supplementary Figure 9. ^{1}H-NMR spectra (400 MHz, 298 K, 10% D_2O in PB, 50 mM, pH 6.0) of substrate **4** and reaction mixture after 24 hours of reaction time.

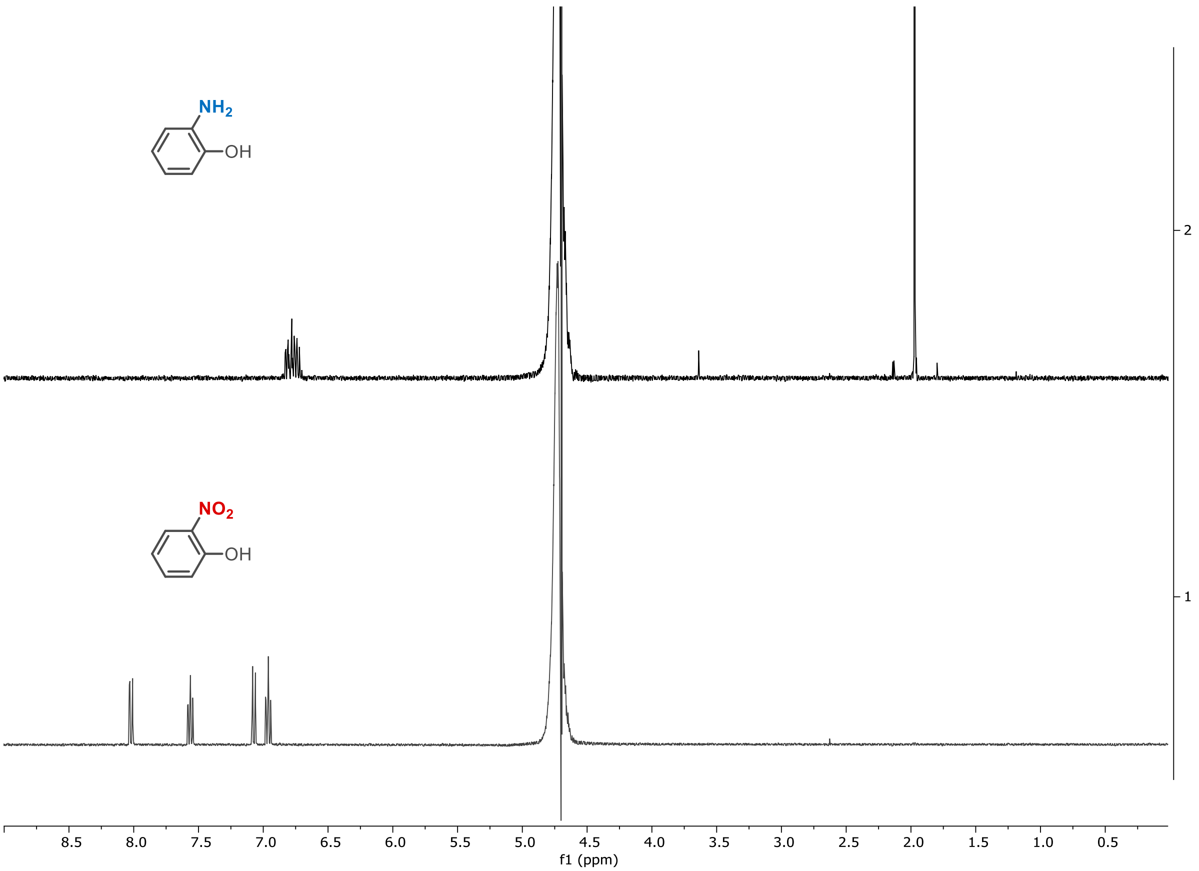

Supplementary Figure 10. ^{1}H-NMR spectra (400 MHz, 298 K, 10% D_2O in PB, 50 mM, pH 6.0) of substrate **5** and reaction mixture after 24 hours of reaction time.

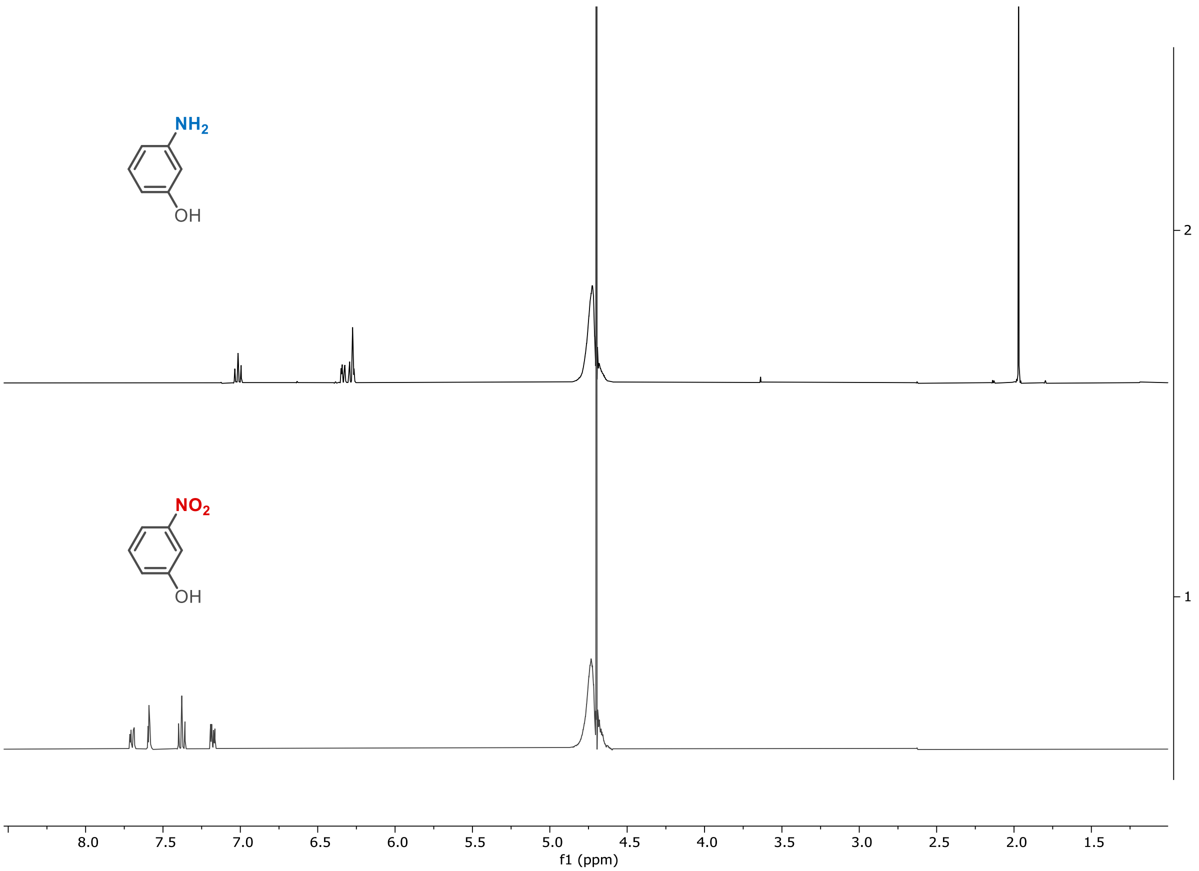

Supplementary Figure 11. ^{1}H-NMR spectra (400 MHz, 298 K, 10% D_2O in PB, 50 mM, pH 6.0) of substrate **6** and reaction mixture after 48 hours of reaction time.

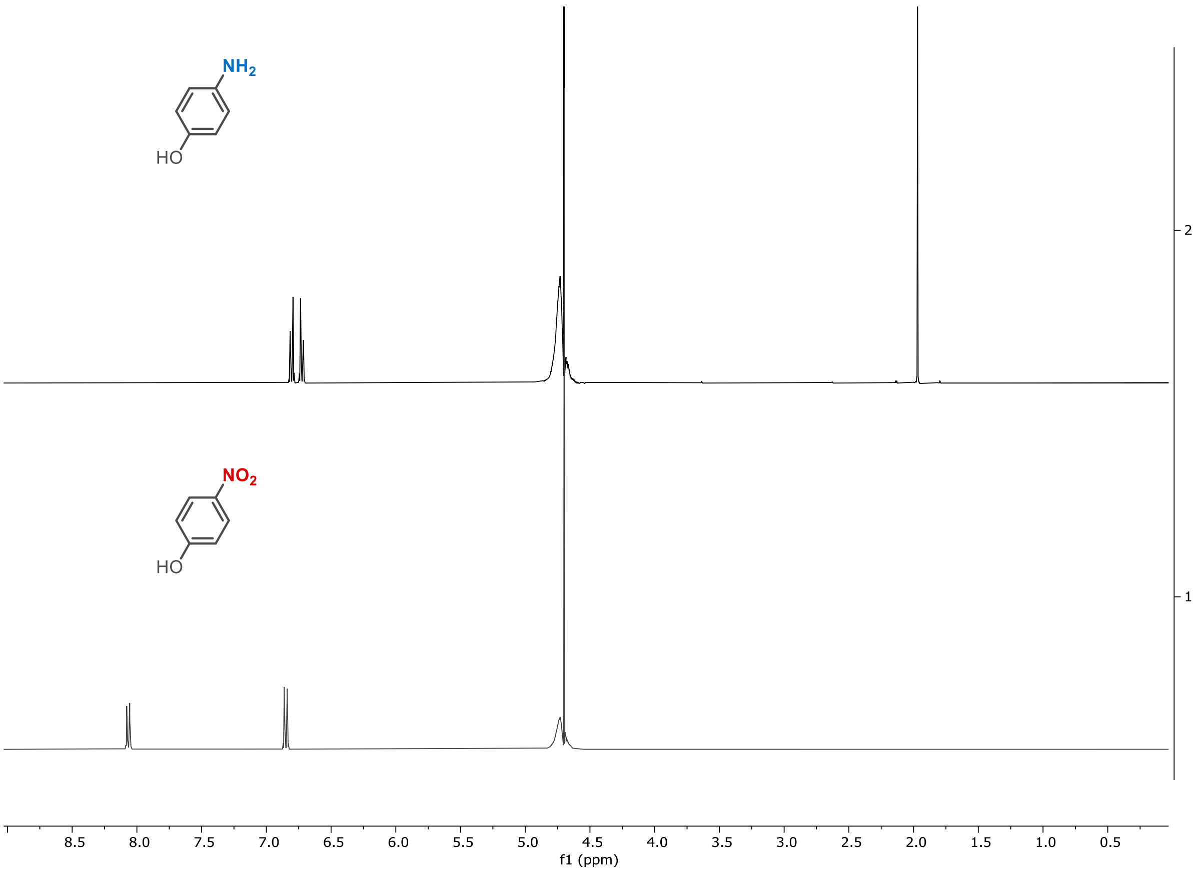

Supplementary Figure 12. ^{1}H-NMR spectra (400 MHz, 298 K, 10% D_2O in PB, 50 mM, pH 6.0) of substrate **7** and reaction mixture after 24 hours of reaction time.

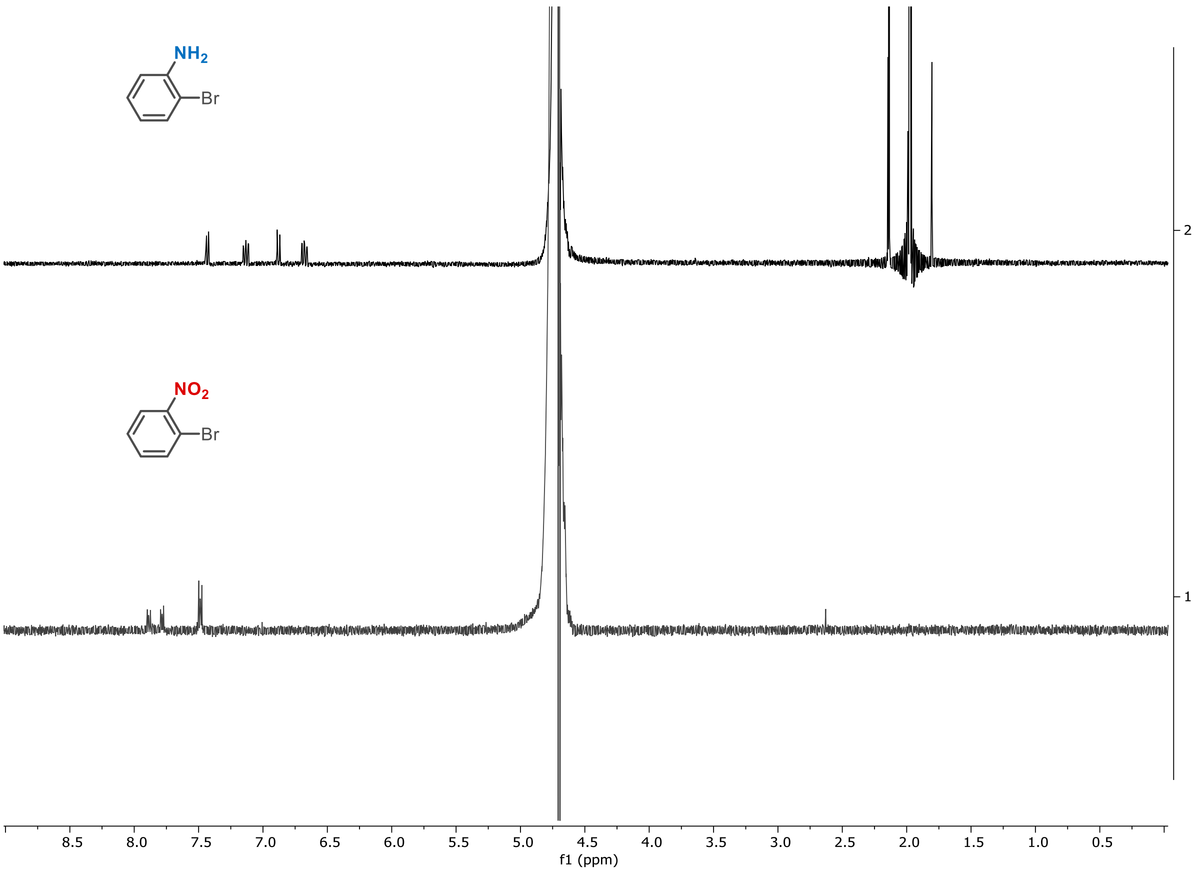

Supplementary Figure 13. ^{1}H-NMR spectra (400 MHz, 298 K, 10% D_2O in PB, 50 mM, pH 6.0) of substrate **8** and reaction mixture after 24 hours of reaction time.

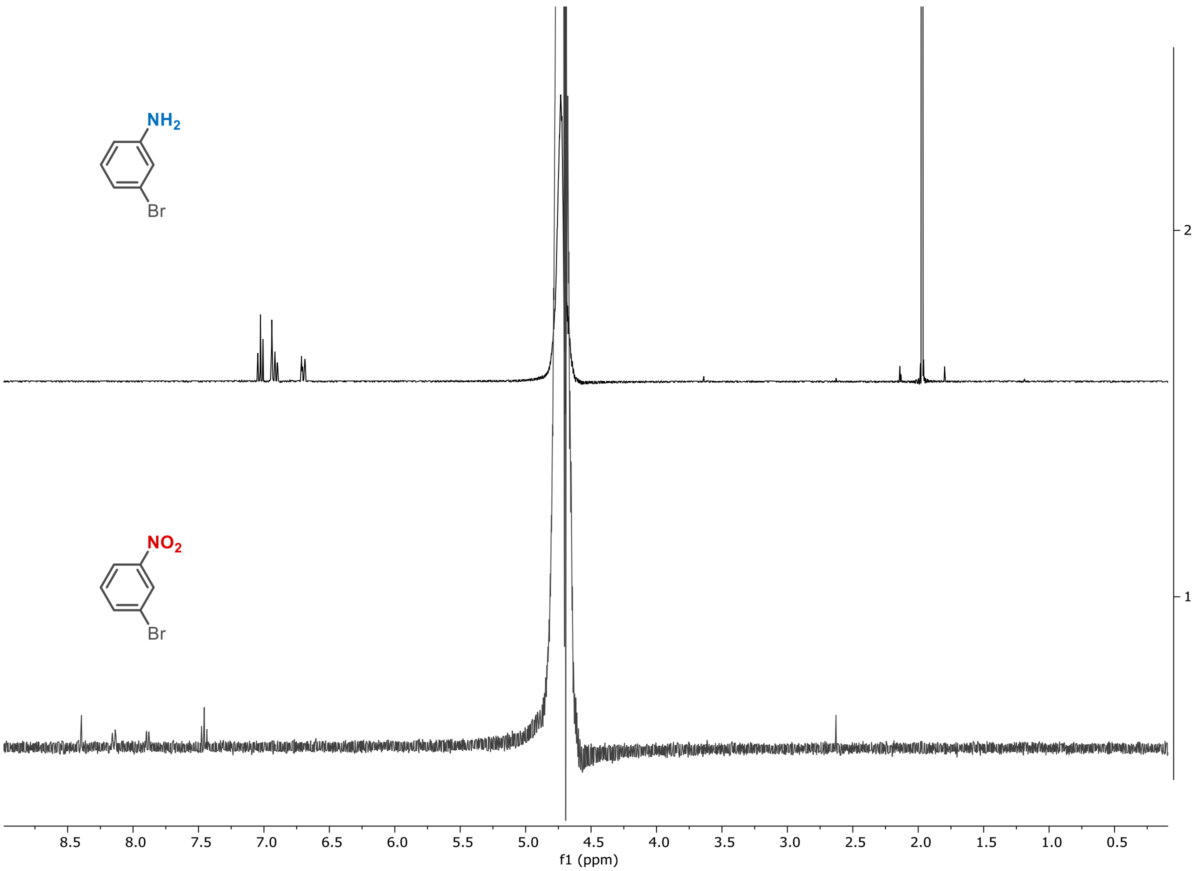

Supplementary Figure 14. ^{1}H-NMR spectra (400 MHz, 298 K, 10% D_2O in PB, 50 mM, pH 6.0) of substrate **9** and reaction mixture after 24 hours of reaction time.

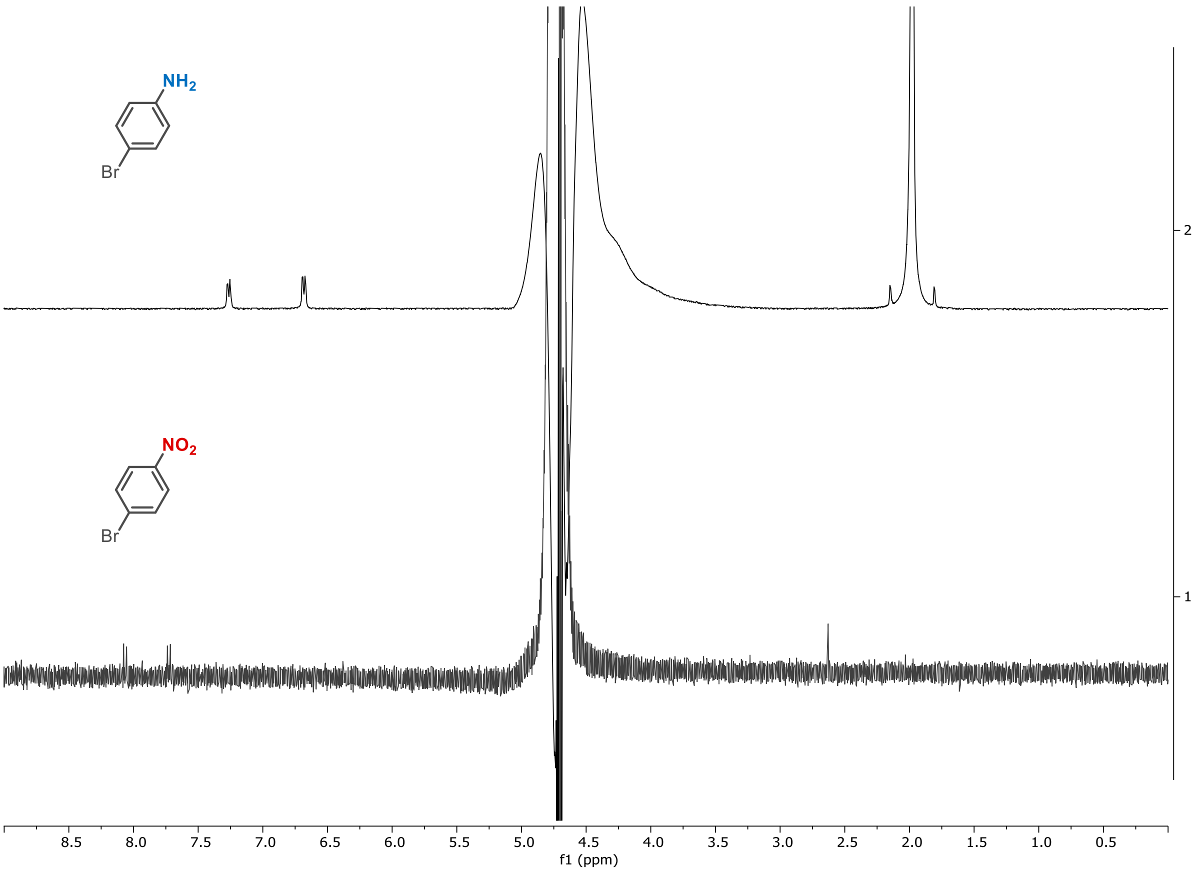

Supplementary Figure 15. ^{1}H-NMR spectra (400 MHz, 298 K, 10% D_2O in PB, 50 mM, pH 6.0) of substrate **10** and reaction mixture after 24 hours of reaction time.

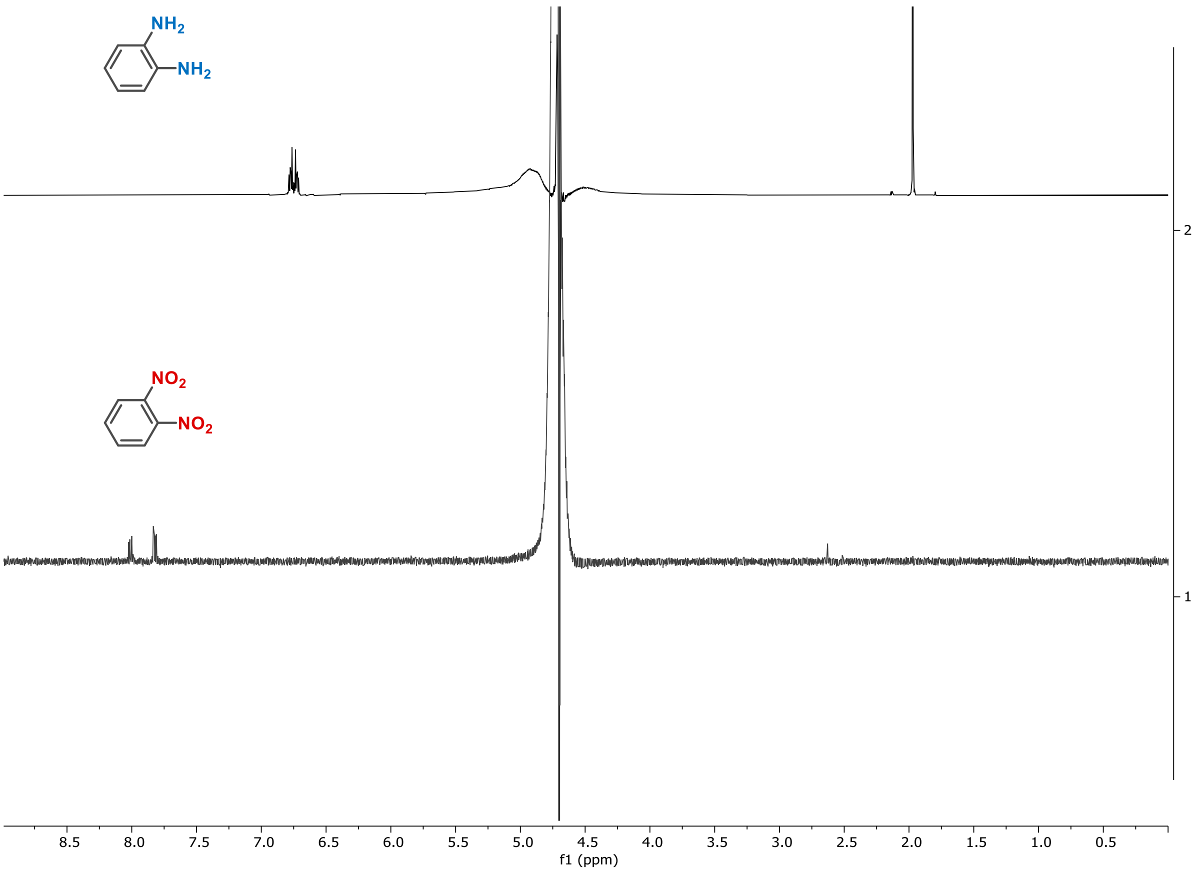

Supplementary Figure 16. ^{1}H-NMR spectra (400 MHz, 298 K, 10% D_2O in PB, 50 mM, pH 6.0) of substrate **11** and reaction mixture after 72 hours of reaction time.

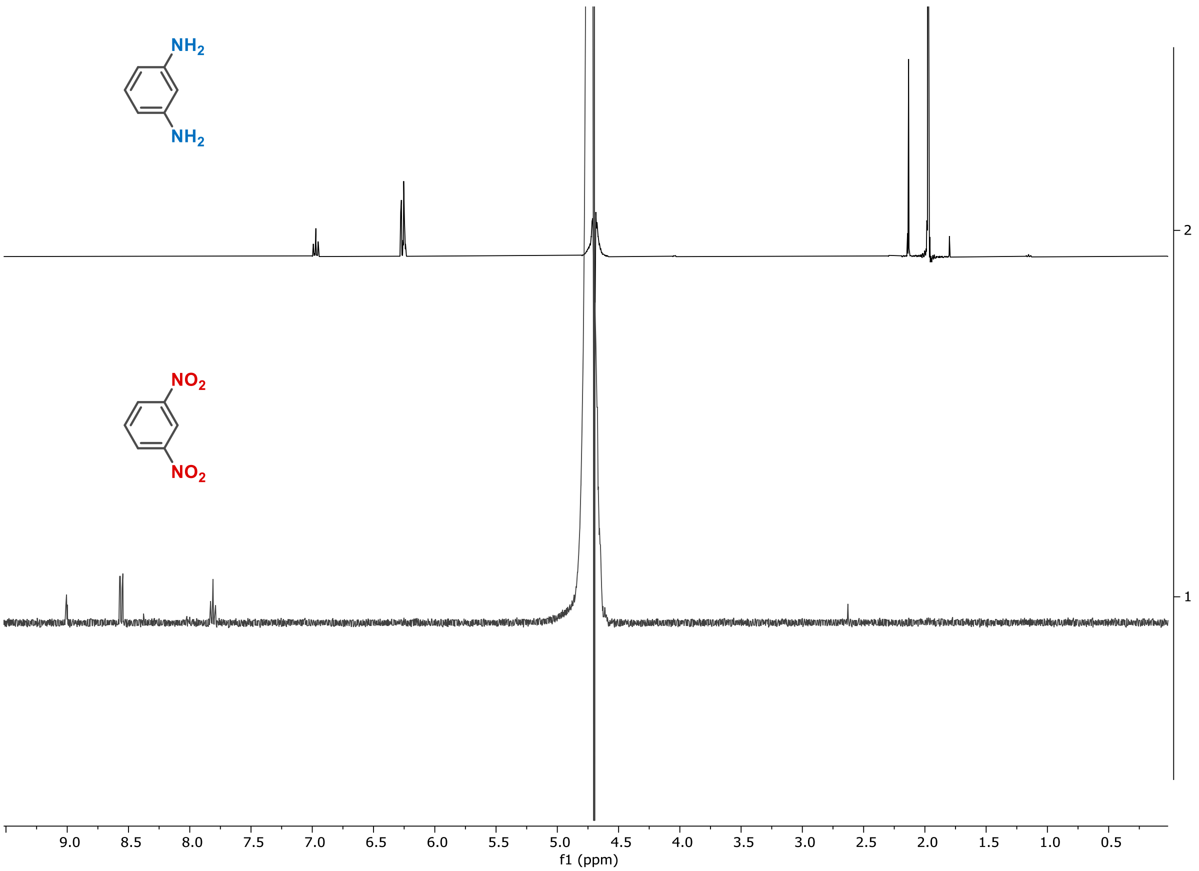

Supplementary Figure 17. ^{1}H-NMR spectra (400 MHz, 298 K, 10% D_2O in PB, 50 mM, pH 6.0) of substrate **12** and reaction mixture after 48 hours of reaction time.

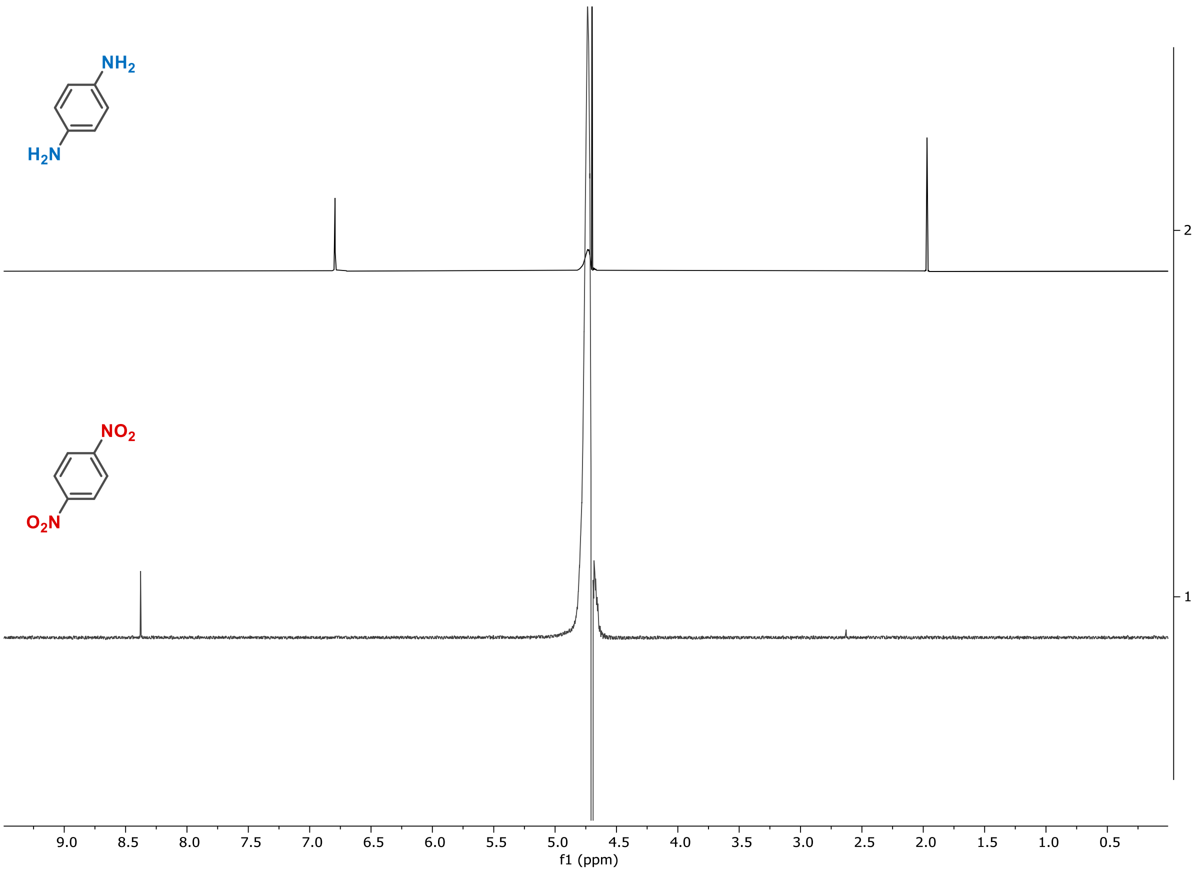

Supplementary Figure 18. ^{1}H-NMR spectra (400 MHz, 298 K, 10% D_2O in PB, 50 mM, pH 6.0) of substrate **13** and reaction mixture after 24 hours of reaction time.

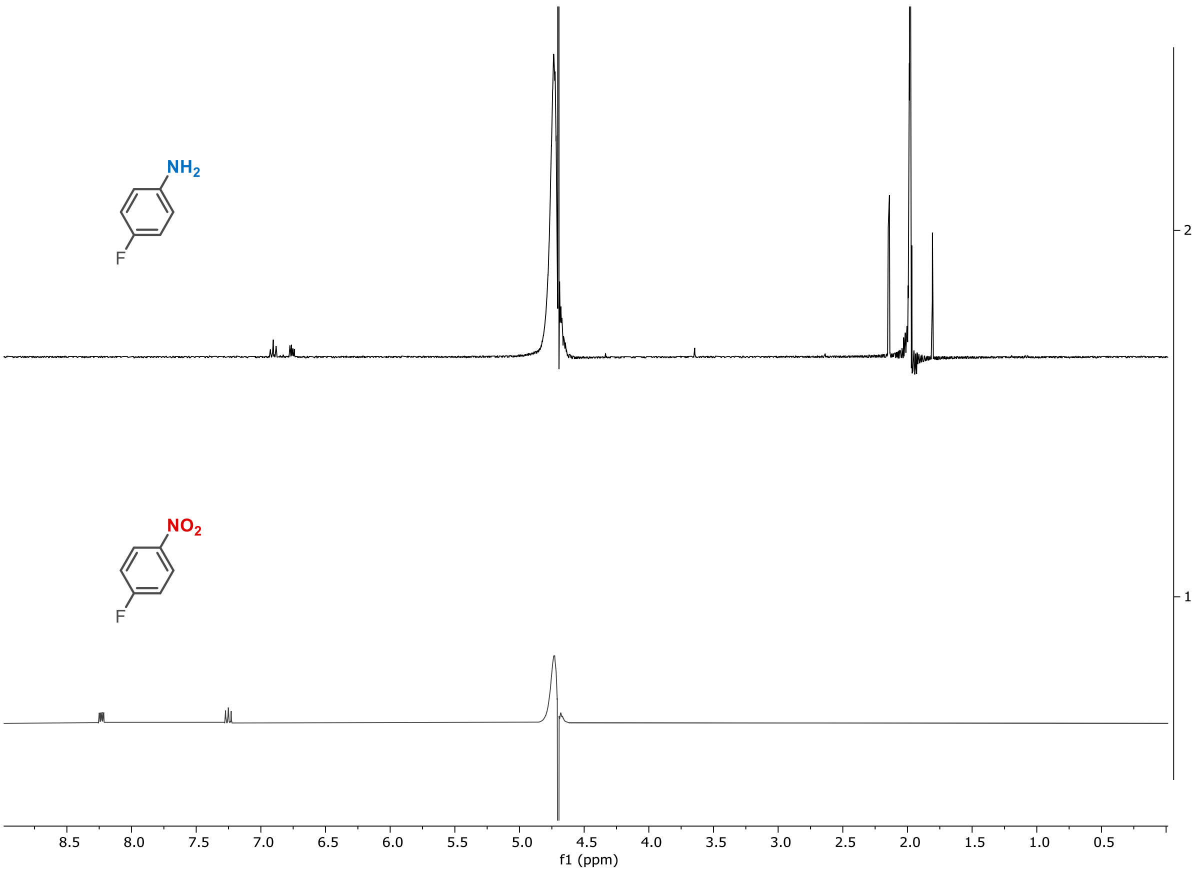

Supplementary Figure 19. ^{1}H-NMR spectra (400 MHz, 298 K, 10% D_2O in PB, 50 mM, pH 6.0) of substrate **14** and reaction mixture after 24 hours of reaction time.

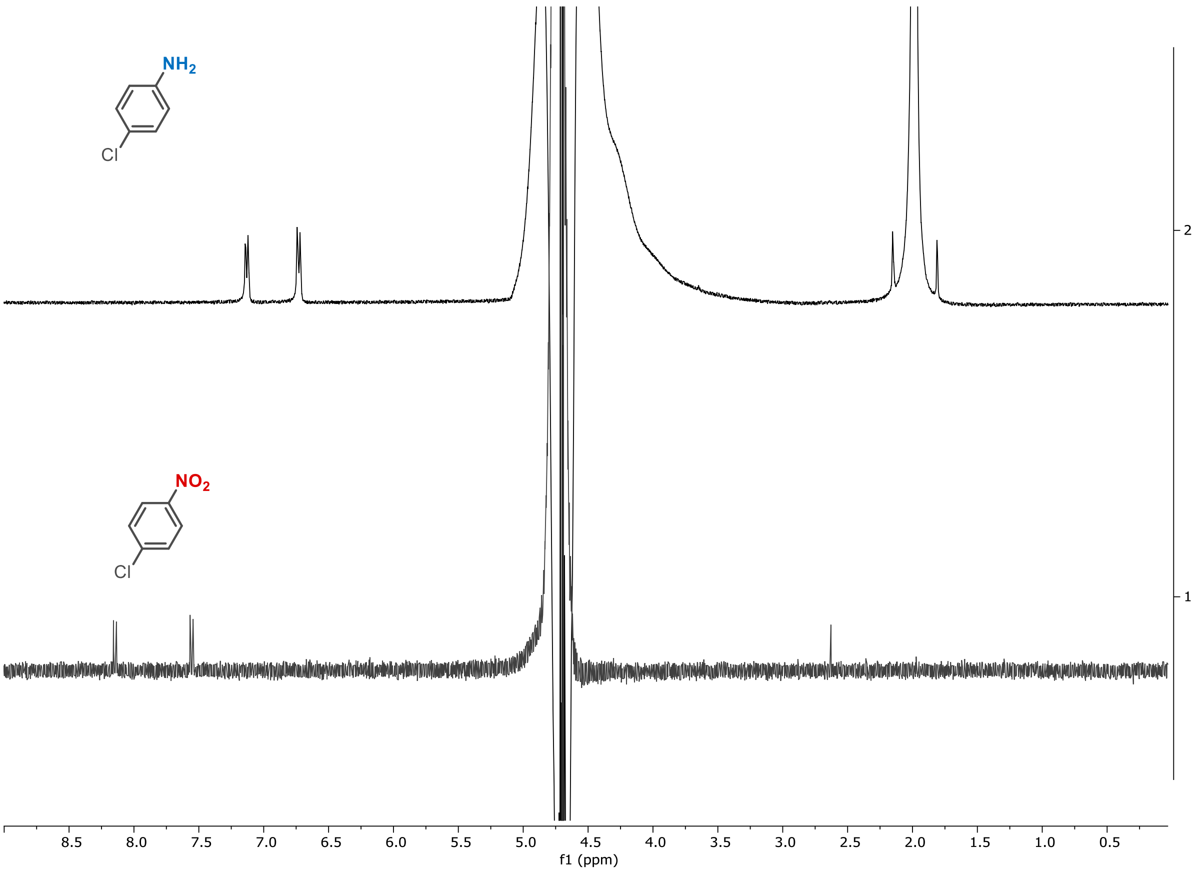

Supplementary Figure 20. ^{1}H-NMR spectra (400 MHz, 298 K, 10% D_2O in PB, 50 mM, pH 6.0) of substrate **15** and reaction mixture after 24 hours of reaction time.

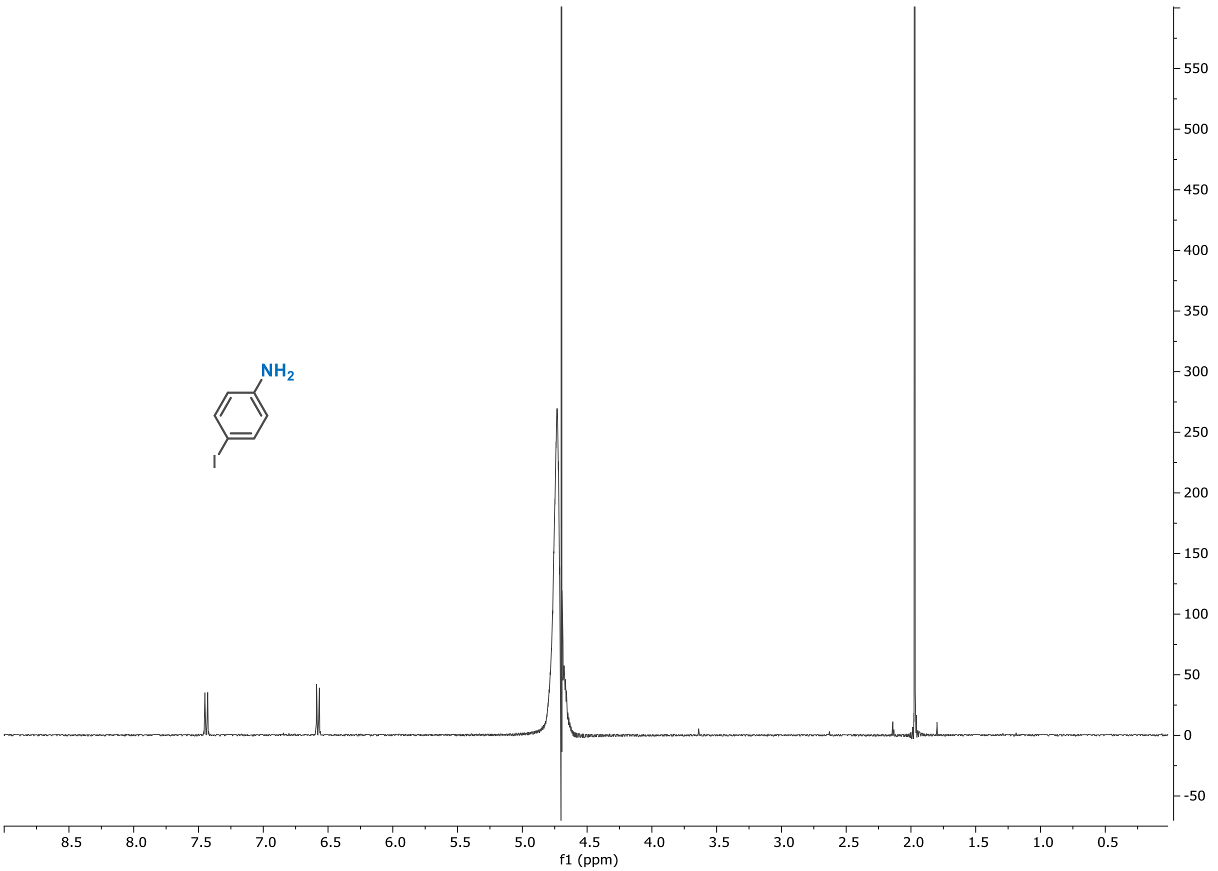

Supplementary Figure 21. ^{1}H-NMR spectrum (400 MHz, 298 K, 10% D_2O in PB, 50 mM, pH 6.0) the reaction mixture after 24 hours of reaction with substrate **16**. Substrate **16** does not dissolve in buffer.

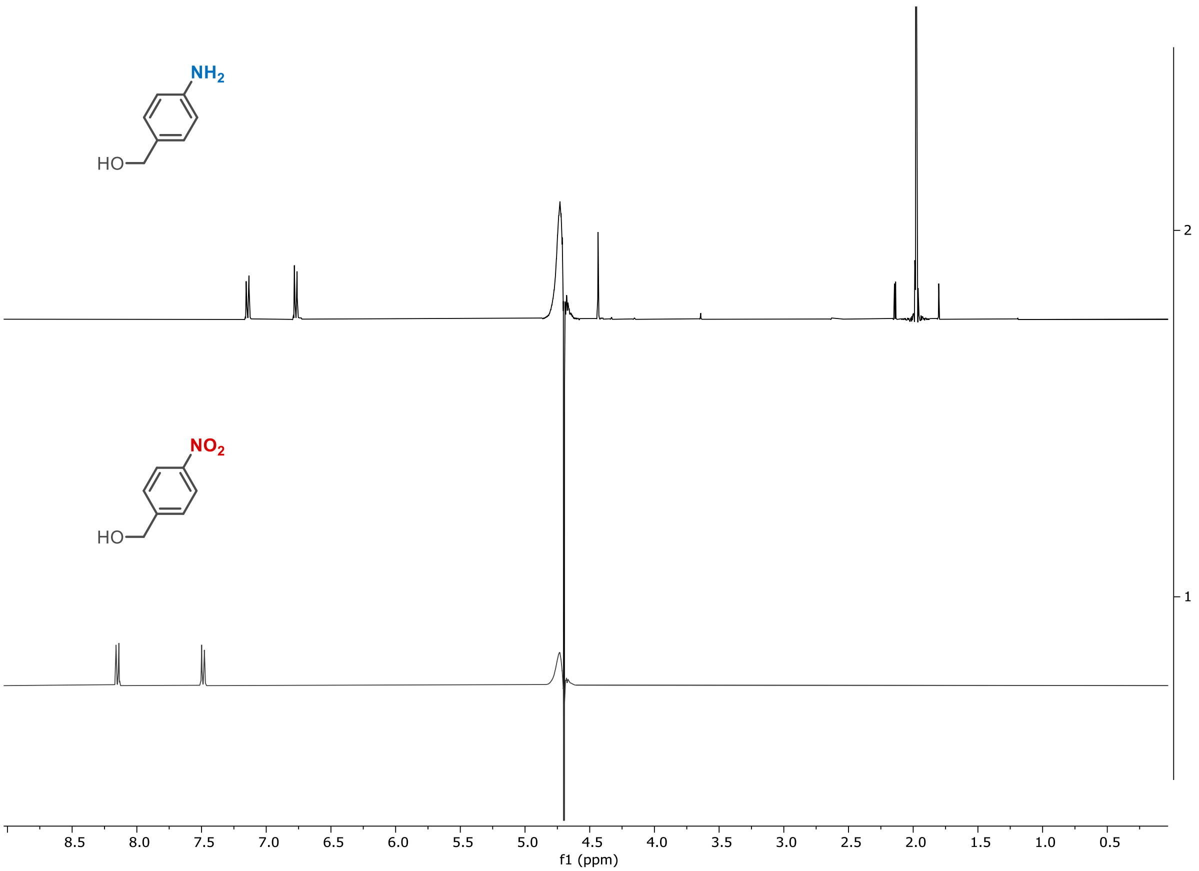

Supplementary Figure 22. ^{1}H-NMR spectra (400 MHz, 298 K, 10% D_2O in PB, 50 mM, pH 6.0) of substrate **17** and reaction mixture after 48 hours of reaction time.

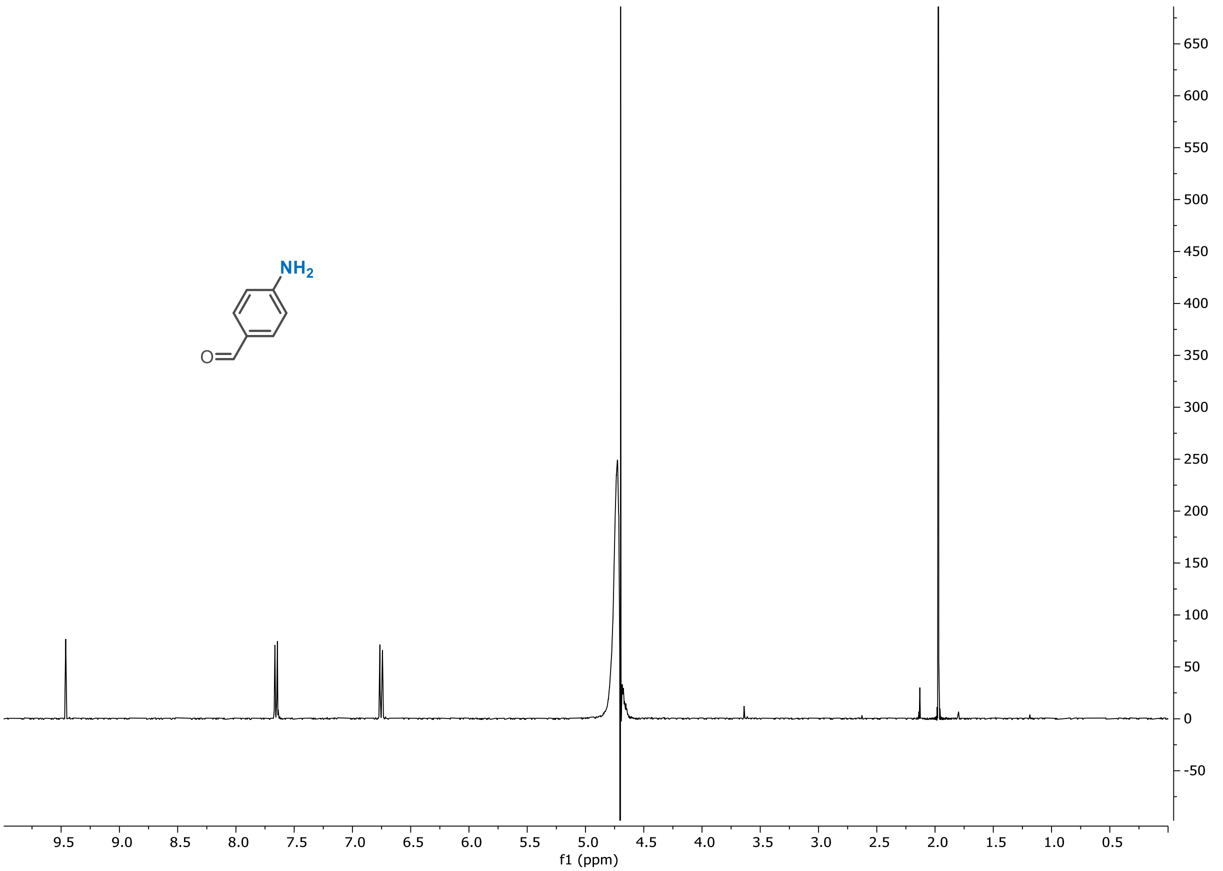

Supplementary Figure 23. ^{1}H-NMR spectra (400 MHz, 298 K, 10% D_2O in PB, 50 mM, pH 6.0) of the reaction mixture after 72 hours of reaction with substrate **18**. Substrate **18** does not dissolve in buffer.

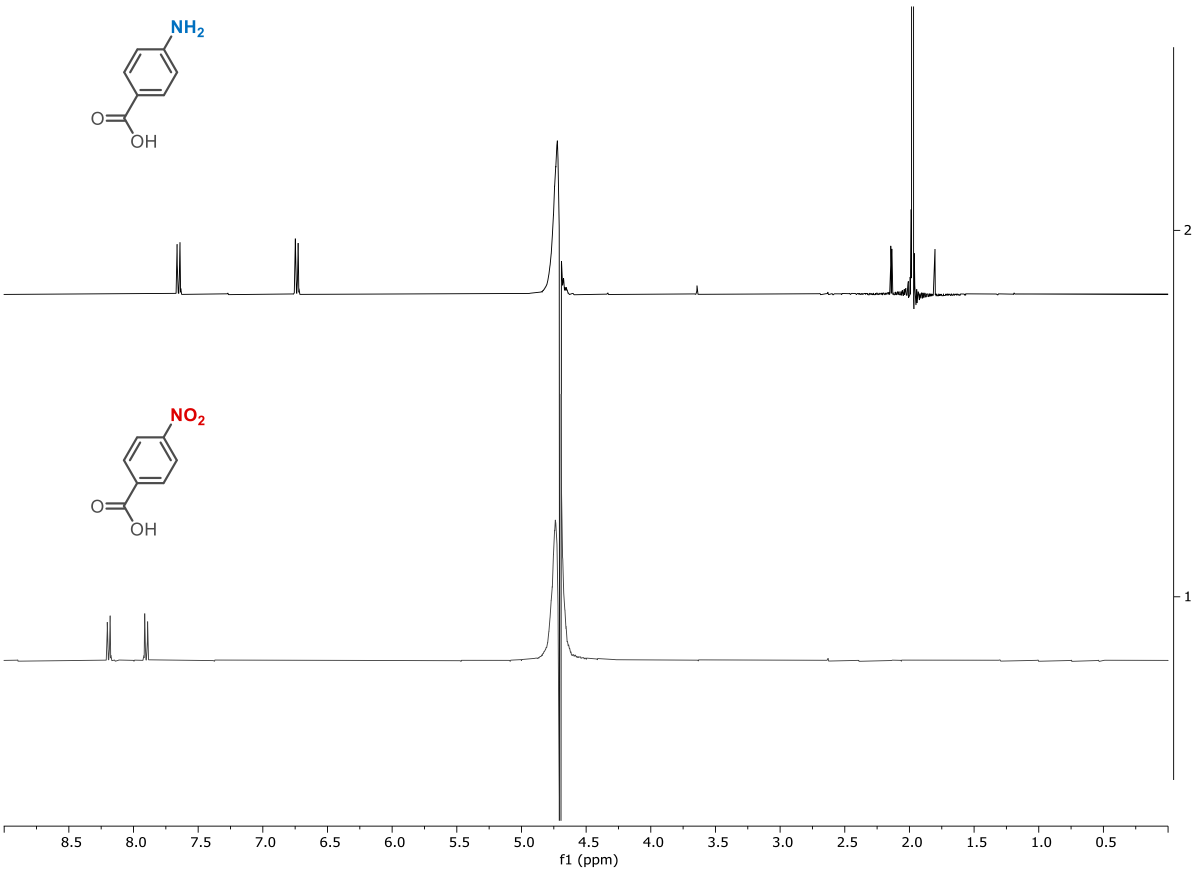

Supplementary Figure 24. ^{1}H-NMR spectra (400 MHz, 298 K, 10% D_2O in PB, 50 mM, pH 6.0) of substrate **19** and reaction mixture after 48 hours of reaction time.

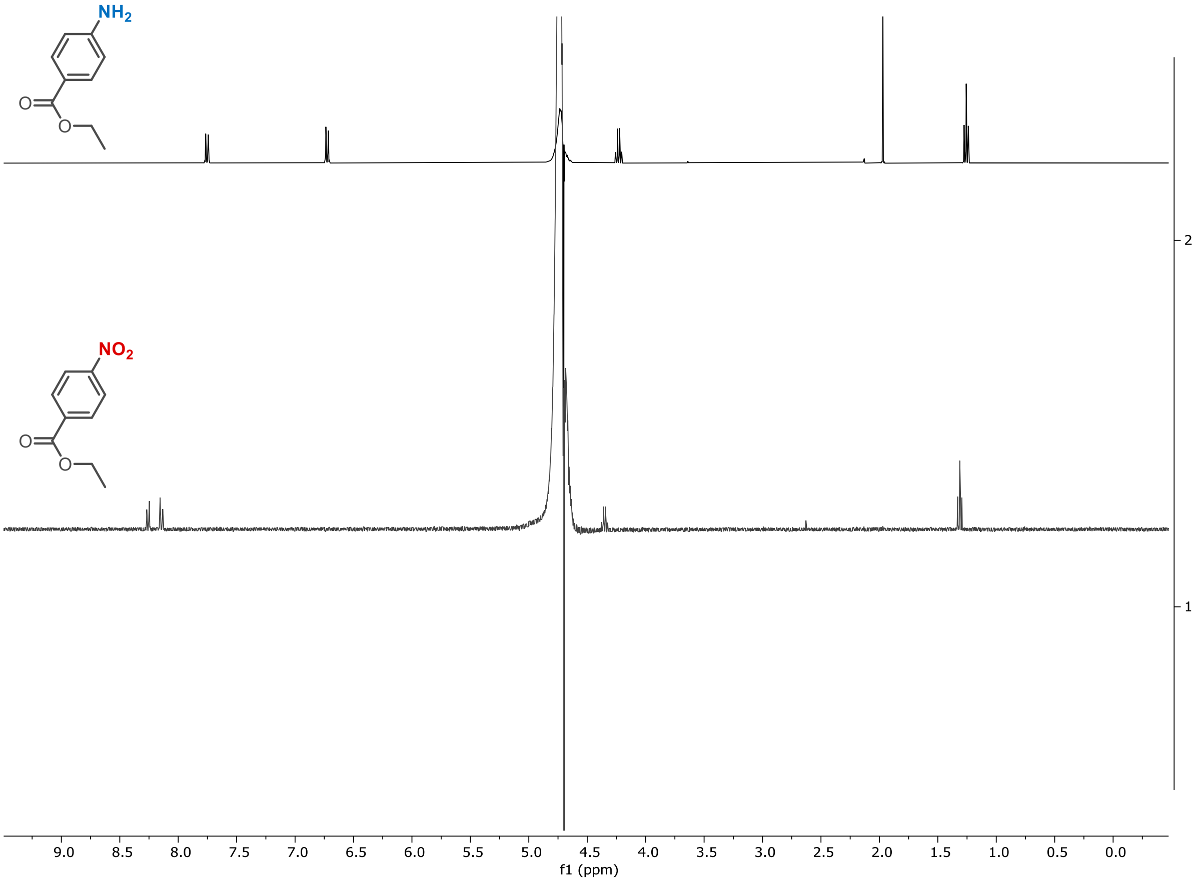

Supplementary Figure 25. ^{1}H-NMR spectra (400 MHz, 298 K, 10% D_2O in PB, 50 mM, pH 6.0) of substrate **20** and reaction mixture after 48 hours of reaction time.

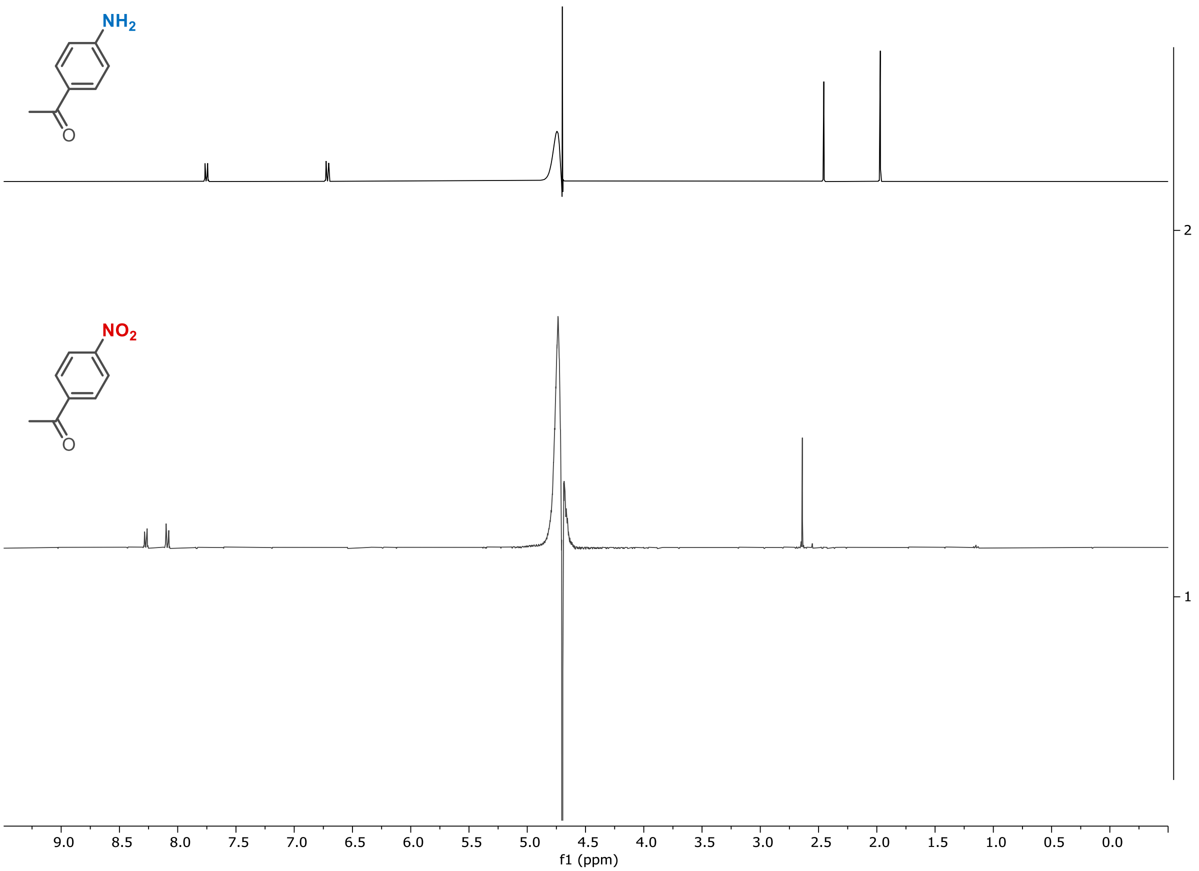

Supplementary Figure 26. ^{1}H-NMR spectra (400 MHz, 298 K, 10% D_2O in PB, 50 mM, pH 6.0) of substrate **21** and reaction mixture after 48 hours of reaction time.

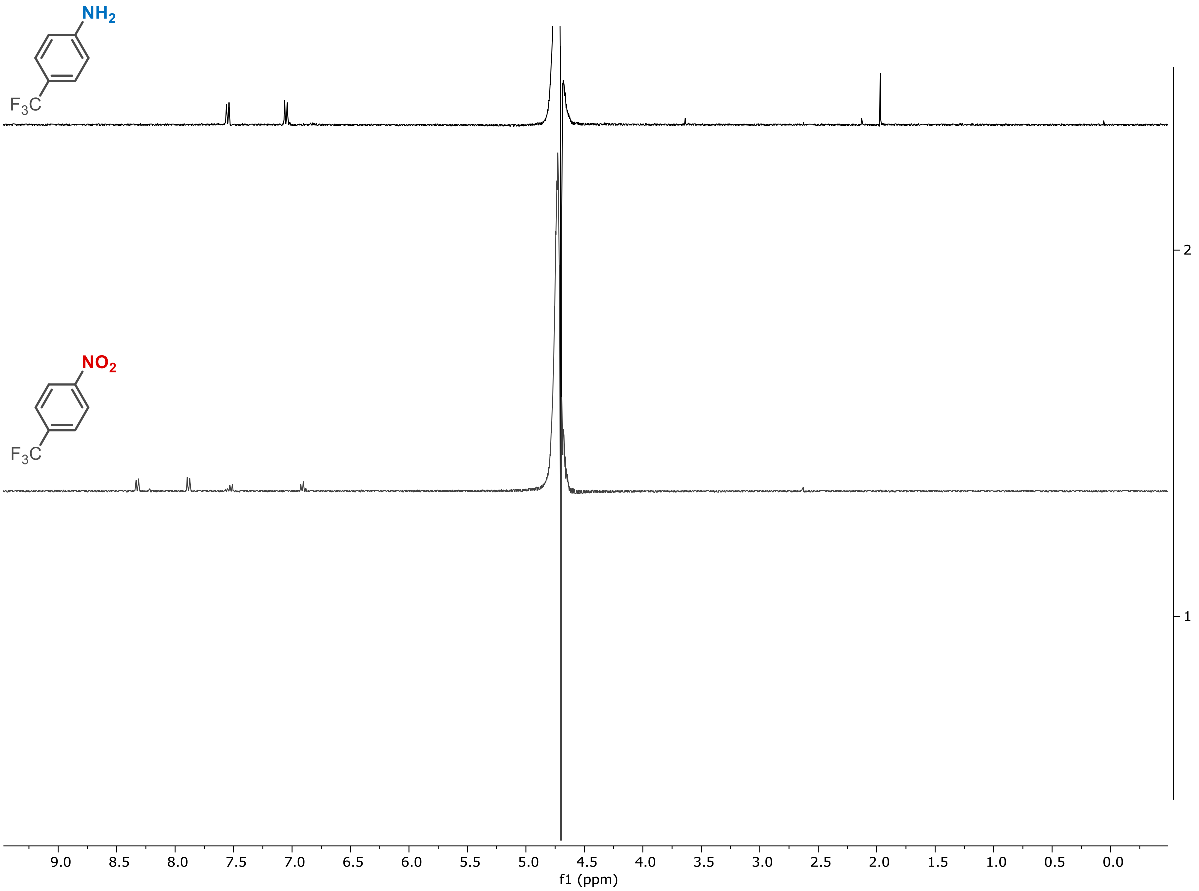

Supplementary Figure 27. ^{1}H-NMR spectra (400 MHz, 298 K, 10% D_2O in PB, 50 mM, pH 6.0) of substrate **22** and reaction mixture after 72 hours of reaction time.

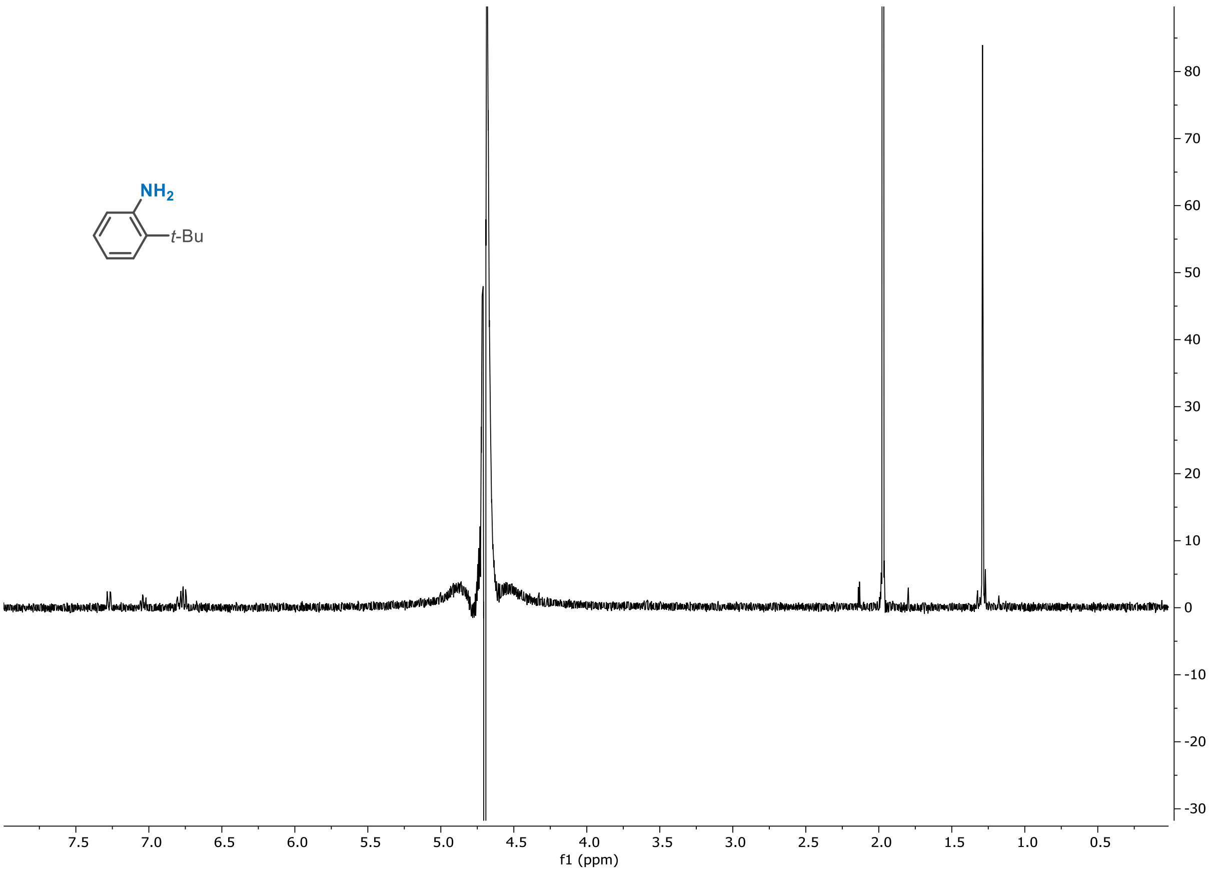

Supplementary Figure 28. ^{1}H-NMR spectra (400 MHz, 298 K, 10% D_2O in PB, 50 mM, pH 6.0) of the reaction mixture after 72 hours of reaction with substrate **23**. Substrate **23** does not dissolve in buffer.

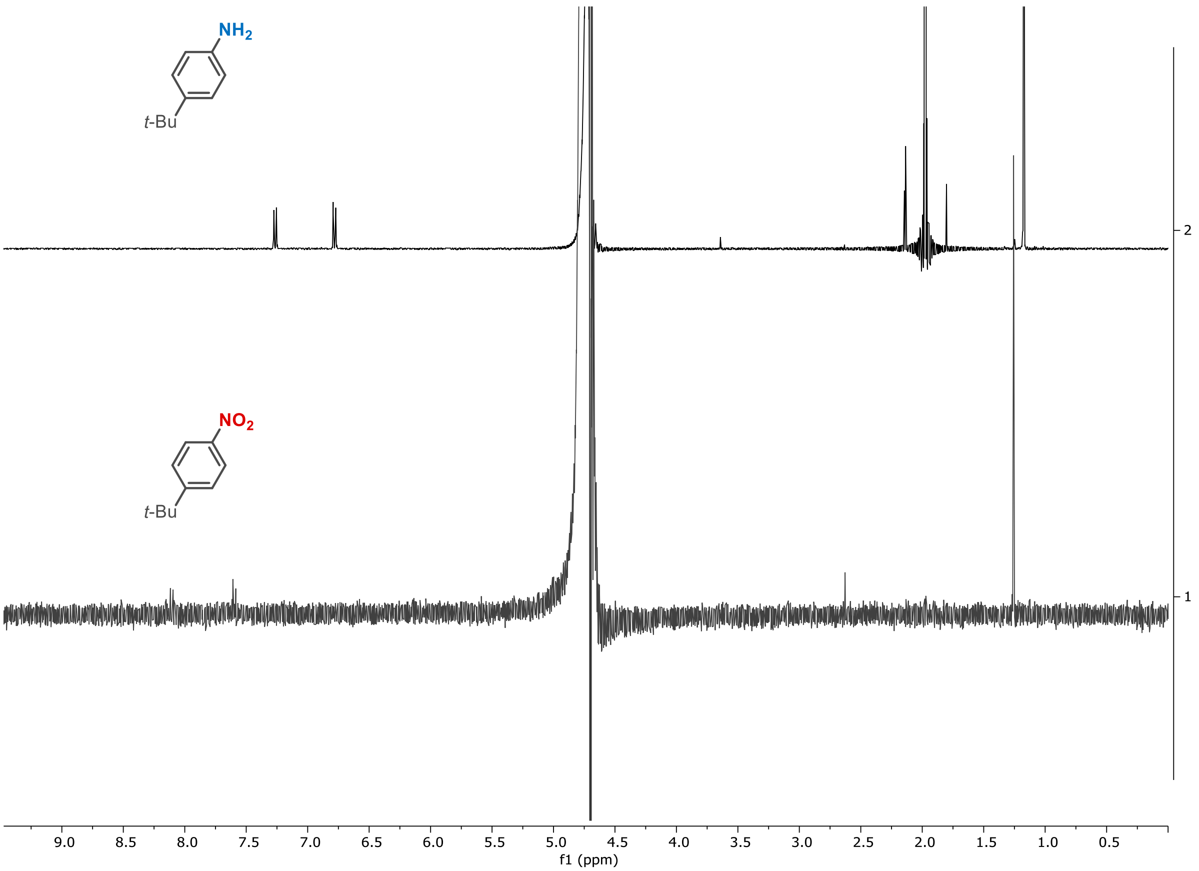

Supplementary Figure 29. ^{1}H-NMR spectra (400 MHz, 298 K, 10% D_2O in PB, 50 mM, pH 6.0) of substrate **24** and reaction mixture after 24 hours of reaction time. Substrate **24** has low solubility in water.

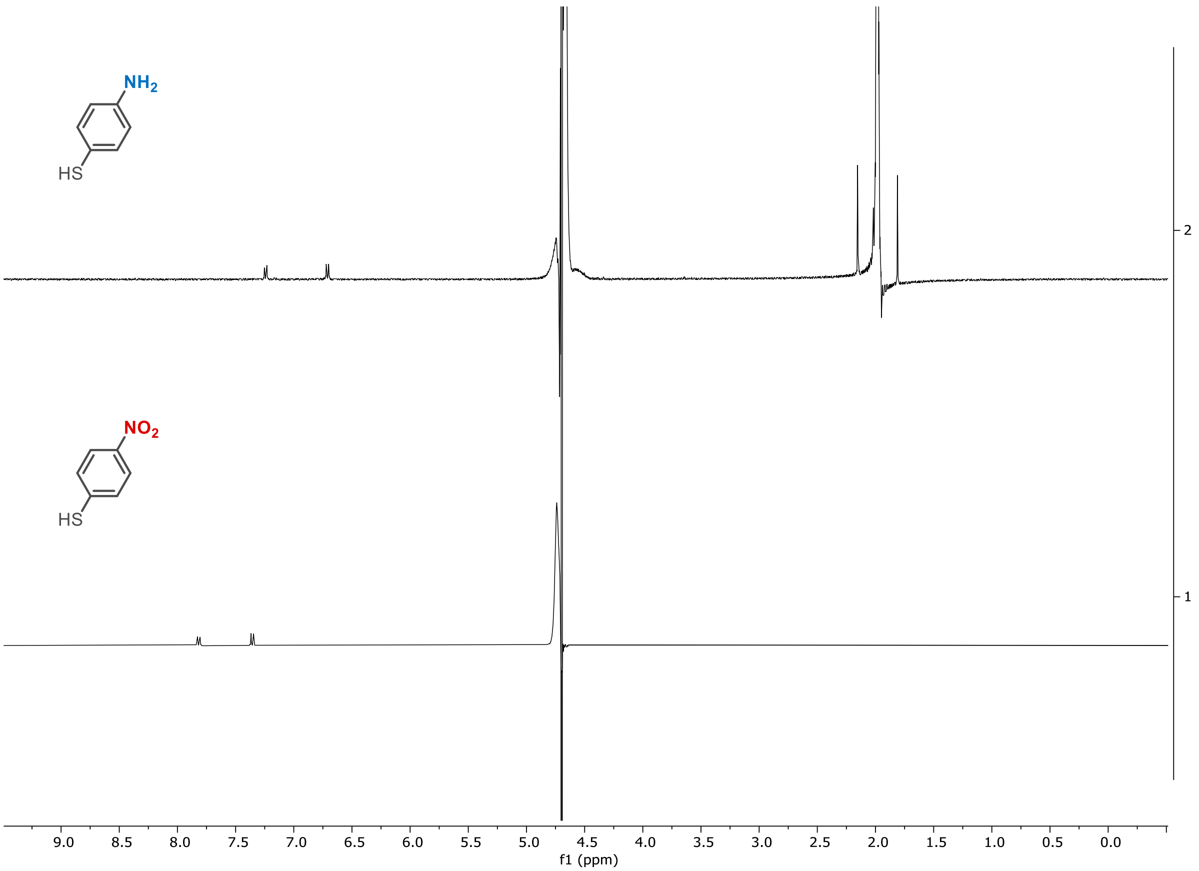

Supplementary Figure 30. ^{1}H-NMR spectra (400 MHz, 298 K, 10% D_2O in PB, 50 mM, pH 6.0) of substrate **25** and reaction mixture after 24 hours of reaction time.

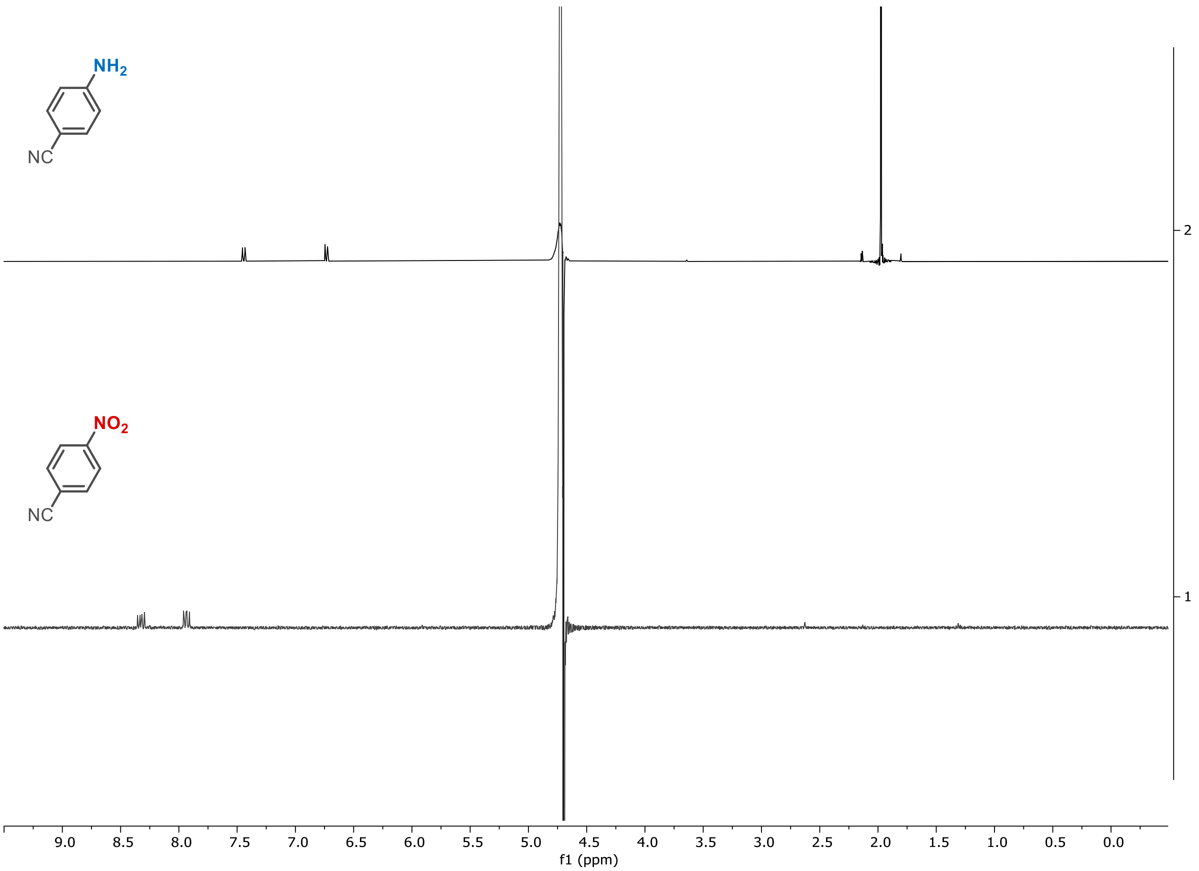

Supplementary Figure 31. ^{1}H-NMR spectra (400 MHz, 298 K, 10% D_2O in PB, 50 mM, pH 6.0) of substrate **26** and reaction mixture after 48 hours of reaction time.

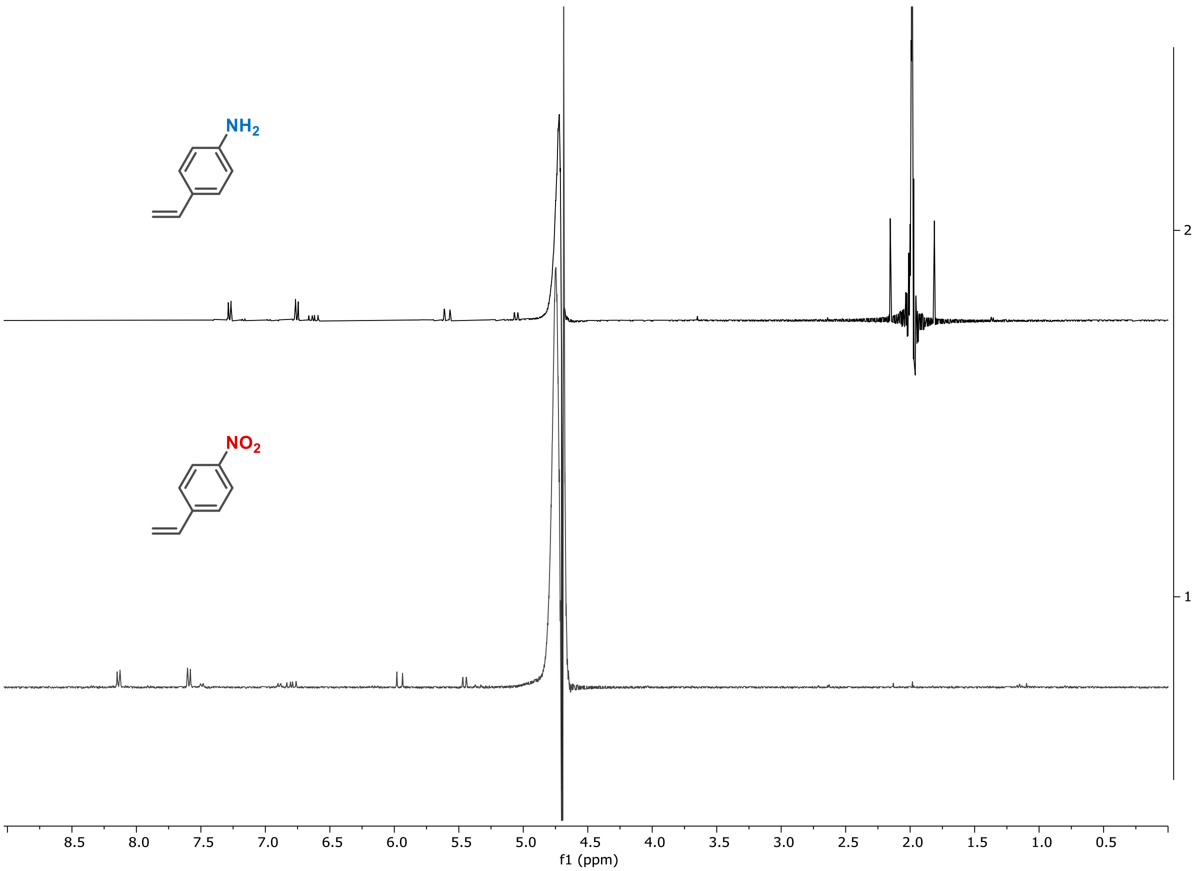

Supplementary Figure 32. ^{1}H-NMR spectra (400 MHz, 298 K, 10% D_2O in PB, 50 mM, pH 6.0) of substrate **27** and reaction mixture after 24 hours of reaction time.

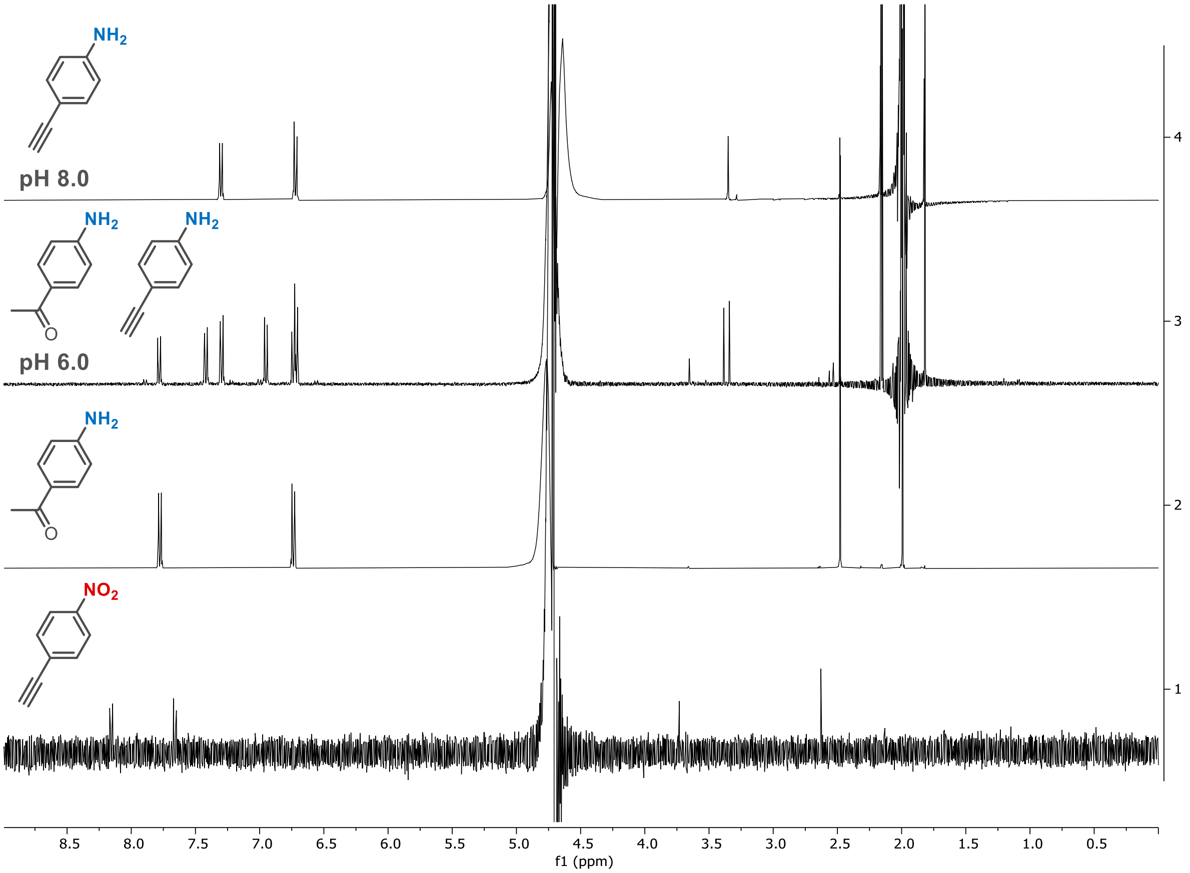

Supplementary Figure 33. ^{1}H-NMR spectra (400 MHz, 298 K, 10% D_2O in PB, 50 mM, pH 6.0 or 8.0) of substrate **28** and reaction mixture after 24 hours of reaction time. ^{1}H-NMR spectrum of the reaction mixture at pH 6.0 indicates formation of *p*-aminoacetophenone as a product of hydrolysis side reaction.

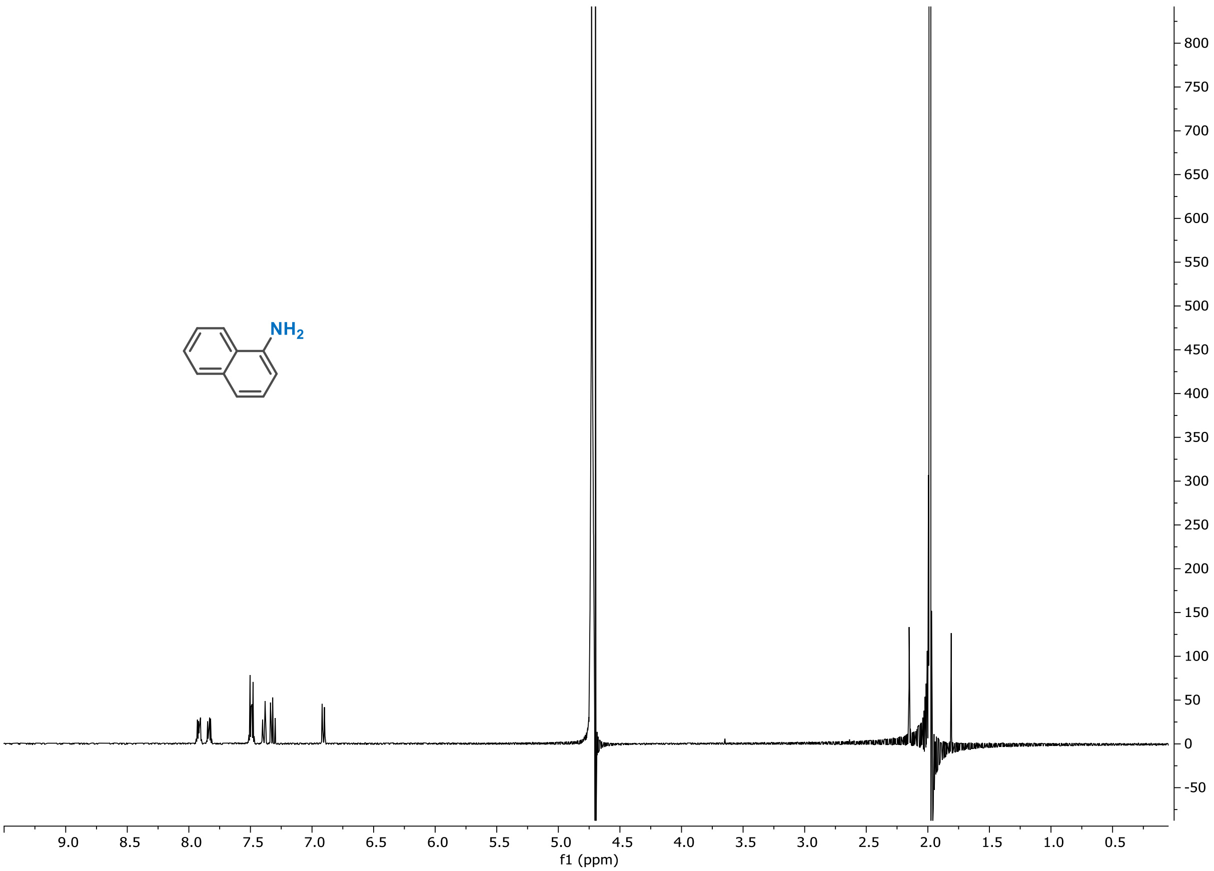

Supplementary Figure 34. ^{1}H-NMR spectra (400 MHz, 298 K, 10% D_2O in PB, 50 mM, pH 6.0) of the reaction mixture after 24 hours of reaction with substrate **29**. Substrate **29** does not dissolve in buffer.

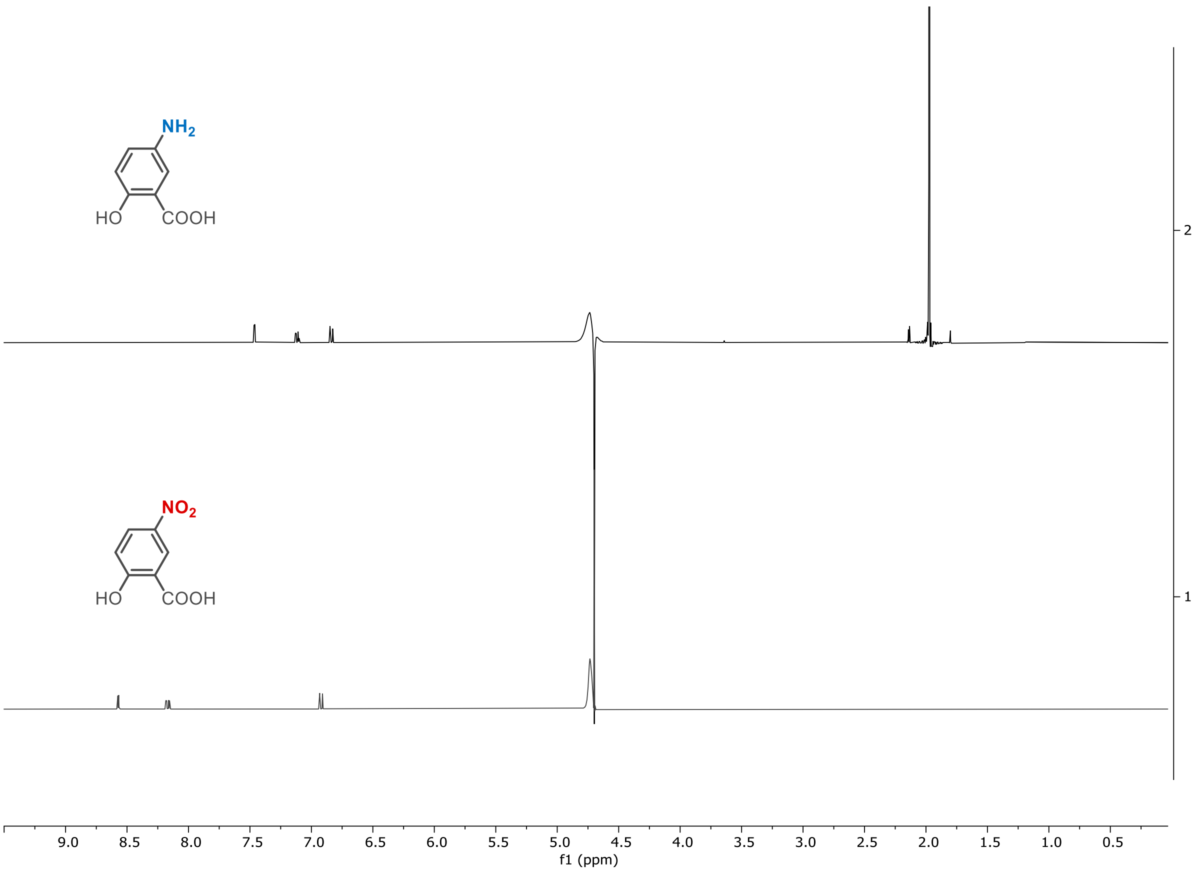

Supplementary Figure 35. ^{1}H-NMR spectra (400 MHz, 298 K, 10% D_2O in PB, 50 mM, pH 6.0) of substrate **30** and reaction mixture after 48 hours of reaction time.

V. Preparative Hydrogenation Experiments

Reaction set-up was performed as described in Section IV with the optimised conditions (Supplementary Table 2). Reaction completion was confirmed by ^{1}H-NMR spectroscopy. After reaction completion, the reaction mixture was centrifuged for 5 min at 14100 × g. The supernatant was then transferred into a glass vial, extracted with Et_2O (3×2 mL, work-up **A** for **1a**–**4a**, **23a**–**24a**), EtOAc (3×2 mL, work-up **B** for **5a**–**10a**, **14a**–**17a**, **19a**–**22a**, **26a**–**29a**), or directly concentrated under reduced pressure and suspended in chloroform (work-up **C** for **11a**–**13a**) or EtOAc (work-up **D** for **30a**), dried with Na_2SO_4, filtered, and concentrated *in vacuo*. The products were then analysed by ^{1}H-NMR spectroscopy. Products **18a** and **25a** were not isolated from the reaction mixtures due to their instability on air. ^{1}H-NMR-based yields were detected for both compounds as described in Section I.

Supplementary Table 2. Optimised reaction conditions for hydrogenation reactions with substrates **1**–**30**, 10 mM concentration of substrate, 2 mL scale in PB (50 mM, pH as indicated).

Substrate	Cat. loading[1]	MeCN (%)[2]	pH	Time (h)[3]	Work-up	Yield (%)[4]
1	**X**	0	6.0	12	A	72
2	**X**	0	6.0	24	A	81
3	**X**	0	6.0	24	A	87
4	**X**	10	6.0	24	A	83
5	**X**	0	6.0	24	B	80
6	**X**	0	6.0	48	B	82
7	**X**	0	6.0	24	B	80
8	2·**X**	10	6.0	24	B	89
9	**X**	10	6.0	24	B	82
10	**X**	10	6.0	24	B	81
11	**X**	10	6.0	72	C	78
12	2·**X**	10	6.0	48	C	82
13	**X**	10	6.0	24	C	82
14	2·**X**	0	6.0	24	B	87
15	**X**	10	6.0	24	B	92
16	**X**	10	6.0	24	B	86
17	2·**X**	10	6.0	48	B	82
18	2·**X**	10	6.0	72	–	91[a]
19	2·**X**	0	6.0	48	B	91
20	2·**X**	10	6.0	48	B	93
21	**X**	10	6.0	48	B	90
22	**X**	10	6.0	72	B	85
23	2·**X**	10	6.0	72	A	86
24	2·**X**	10	6.0	24	A	89
25	**X**	0	6.0	24	–	78[a]
26	2·**X**	10	6.0	48	B	83
27	4·**X**	10	6.0	24	B	83
28	2·**X**	10	**8.0**	24	B	82
29	**X**	10	6.0	24	B	96
30	2·**X**	10	6.0	48	D	88

[1]**X** is a standard catalyst loading of 1.32 mg of Hyd-1 per 1 mmol of substrate, C:Hyd-1 = 40:1 mass ratio; [2]MeCN used as a co-solvent, 0% or 10% v/v; [3]Time of full conversion of substrate to corresponding amine; [4]Isolated yields; [a]^{1}H-NMR yield determined using 4-nitrophenol as internal standard.

1a 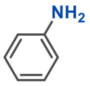

Aniline (**1a**). Colorless liquid, 1.33 mg (72%). ^{1}H-NMR (400 MHz, $CDCl_3$): δ = 7.22 – 7.12 (m, 2H), 6.80 – 6.73 (m, 1H), 6.73 – 6.66 (m, 2H), 3.63 (brs, 2H). The obtained data match those reported in literature.[3]

2a 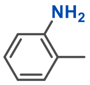

o-Toluidine (**2a**). Colorless liquid, 1.73 mg (81%). ^{1}H-NMR (400 MHz, $CDCl_3$): δ = 7.09 – 7.01 (m, 2H), 6.77 – 6.68 (m, 2H), 3.86 (brs, 2H), 2.19 (s, 3H). The obtained data match those reported in literature.[4]

3a 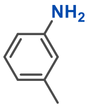

m-Toluidine (**3a**). Colorless liquid, 1.86 mg (87%). ^{1}H-NMR (400 MHz, $CDCl_3$): δ = 7.05 (t, J = 7.6 Hz, 1H), 6.65 – 6.57 (m, 1H), 6.57 – 6.49 (m, 2H), 3.64 (brs, 2H), 2.27 (s, 3H). The obtained data match those reported in literature.[4]

4a 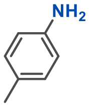

p-Toluidine (**4a**). Colorless solid, 1.77 mg (83%). ^{1}H-NMR (400 MHz, $CDCl_3$): δ = 6.99 – 6.94 (m, 2H), 6.63 (d, J = 8.3 Hz, 2H), 3.71 (brs, 2H), 2.24 (s, 3H). The obtained data match those reported in literature.[4]

5a 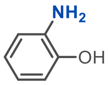

o-Aminophenol (**5a**). White solid, 1.76 mg (80%). ^{1}H-NMR (400 MHz, DMSO-d_6): δ = 8.90 (brs, 1H), 6.66 – 6.59 (m, 1H), 6.59 – 6.47 (m, 2H), 6.42 – 6.32 (m, 1H), 4.44 (brs, 2H). The obtained data match those reported in literature.[5]

6a 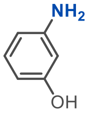

m-Aminophenol (**6a**). White solid, 1.80 mg (82%). ^{1}H-NMR (400 MHz, $CDCl_3$): δ = 7.03 (t, J = 8.0 Hz, 1H), 6.42 – 6.10 (m, 3H), 3.68 (brs, 2H). The obtained data match those reported in literature.[6]

7a 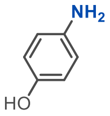

p-Aminophenol (**7a**). White solid, 1.77 mg (80%). ^{1}H-NMR (400 MHz, DMSO-d_6): δ = 8.31 (s, 1H), 6.49 – 6.43 (m, 2H), 6.43 – 6.37 (m, 2H), 4.36 (s, 2H). The obtained data match those reported in literature.[7]

8a 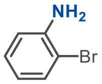

o-Bromoaniline (**8a**). Brown oil, 3.07 mg (89%). ^{1}H-NMR (400 MHz, $CDCl_3$): δ = 7.40 (dd, J = 8.0, 1.5 Hz, 1H), 7.15 – 7.06 (m, 1H), 6.77 (dd, J = 8.0, 1.5 Hz, 1H), 6.66 – 6.58 (m, 1H), 4.07 (brs, 2H). The obtained data match those reported in literature.[8]

9a 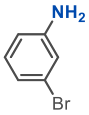

m-Bromoaniline (**9a**). Brown oil, 2.82 mg (82%). ^{1}H-NMR (400 MHz, $CDCl_3$): δ = 7.00 (t, J = 7.9 Hz, 1H), 6.91 – 6.76 (m, 2H), 6.64 – 6.52 (m, 1H), 3.69 (brs, 2H). The obtained data match those reported in literature.[9]

10a 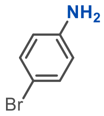

p-Bromoaniline (**10a**). Yellow solid, 2.79 mg (81%). ^{1}H-NMR (400 MHz, $CDCl_3$): δ = 7.33 – 7.17 (m, 2H), 6.59 – 6.53 (m, 2H), 3.66 (brs, 2H). The obtained data match those reported in literature.[4]

11a 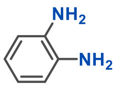

1,2-Diaminobenzene (**11a**). Beige solid, 2.15 mg (78%). ^{1}H-NMR (400 MHz, $CDCl_3$): δ = 6.75 – 6.68 (m, 4H), 3.22 (s, 4H). The obtained data match those reported in literature.[10]

12a 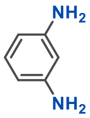

1,3-Diaminobenzene (**12a**). White solid, 2.82 mg (82%). ^{1}H-NMR (400 MHz, $CDCl_3$): δ = 6.94 (t, *J* = 7.9 Hz, 1H), 6.12 (dd, *J* = 7.9, 2.2 Hz, 2H), 6.04 (t, *J* = 2.2 Hz, 1H), 3.55 (brs, 4H). The obtained data match those reported in literature.[11]

13a 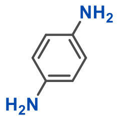

1,4-Diaminobenzene (**13a**). Beige solid, 2.26 mg (82%). ^{1}H-NMR (400 MHz, $CDCl_3$): δ = 6.57 (s, 4H), 3.35 (brs, 4H). The obtained data match those reported in literature.[12]

14a 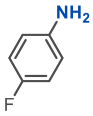

p-Fluoroaniline (**14a**). Pale-yellow oil, 1.93 mg (87%). ^{1}H-NMR (400 MHz, $CDCl_3$): δ = 6.91 – 6.81 (m, 2H), 6.65 – 6.58 (m, 2H), 3.53 (brs, 2H). The obtained data match those reported in literature.[4]

15a 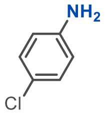

p-Chloroaniline (**15a**). White solid, 2.35 mg (92%). ^{1}H-NMR (400 MHz, $CDCl_3$): δ = 7.20 – 7.00 (m, 2H), 6.76 – 6.45 (m, 2H), 3.65 (brs, 2H). The obtained data match those reported in literature.[4]

16a 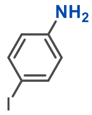

p-Iodoaniline (**16a**). Yellow solid, 3.76 mg (86%). ^{1}H-NMR (400 MHz, $CDCl_3$): δ = 7.41 (d, *J* = 8.6 Hz, 2H), 7.02 (d, *J* = 8.5 Hz, 2H, 4.53 (brs, 2H). The obtained data match those reported in literature.[13]

17a 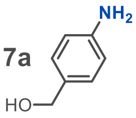

4-Aminobenzyl alcohol (**17a**). Beige solid, 2.03 mg (82%). ^{1}H-NMR (400 MHz, $CDCl_3$): δ = 7.20 – 7.13 (m, 2H), 6.71 – 6.64 (m, 2H), 4.56 (s, 2H), 3.68 (brs, 2H). The obtained data match those reported in literature.[14]

18a 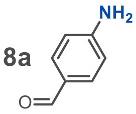

4-Aminobenzaldehyde (**18a**). 91%. The yield was determined by integration of ^{1}H-NMR spectrum signals relative to 4-nitrophenol as an internal standard. The obtained ^{1}H-NMR signals of **18a** matched those registered for commercial sample of 4-aminobenzaldehyde.^{1}H-NMR (400 MHz, 10% D_2O in PB, 50 mM, pH 8.0): δ = 9.45 (s, 1H), 7.70 – 7.59 (m, 2H), 6.78 – 6.71 (m, 2H).

19a 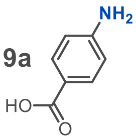

4-Aminobenzoic acid (**19a**). White solid, 2.51 mg (91%). ^{1}H-NMR (400 MHz, $CDCl_3$): δ = 7.91 (d, *J* = 6.5 Hz, 2H), 6.66 (d, *J* = 6.5 Hz, 2H), 4.13 (brs, 2H). The obtained data match those reported in literature.[15]

20a 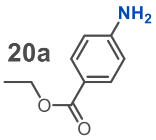

Ethyl 4-aminobenzoate (**20a**). White solid, 3.09 mg (93%). ^{1}H-NMR (400 MHz, $CDCl_3$): δ = 7.92 – 7.81 (m, 2H), 6.67 – 6.61 (m, 2H), 4.32 (q, *J* = 7.2 Hz, 2H), 4.07 (brs, 2H), 1.36 (t, *J* = 7.1 Hz, 3H). The obtained data match those reported in literature.[15]

21a 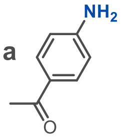

4-Aminoacetophenone (**21a**). White solid, 2.44 mg (90%). ^{1}H-NMR (400 MHz, $CDCl_3$): δ = 7.85 – 7.77 (m, 2H), 6.69 – 6.60 (m, 2H), 4.13 (brs, 2H), 2.51 (s, 3H). The obtained data match those reported in literature.[6]

22a 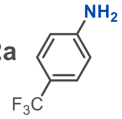

4-(Trifluoromethyl)aniline (**22a**). White solid, 2.73 mg (85%). ^{1}H-NMR (400 MHz, $CDCl_3$): δ = 7.39 (d, J = 8.3 Hz, 2H), 6.69 (d, J = 8.3 Hz, 2H), 3.93 (brs, 2H). The obtained data match those reported in literature.[16]

23a 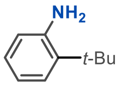

o-tert-Butylaniline (**23a**). White solid, 2.57 mg (86%). ^{1}H-NMR (400 MHz, $CDCl_3$): 7.25 (dd, J = 7.8, 1.6 Hz, 1H), 7.10 – 7.02 (m, 1H), 6.80 – 6.74 (m, 1H), 6.70 (dd, J = 7.8, 1.4 Hz, 1H), 4.13 (brs, 2H), 1.43 (s, 9H). The obtained data match those reported in literature.[17]

24a 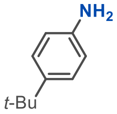

p-tert-Butylaniline (**24a**). Pale-yellow liquid, 2.66 mg (89%). ^{1}H-NMR (400 MHz, $CDCl_3$): δ = 7.19 (d, J = 8.6 Hz, 2H), 6.67 (d, J = 8.7 Hz, 2H), 3.63 (brs, 2H), 1.28 (s, 9H). The obtained data match those reported in literature.[18]

25a 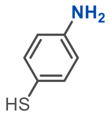

4-Aminothiophenol (**25a**). 78%. The yield was determined by integration of ^{1}H-NMR spectrum signals relative to 4-nitrophenol as an internal standard. The obtained ^{1}H-NMR signals of **25a** matched those registered for commercial sample of 4-aminothiophenol.^{1}H-NMR (400 MHz, 10% D_2O in PB, 50 mM, pH 6.0): δ = 7.19 – 7.10 (m, 2H), 6.75 – 6.63 (m, 2H).

26a 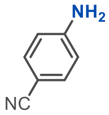

4-Aminobenzonitrile (**26a**). Pale-yellow solid, 1.96 mg (83%). ^{1}H-NMR (400 MHz, $CDCl_3$): δ = 7.48 – 7.37 (m, 2H), 6.69 – 6.58 (m, 2H), 4.12 (brs, 2H). The obtained data match those reported in literature.[11]

27a 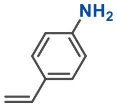

4-Aminostyrene (**27a**). Yellow oil, 1.91 mg (83%). ^{1}H-NMR (400 MHz, methanol-d_4): δ = 7.18 (d, J = 8.5 Hz, 2H), 6.67 (d, J = 8.5 Hz, 2H), 6.59 (dd, J = 17.6, 10.9 Hz, 1H), 5.51 (dd, J = 17.6, 1.2 Hz, 1H), 4.96 (dd, J = 10.9, 1.2 Hz, 1H). ^{1}H-NMR signals matched with the ^{1}H-NMR signals of commercial sample of 4-aminostyrene measured in methanol-d_4.

28a 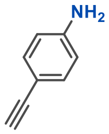

4-Ethynylaniline (**28a**). Yellow solid, 1.92 mg (82%). ^{1}H-NMR (400 MHz, DMSO-d_6): δ = 7.11 (d, J = 8.5 Hz, 2H), 6.53 – 6.45 (m, 2H), 5.49 (s, 2H), 3.76 (s, 1H). The obtained data match those reported in literature.[19]

29a 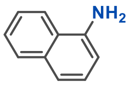

1-Naphtylamine (**29a**). White solid, 2.75 mg (96%). ^{1}H-NMR (400 MHz, $CDCl_3$): δ = 7.87 – 7.76 (m, 2H), 7.50 – 7.41 (m, 2H), 7.36 – 7.27 (m, 2H), 6.79 (dd, J = 6.8, 1.7 Hz, 1H), 4.15 (s, 2H). The obtained data match those reported in literature.[18]

30a

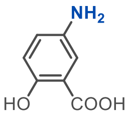

5-Aminosalicylic acid (**30a**). White solid, 2.69 mg (88%). ^{1}H-NMR (400 MHz, DMSO-d_6): δ = 7.11 (s, 1H), 6.84 (dd, J = 8.7, 2.9 Hz, 1H), 6.67 (d, J = 8.7 Hz, 1H). The obtained data match those reported in literature.[20]

VI. Preparative Gram-Scale Hydrogenation

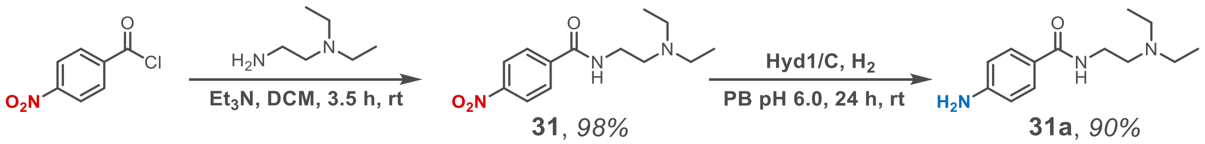

The protocol for the synthesis of **31** was adapted from Cong-shan Jiang *et al.*[21] Solution of 4-nitrobenzoyl chloride (5.57 g, 30 mmol, 1.00 eq) in DCM (40 mL) was added to the solution of *N*,*N*-diethylethylenediamine (5.62 mL, 40 mmol, 1.33 eq) and triethylamine (6.90 mL, 50 mmol, 1.67 eq) in DCM (60 mL) at 0 °C. The reaction mixture was then stirred at room temperature overnight. After that, DCM (60 mL) was added and the reaction mixture was washed with water (100 mL), 1M aqueous solution of NaOH (100 mL), brine (50 mL), dried with anhydrous Na_2SO_4, and filtered. The solvent was removed *in vacuo* to yield 7.80 g of **31** as a yellow solid (98%), which was directly used for further hydrogenation. The ^{1}H-NMR spectroscopy data match those reported in literature.[22] ^{1}H-NMR (400 MHz, $CDCl_3$): δ = 8.33 – 8.25 (m, 2H), 7.96 – 7.89 (m, 2H), 7.08 (s, 1H), 3.53 – 3.46 (m, 2H), 2.66 (dd, J = 6.4, 5.4 Hz, 2H), 2.57 (q, J = 7.1 Hz, 4H), 1.04 (t, J = 7.1 Hz, 6H).

Catalyst preparation was carried out in a glove box (Glove Box Technology Ltd.) under a protective N_2 atmosphere (O_2 < 3 ppm). A suspension of 264 mg of carbon black in 13.2 mL of PB (50 mM, pH 6.0) was sonicated in a 15 mL FALCON™ tube for 1 hour. After that, 1.02 mL of Hyd-1 solution (6.5 mg/mL) was added (C:Hyd-1 = 40:1 mass ratio), the mixture was gently mixed and left in the fridge (4 °C) for 1 hour. Next, the suspension of the catalyst was centrifuged (10 min, 11309 × g), the supernatant was decanted, and the catalyst was resuspended in 25 mL of PB (50 mM, pH 6.0). Resuspension-centrifugation-decanting steps were repeated 5 times, and then the catalyst was resuspended in 25 mL of PB (50 mM, pH 6.0). Then, 50 mL of stock solution of substrate **31** in MeCN (100 mM) was transferred to a three-neck round-bottom flask (1 L) equipped with a stirring bar, the catalyst suspension was added, and the volume was adjusted to a total volume of 500 mL with the same buffer. The flask was closed with three SUBA-SEAL® septa and removed from the glove box. The hydrogen line was connected, and the reaction was run at a 30 mL/min flow of H_2 at 25 °C in a temperature-controlled oil bath (Supplementary Fig. 36). Reaction completion was confirmed after 24 hours by ^{1}H-NMR spectroscopy (Supplementary Fig. 37). To prepare a sample for the ^{1}H-NMR analysis, an aliquot of 480 µL of the reaction mixture was centrifuged (3 min, 14100 × g), 450 µL of supernatant was placed in the NMR tube, and 50 µL of D_2O was added. ^{1}H-NMR spectrum was measured with water signal suppression. The reaction mixture was transferred into a centrifuge bottle and centrifuged for 20 min at 11309 × g. The supernatant was transferred into a separation funnel and washed with EtOAc (2×50 mL). The aqueous layer was collected into an Erlenmeyer flask and pH was adjusted to ~10-11 by slow addition of saturated aqueous K_2CO_3 solution followed by addition of brine (100 mL). The resulting solution was extracted with EtOAc (10×50 mL), combined organic layers were washed with brine (100 mL), dried with Na_2SO_4, filtered, and concentrated *in vacuo* to yield 1.10 g of **31a** as a yellow oil (90%, 96% purity). Analytical sample for ^{1}H-NMR analysis was obtained by flash column chromatography purification (silica gel, 2.5% MeOH in DCM + 1% Et_3N, R_f = 0.14). ^{1}H-NMR (400 MHz, $CDCl_3$): δ = 7.66 – 7.53 (m, 2H), 6.79 (s, 1H), 6.69 – 6.59 (m, 2H), 3.98 (s, 2H), 3.52 – 3.38 (m, 2H), 2.63 (t, J = 6.0 Hz, 2H), 2.56 (q, J = 7.1 Hz, 4H), 1.03 (t, J = 7.1 Hz, 6H). The obtained data match those reported in literature.[23]

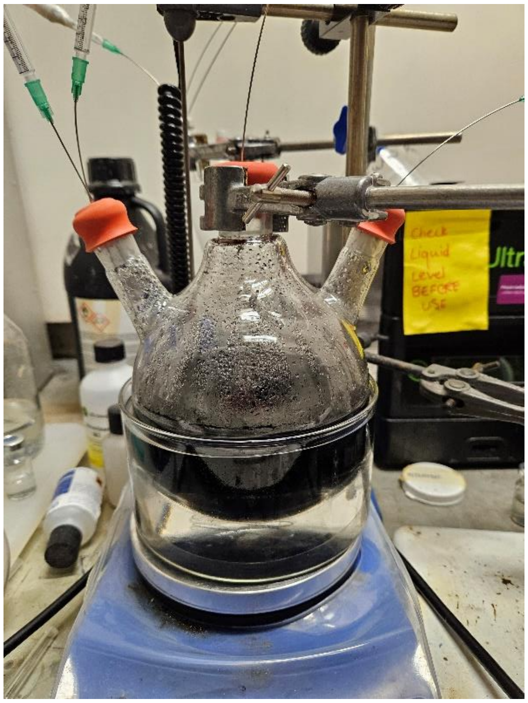

Supplementary Figure 36. Reaction set-up for running gram-scale hydrogenation reaction.

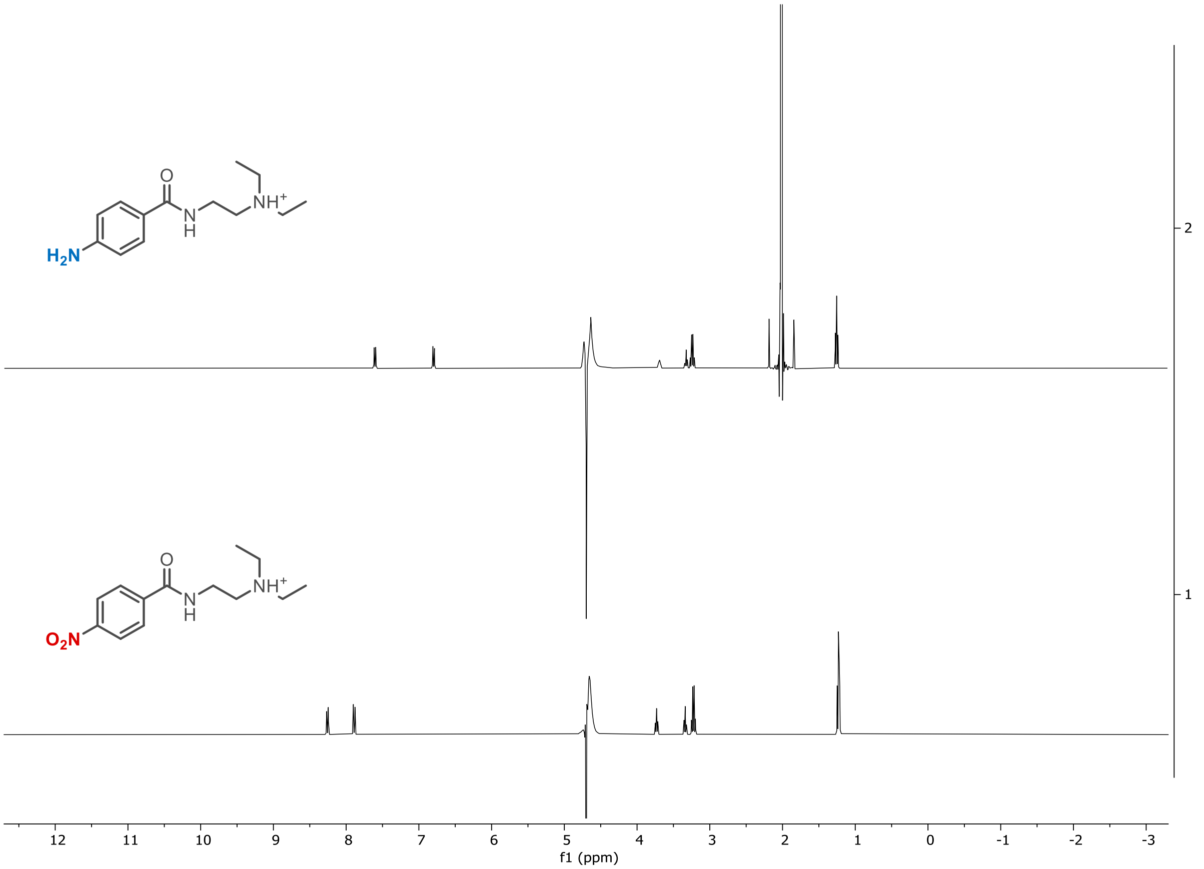

Supplementary Figure 37. ^{1}H-NMR spectra (400 MHz, 298 K, 10% D_2O in PB, 50 mM, pH 6.0) of substrate **31** and reaction mixture after 24 hours of reaction time.

VII. Exploration of the Catalyst and its Operating Conditions

Screening of various reaction conditions in presence of the catalyst was performed according to the procedures described in sections II and IV by varying one of the parameters at the time. Nitrobenzene (**1**) was chosen as the model substrates for these experiments. Conversions and ratios of products were determined by integration of ^{1}H-NMR spectra.

1. Influence of the catalyst loading

Variation of the catalyst loading as shown in Supplementary Table 3 using nitrobenzene (**1**) as a substrate did not indicate any difference between reactions using 2·**X**, **X**, and 0.5·**X** catalyst loadings yielding aniline (**1a**) as a single product after 24 hours of reaction time suggesting that the catalyst loading can be decreased to 0.5·**X** for substrate **1** (Supplementary Table 3, Supplementary Fig. 38). Screening of other substrates indicated the need to use higher catalyst loading than 0.5·**X**; standard catalyst loading **X** gave full conversion of nitro substrate to corresponding amine for most of substrates from Fig. 3 of the Manuscript; some substrates required higher catalyst loading (see Fig. 3 of the Manuscript and SI Section IV). Further decrease of the catalyst loading to 0.25·**X** and 0.125·**X** gave full conversion of nitrobenzene (**1**) with *N*-phenylhydroxylamine as the major product (Supplementary Fig. 38).

Supplementary Table 3. Results of variation of catalyst loading for hydrogenation of nitrobenzene (**1**). Conversions and ratios of products were determined by integration of ^{1}H-NMR spectra.

Entry	Cat. Loading[1]	Conversion of 1 (%)	1b:1a
1	2·**X**	100	0:100
2	**X**	100	0:100
3	0.5·**X**	100	0:100
4	0.25·**X**	100	84:16
5	0.125·**X**	100	93:7

[1]***X** is a standard catalyst loading of 1.32 mg of Hyd-1 per 1 mmol of substrate, C:Hyd-1 = 40:1 mass ratio.*

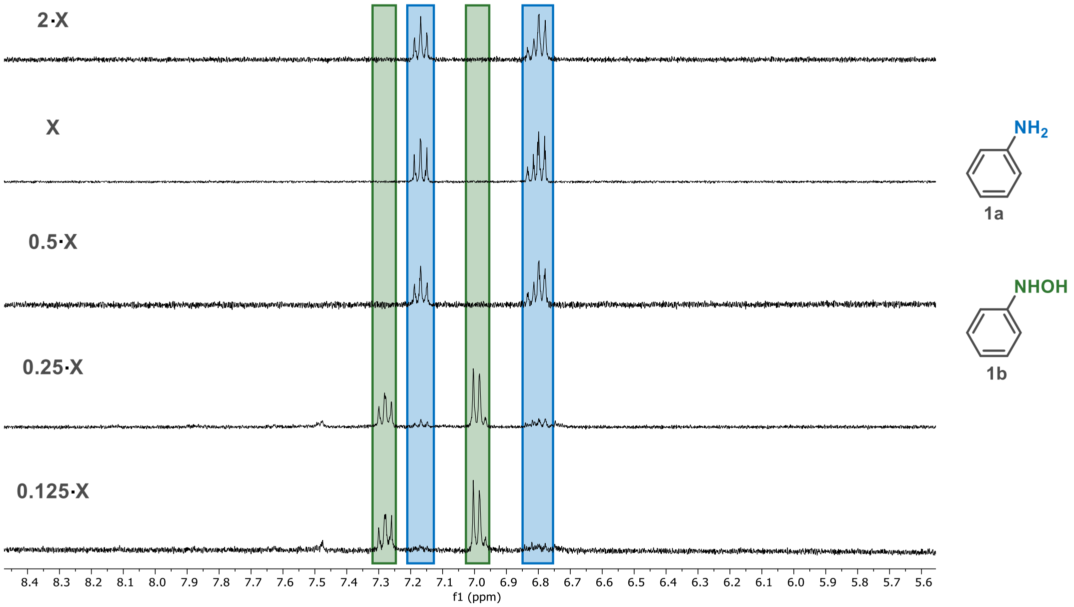

Supplementary Figure 38. ^{1}H-NMR spectra (400 MHz, 298 K, 10% D_2O in PB, 50 mM, pH 6.0) of nitrobenzene (**1**) hydrogenation reaction mixtures after 24 hours under standard reaction conditions (see Section IV) with variation of catalyst loading (indicated on the left side of each spectrum, **X** is a standard catalyst loading of 1.32 mg of Hyd-1 per 1 mmol of substrate, C:Hyd-1 = 40:1 mass ratio). Full conversion to aniline (**1a**, blue markers) was achieved when using 2·**X**, **X**, and 0.5·**X** catalyst loadings; *N*-phenylhydroxylamine (**1b**) labeled with green markers).

2. Influence of MeCN as a co-solvent

To test the system tolerance for organic co-solvents, different amounts of MeCN (0–50%) were tested under standard reaction conditions (Supplementary Table 4, Supplementary Fig. 39). Obtained results indicate that the catalytic system tolerates up to 20% of MeCN yielding aniline (**1a**) as a single product. With 30% of MeCN as a co-solvent full conversion of starting material was observed after 24 hours of reaction time yielding *N*-phenylhydroxylamine (**1b**) and **1a** in 15:85 ratio. Addition of 40% of MeCN as a co-solvent slowed down the conversion of **1** to 82% yielding **1b** and **1a** in 89:11 ratio. After 24 hours of reaction time, reactions with 50% of MeCN as a co-solvent reached 26% conversion of starting material to **1b** as a single product.

Supplementary Table 4. Results of variation of MeCN additive (% v/v) as a co-solvent. Conversions and ratios of products were determined by integration of ^{1}H-NMR spectra.

Entry	MeCN (%)	Conversion of 1(%)	1b:1a
1	0	100	0:100
2	10	100	0:100
3	20	100	0:100
4	30	100	15:85
5	40	82	89:11
6	50	26	100:0

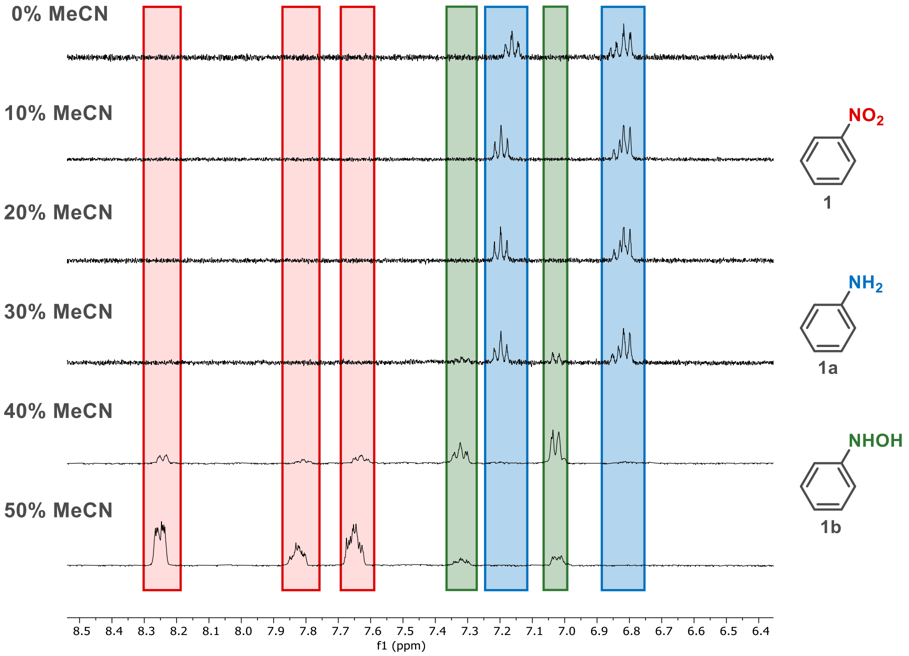

Supplementary Figure 39. ^{1}H-NMR spectra (400 MHz, 298 K, 10% D_2O in PB, 50 mM, pH 6.0) of nitrobenzene (**1**, red markers) hydrogenation reaction mixtures after 24 hours under standard reaction conditions (see Section IV) with different additives of MeCN as a co-solvent (indicated as % on the left side of each spectrum). Full conversion to **1a** (blue markers) observed in presence of 0–20% MeCN; full conversion to the mixture of **1a** and **1b** (green markers) observed in presence of 30% MeCN; incomplete conversion to **1b** observed in presence of 40% and 50% of MeCN.

3. Influence of pH

PB (50 mM) was prepared with pH values adjusted to 5, 6, 7, 8, and 9. The catalytic system tolerates pH range 5–9 without any drop of conversion of **1** yielding **1a** as a single product after 24 hours of reaction (Supplementary Fig. 40).

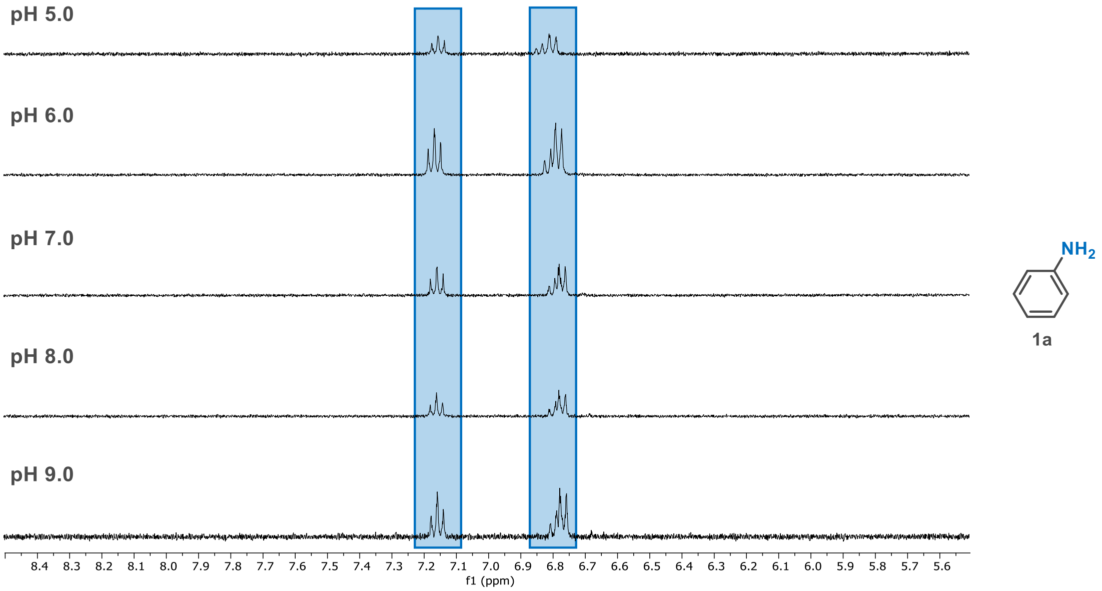

Supplementary Figure 40. ^{1}H-NMR spectra (400 MHz, 298 K, 10% D_2O in PB, 50 mM) of nitrobenzene (**1**) hydrogenation reaction mixtures after 24 hours under standard reaction conditions (see Section IV) at different pH (indicated on the left side of each spectrum, 50 mM). Full conversion to aniline (**1a**, blue markers) was observed in each case.

4. Influence of buffer concentration

Reduction of nitrobenzene (**1**) was performed in 10, 20, 50, and 100 mM PB (pH 6.0) resulting in full conversion to aniline (**1a**) after 24 hours of reaction time (Supplementary Fig. 41).

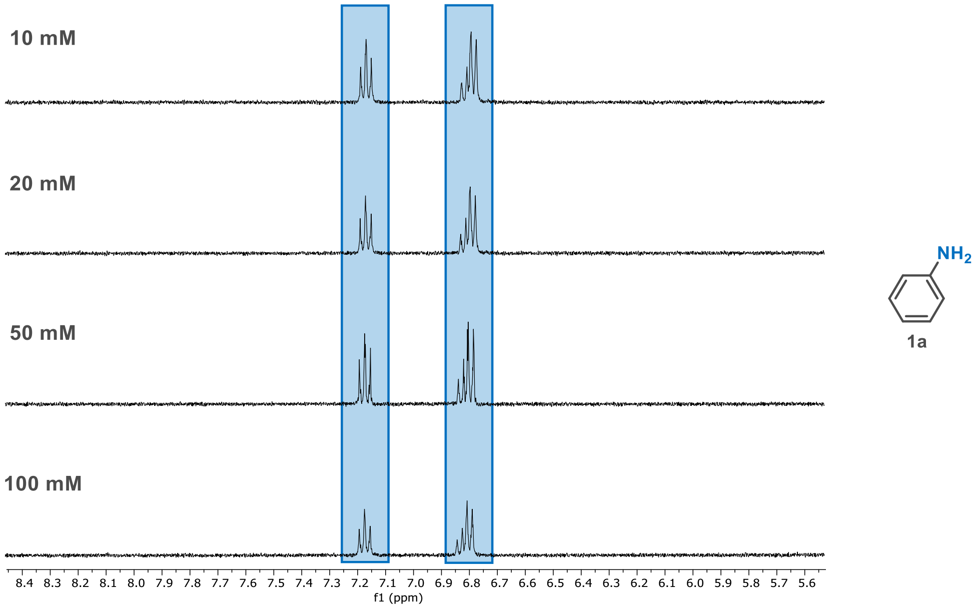

Supplementary Figure 41. ^{1}H-NMR spectra (400 MHz, 298 K, 10% D_2O PB, pH 6.0) of nitrobenzene (**1**) hydrogenation reaction mixtures after 24 hours under standard reaction conditions (see Section IV) at different PB concentrations (indicated on the left side of each spectrum, pH 6.0). Full conversion to aniline (**1a**, blue markers) was observed in each case.

5. Influence of substrate concentration

Reduction of nitrobenzene (**1**) was performed at different concentrations of substrate: 10, 20, 50, and 100 mM of **1** (Supplementary Table 5, Fig. 42). Reactions with 10 mM concentration of **1** were completed after 24 hours of reaction time yielding aniline (**1a**) as a single product. Increase of concentration of **1** to 20 and 50 mM did not impact the conversion of starting material but gave mixture of *N*-phenylhydroxylamine (**1b**) and **1a** (4:96 for 20 mM of **1** and 18:82 for 50 mM of **1**). Reactions with 100 mM concentration of **1** gave 42% conversion after 24 hours of reaction time yielding 13:87 mixture of **1b** to **1a**.

Supplementary Table 5. Results of variation of substrate **1** initial concentration. Conversions and ratios of products were determined by integration of ^{1}H-NMR spectra.

Entry	Concentration of 1 (mM)	Conversion of 1 (%)	1b:1a
1	10	100	0:100
2	20	100	4:96
3	50	100	18:82
4	100	42	13:87

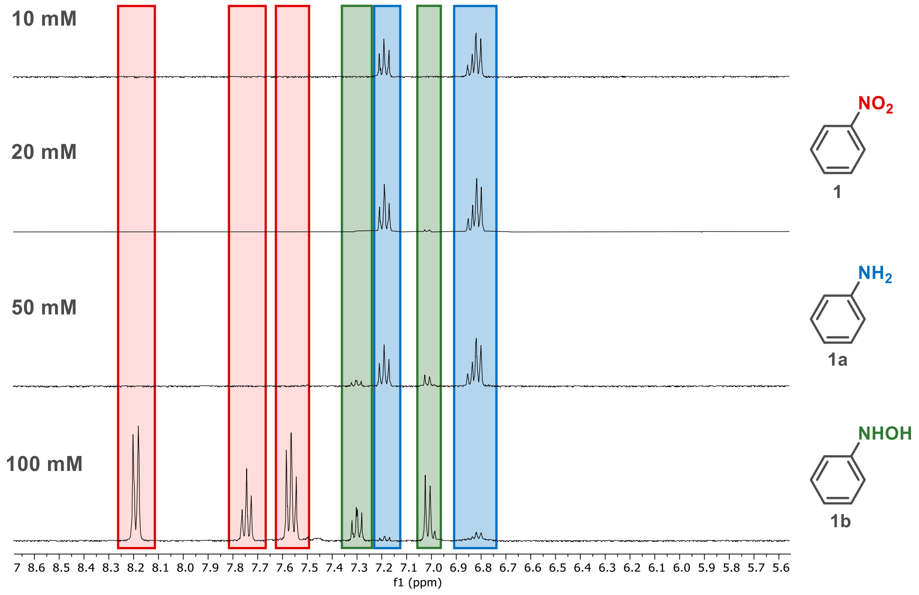

Supplementary Figure 42. ^{1}H-NMR spectra (400 MHz, 298 K, 10% D_2O in PB, 50mM. pH 6.0) of nitrobenzene (**1**, red markers) hydrogenation reaction mixtures after 24 hours under standard reaction conditions (see Section IV) at different substrate concentrations (indicated on the left side of each spectrum). Full conversion to aniline (**1a**, blue markers) observed at 10 mM concentration of **1**; full conversion to the mixture of **1a** and **1b** (green markers) observed at 20 mM and 50 mM concentration of **1**; incomplete conversion to a mixture of **1b** and aniline **1a** observed at 100 mM initial concentration of **1**.

VIII. Catalyst Recycling

Reaction set-up was carried out in a glove box (Glove Box Technology Ltd.) under a protective N_2 atmosphere (O_2 < 3 ppm). Reactions were run on a 2 mL scale with a 10 mM concentration of **1** in PB (50 mM, pH 6.0) at room temperature under a gentle H_2 flow in an ASYNT OCTO MINI REACTOR. A stock solution of substrate in buffer was transferred to a reaction vessel, 100 µL of catalyst suspension in PB (50 mM, pH 6.0) was added, and the volume was adjusted to a total of 2 mL with the corresponding buffer. The reactor was closed and removed from the glove box. The hydrogen line was connected, and the reaction was run at a 30 mL/min flow of H_2. After 24 hours of reaction time, the reaction mixture was transferred to an EPPENDORF TUBE® and centrifuged (3 min, 14100 × g), the supernatant was removed and analysed by ^{1}H-NMR spectroscopy. The remaining catalyst was resuspended in 100 µL PB (50 mM, pH 6.0) and subjected to the next reaction cycle. The same procedure was repeated until a drop of conversion of the starting material was observed (Supplementary Fig. 43). The first 5 cycles of the catalyst reuse gave full conversion of **1** to aniline (**1a**). Starting from cycle 6 until cycle 13 of the catalyst reuse there was no drop of conversion of **1** observed, however more *N*-phenylhydroxylamine (**1b**) was formed with each cycle. After cycles 14 and 15 of the catalyst reuse a drop of conversion (~80%) of **1** was observed and the experiment was stopped.

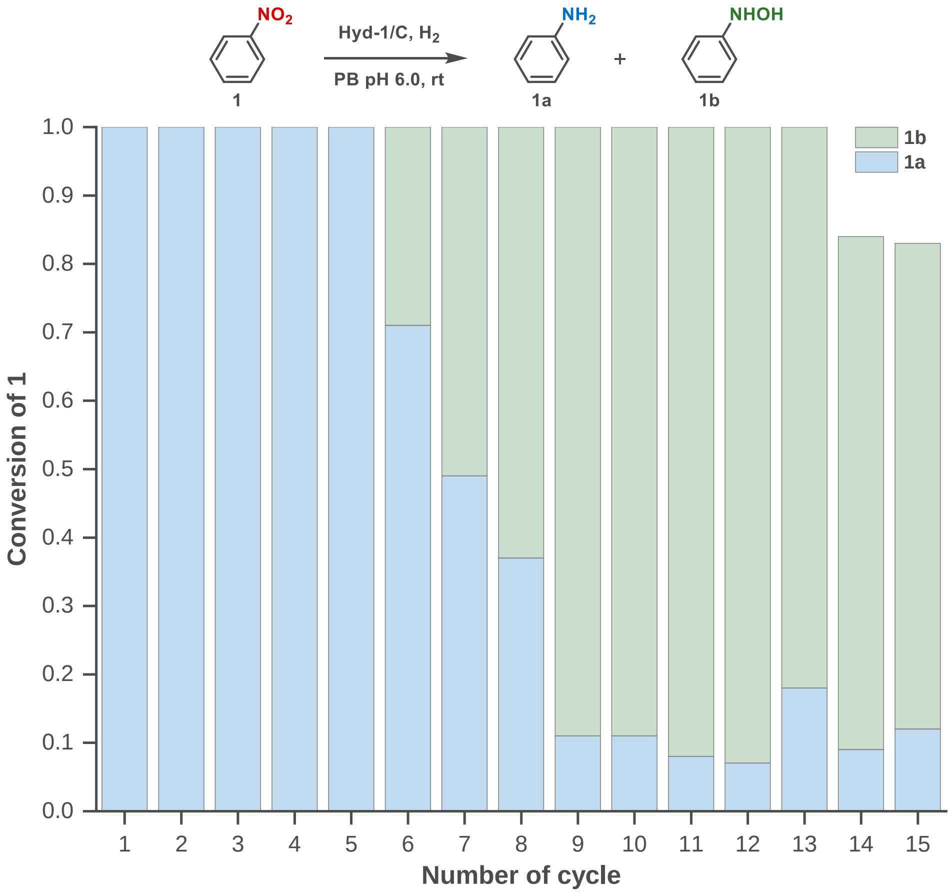

Supplementary Figure 43. Recycling of Hyd-1/C catalyst in the hydrogenation of nitrobenzene (**1**) at 10 mM concentration of **1** in 2 mL of PB (50 mM, pH 6.0).

IX. Electrochemical Procedures

All electrochemistry was conducted using a 3-electrode cell consisting of a pyrolytic graphite edge (PGE) working electrode (WE, 0.031 cm^2), a saturated calomel reference electrode (SCE, PALMSENS BV), a coiled platinum wire counter electrode (CE), and aqueous electrolyte (PB 50 mM, pH 6.0 unless otherwise specified). Cell temperature was kept constant at 25 ^oC by a water jacket. All experiments were conducted in a N_2 atmosphere (O_2 < 3 ppm) glovebox (GLOVE BOX TECHNOLOGY LTD.).

1. Voltammetry of Hyd-1 and nitrobenzene

Cyclic voltammograms at stationary electrodes of nitrobenzene (**1**) (Fig. 2A of the Manuscript), hexylamine and nitrosobenzene (**1c**) were recorded using a PalmSens4 potentiostat (PALMSENS BV) and the associated PSTrace 5.8 software. Cyclic voltammograms (CVs) were recorded with n = 2 replicates on independent electrodes and 3 sequential CVs recorded within each replicate. Each measurement included a 20 s poise to equilibrate the electrode before the scan. Aqueous electrolyte for all measurements was PB (50 mM, pH 6.0) containing 1 mM substrate (note: where solubility is < 1 mM, this is a saturated solution). Each repeat included a control measurement in PB (50mM, pH 6.0) without substrate to ensure a clean electrode surface before the start of each measurement. Before use, each WE was polished through 600, 1200 and 4000 grit sandpapers to achieve a high shine, with three 5-second pulses in a sonicator in MilliQ-water and rinsing with ethanol between each grade of sandpaper.

Cyclic voltammograms (Supplementary Fig. 44 and 45), show the reversible 2 electron nitrosobenzene / *N*-phenylhydroxylamine couple under experimental conditions, confirming the correct assignment of the redox couple as identified in Fig. 2A of the Manuscript.

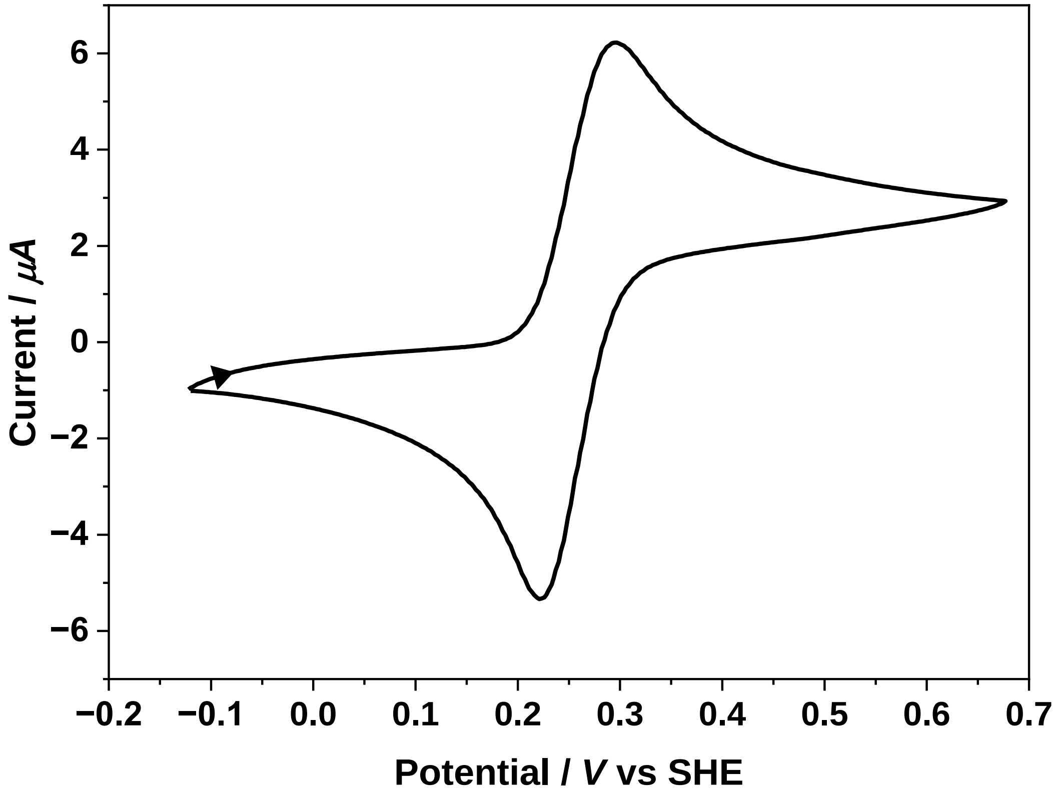

Supplementary Figure 44. Cyclic voltammogram of *N*-phenylhydroxylamine (**1b**) (10 mV/s, stationary electrode, PB 50 mM, pH 6.0, 1 mM *N*-phenylhydroxylamine). First cycle shown, arrow indicates direction of scan.

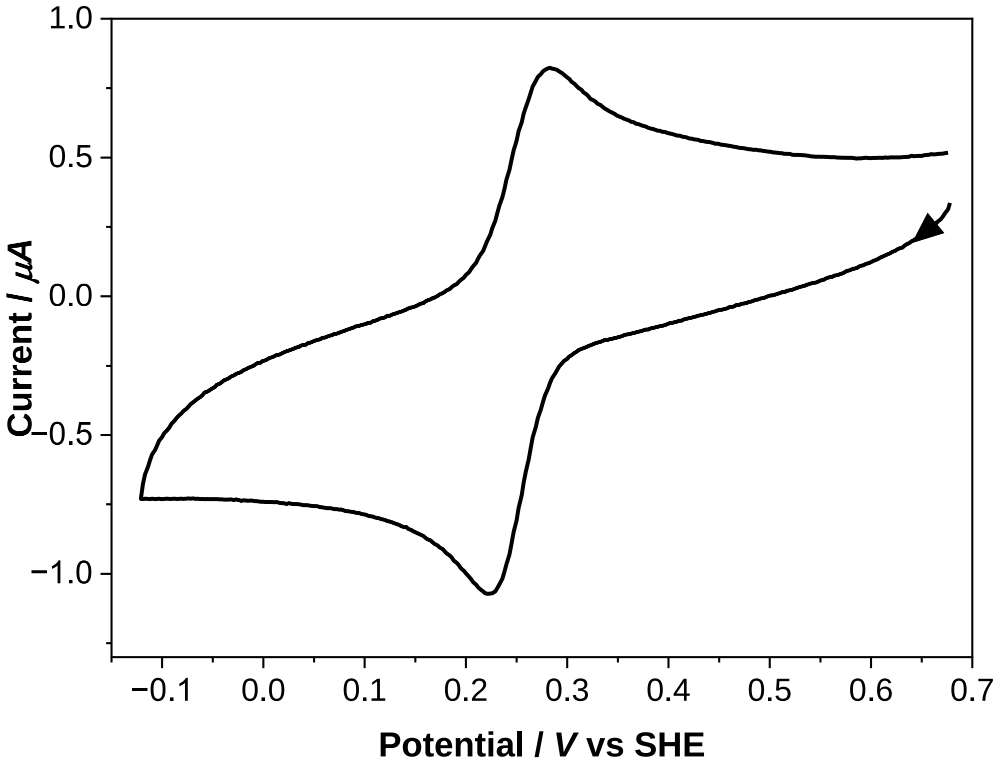

Supplementary Figure 45. Cyclic voltammogram of a saturated solution of nitrosobenzene (**1c**) (10 mV/s, stationary electrode, PB 50 mM, pH 6.0). The lower current, by comparison to the *N*-phenylhydroxylamine (**1b**), can be attributed to the lower solubility of **1c** in buffer. First cycle. Arrow indicates direction of scan.

2. Enzyme film voltammograms

To determine the onset potential of H_2 oxidation by Hyd-1 and Hyd-2 (Supplementary Fig. 46) voltammograms were recorded using an AUTOLAB PGSTAT30 potentiostat (ECOCHEMIE) and the associated Nova 1.1 software. The cell headspace was purged with a constant flow of 1000 scc/min H_2 gas before and during measurements. Aqueous electrolyte for all measurements was PB (50 mM, pH 6.0). Voltammograms were recorded with n = 2 replicates on independent electrodes. Each experiment included a control measurement with no enzyme film. Before use, each WE was polished with 400 grit sandpaper and rinsed with MilliQ-water, and was then modified with an enzyme film by dropping 2 μL of enzyme solution (Hyd-1 = 1.71 mg/mL, Hyd-2 = 6.3 mg/mL) onto the electrode which was allowed to incubate for 5-10 min before rinsing again with MilliQ water. The modified electrode was mounted onto a rotator (METROHM) and rotated at 3000 rpm for the duration of the measurements to negate mass transport effects.

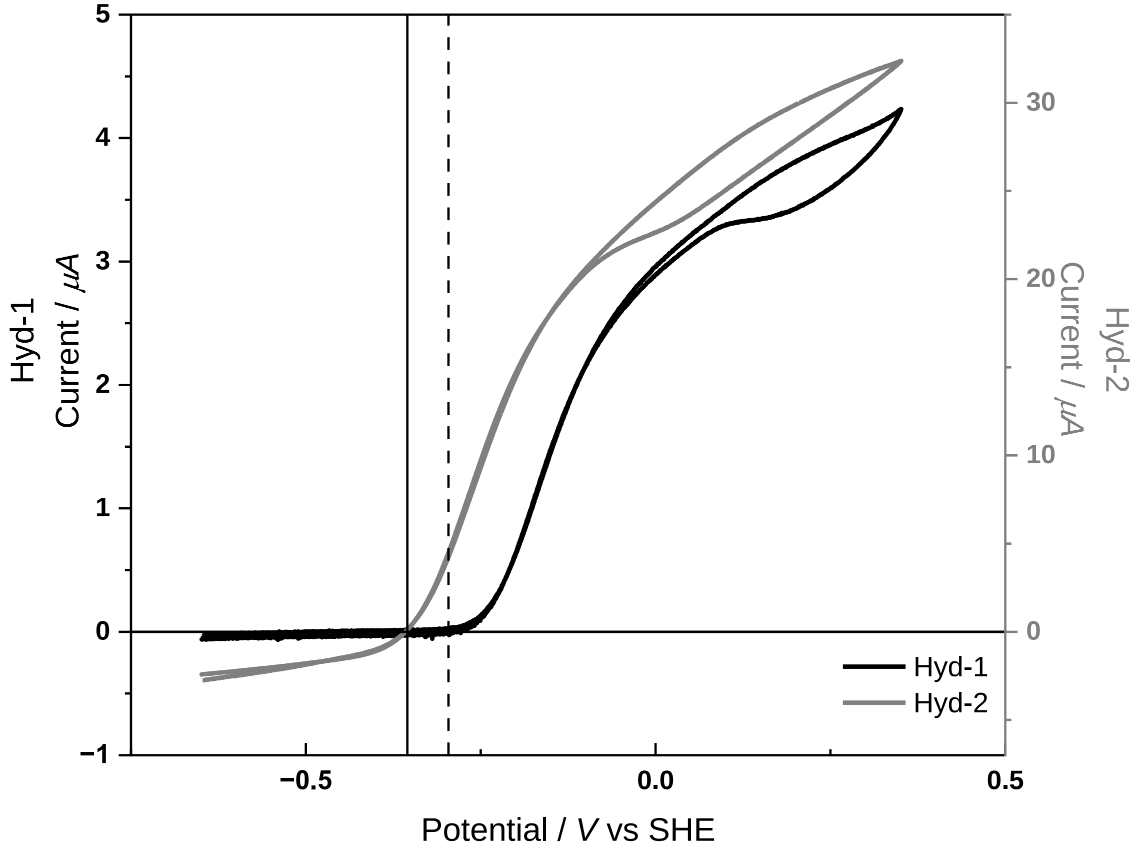

Supplementary Figure 46. Enzyme film cyclic voltammetry (1 mV/s, 3000 rpm, 50 mM PB pH 6.0, 1000 scc/min H_2 gas flow) for Hyd-1 (black) and Hyd-2 (gray). $E'(2H^+/H_2)$ couple (25 °C, 1 bar, pH 6.0) is indicated by a solid black line. The Hyd-2 onset was determined to be -0.355 V from two independent repeats. The onset for H_2 oxidation by Hyd-1 is determined here to be -0.296 V from two independent repeats (blue dashed line), representing an overpotential of 59 mV when compared to $E'(2H^+/H_2)$. The inflexion in the current on the return sweep (towards negative potentials) for each enzyme is well-understood to arise from reductive re-activation after a slow oxidative inactivation which occurs at high potential.[24]

Whole catalyst voltammograms were recorded by the same method as the enzyme film voltammograms with the exception that 3 µL of freshly prepared catalyst suspension (Hyd-1/C or Hyd-2/C) was drop-cast on to the polished electrode in place of the enzyme. These voltammograms show the resultant electrochemistry of the component catalyst with **1** under reaction conditions. The potential of zero crossing (the potential at which reduction and oxidation currents are of equal magnitude), E_{ZC}, is shown by a dashed line, and falls between the onset for H_2 oxidation by Hyd-1 and the onset for nitrobenzene reduction on carbon and gives an indication of the initial working potential of the catalyst. The effective electrochemical potential at the catalyst in contact with H*2* and a nitro-containing substrate can be assessed from the potential of zero crossing (E_{ZC}, the potential at which reduction and oxidation currents are of equal magnitude) in a linear sweep voltammogram with the catalyst immobilised on an electrode (Supplementary Fig. 47).[25] For Hyd-1/C in contact with H_2 and nitrobenzene, E_{ZC} lies at -0.251 V, between the onset for nitro reduction at carbon, and the H_2 oxidation onset for Hyd-1/C.

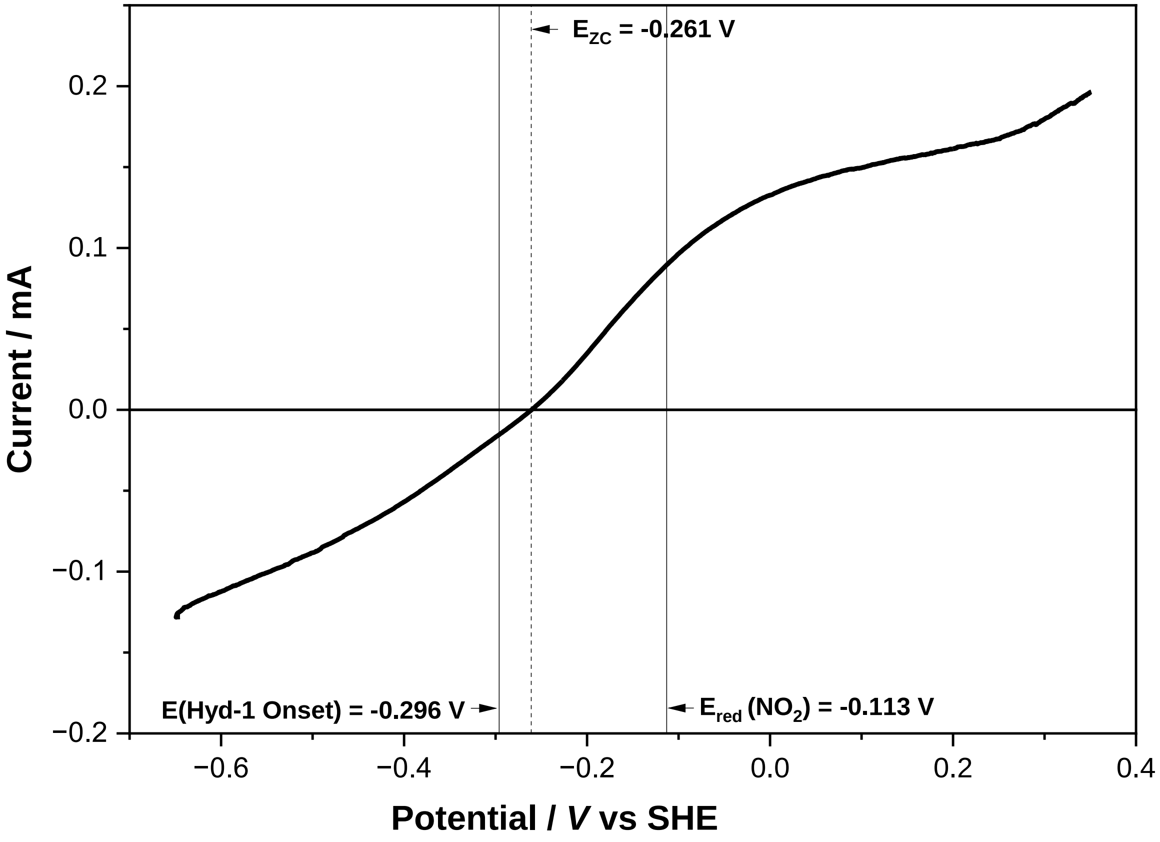

Supplementary Figure 47. Linear sweep voltammogram of Hyd-1/C catalyst (1 mV/s, 3000 rpm, 50 mM PB pH 6.0, 10 mM nitrobenzene, 1000 scc/min H_2 gas flow, i.e. in the presence of both H_2 and nitrobenzene).

3. Determination of substrate reduction onset potentials

A broader study of the reduction onset potentials in aqueous electrolyte of nitro-containing compounds was conducted to assess whether these thermodynamic predictions of catalyst reactivity were accurate. Since these are not currently available in literature under aqueous conditions, we used voltammetry of the substrates under reaction conditions to determine these.

Substrate reduction voltammograms (Supplementary Fig. 49–74) were recorded using a PalmSens4 (PALMSENS BV) potentiostat and the associated PSTrace 5.8 software. Cyclic voltammograms (CVs) were recorded with $n = 2$ replicates on independent electrodes and 3 sequential CVs recorded within each replicate following a 20 s poise to equilibrate the electrode. The first sweep of each (equivalent to a linear sweep voltammogram) was taken for analysis. Electrolyte for all measurements was PB (50 mM, pH 6.0) containing 1 mM substrate (note: where solubility is < 1 mM, this is a saturated solution). Each repeat included a control measurement in PB (50 mM, pH 6.0) without substrate. Before use, each WE was polished through 600, 1200 and 4000 grit sandpapers to achieve high shine, with three 5 second pulses in MilliQ-water in a sonicator and rinsing with ethanol between each grade of sandpaper. Working electrodes were then mounted onto an AutoLab (METROHM) rotator and were rotated at 3000 rpm, unless otherwise specified, during data acquisition to negate mass transport effects, therefore providing a study of substrate behavior at the carbon surface.

Onset potentials were then calculated by analysis of the first voltammogram for each substrate once the electrode was placed into solution. Checking for consistency with the control voltammogram taken before each measurement, a linear baseline correction was extrapolated from a region of no activity. The potential at which the difference between the measured current and the baseline began to increase exponentially, exceeding a threshold value of 10 nA (instrument resolution ~0.5 nA), was taken to be the onset potential. Each voltammogram and calculation was repeated with two independent electrodes to ensure true repeats on newly prepared carbon surface and both data points are reported in Supplementary Fig. 48.

Supplementary Table 6. Onset potentials as calculated from linear sweep voltammograms for the reduction of nitro compounds in aqueous conditions (PB, 50 mM, pH 6.0).

Entry	Substrate[1]	Nitro reduction onset potential / V vs SHE[2]
1	**1**	-0.113
2	**2**	-0.098
3	**3**	-0.046
4	**4**	-0.159
5	**5**	-0.063
6	**6**	-0.167
7	**7**	-0.198
8	**10**	-0.048
9	**13**	0.114[3]
10	**14**	-0.127
11	**15**	-0.128
12	**16**	-0.106
13	**17**	-0.019
14	**18**	0.026
15	**19**	0.030
16	**20**	-0.029
17	**21**	0.000
18	**22**	-0.093
19	**24**	-0.085
20	**25**	-0.130
21	**26**	0.005
22	**27**	-0.028
23	**28**	-0.037
24	**30**	-0.097
25	**32**	-0.313
	Hyd-1 onset H_2 oxidation (pH 6.0)	-0.267
	$E'(2H^+/H_2)$ (pH 6.0)	-0.355

[1]*See Figure 3 in Manuscript*
[2]*Mean calculated from 2 independent electrodes, n = 2. See Supplementary Fig. 48.*
[3]*First reduction potential reported.*

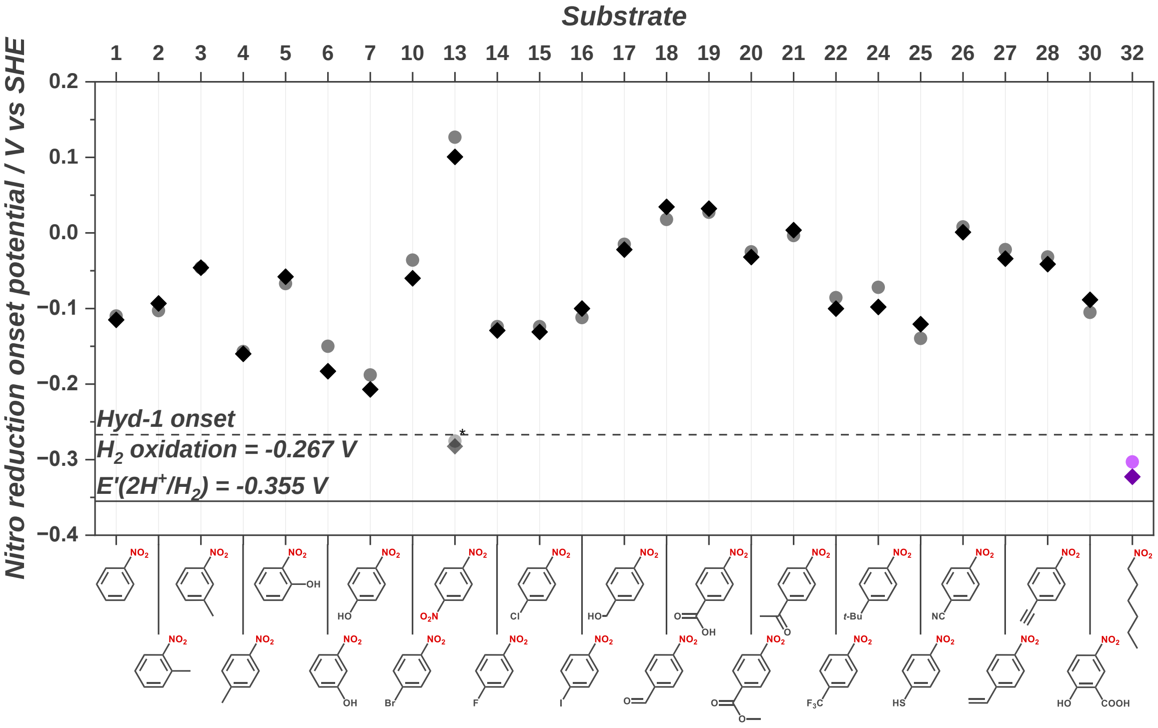

*The second NO_2 reduction is within error of the measurement where a true baseline cannot be established between the two reduction events and is accessible by Hyd-1/C.

Supplementary Figure 48. Comparison of the onset potential for the reduction of nitro compounds as determined by cyclic voltammetry across two independent repeats at different electrodes (pyrolytic graphic edge, rotated at 3000 rpm, scan rate, 10 mV/s; PB, 50 mM, pH 6.0; 1 mM substrate). Shown with a dotted line is the onset for Hyd-1 oxidation of H_2, at potentials more positive of this $2H^+$ and $2e^-$ are generated creating a negative potential at the carbon surface where reduction may occur. Shown with a solid line, is $E'(2H^+/H_2)$ (25 °C, 1 bar H_2, pH 6.0), which also corresponds with the Hyd-2 oxidation onset potential.

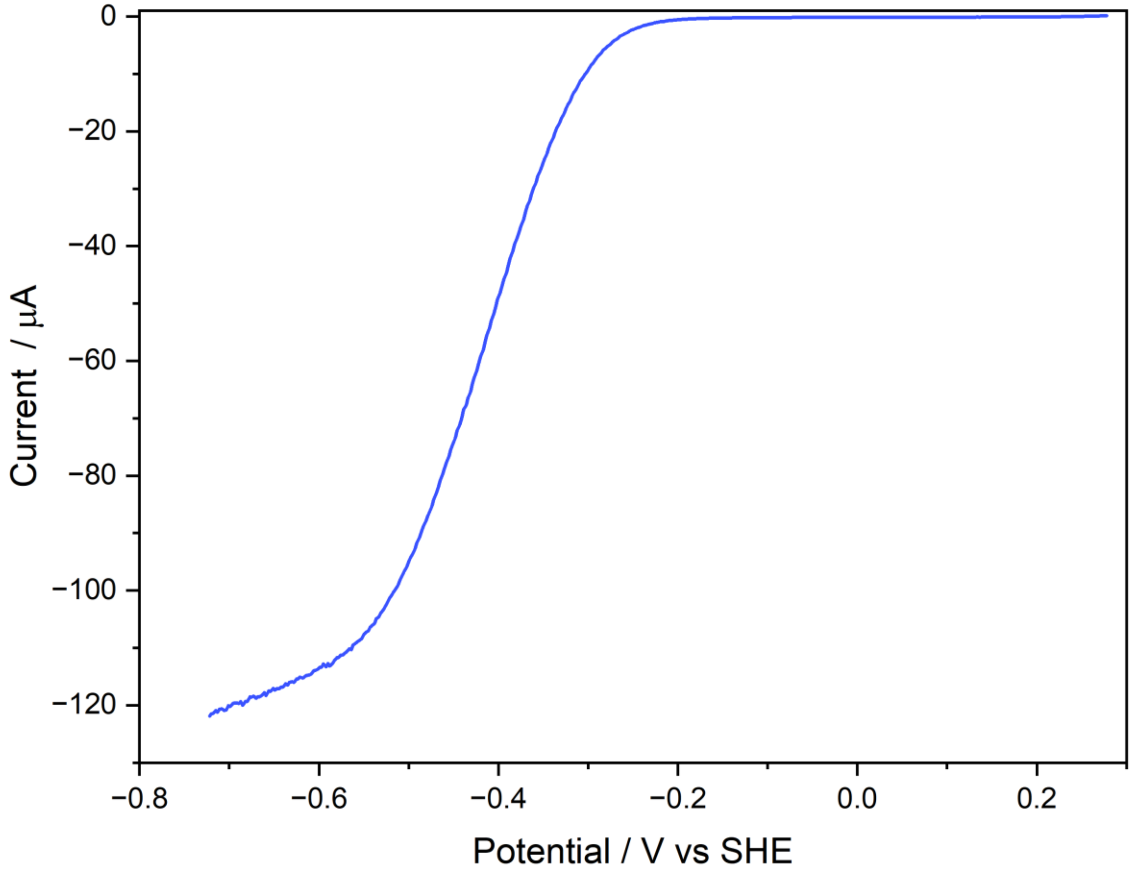

Supplementary Figure 49. Linear sweep voltammogram (0.01 V/s, 0.0024 V step) of substrate **1** in aqueous PB (50mM, pH 6.0) at 3000 rpm.

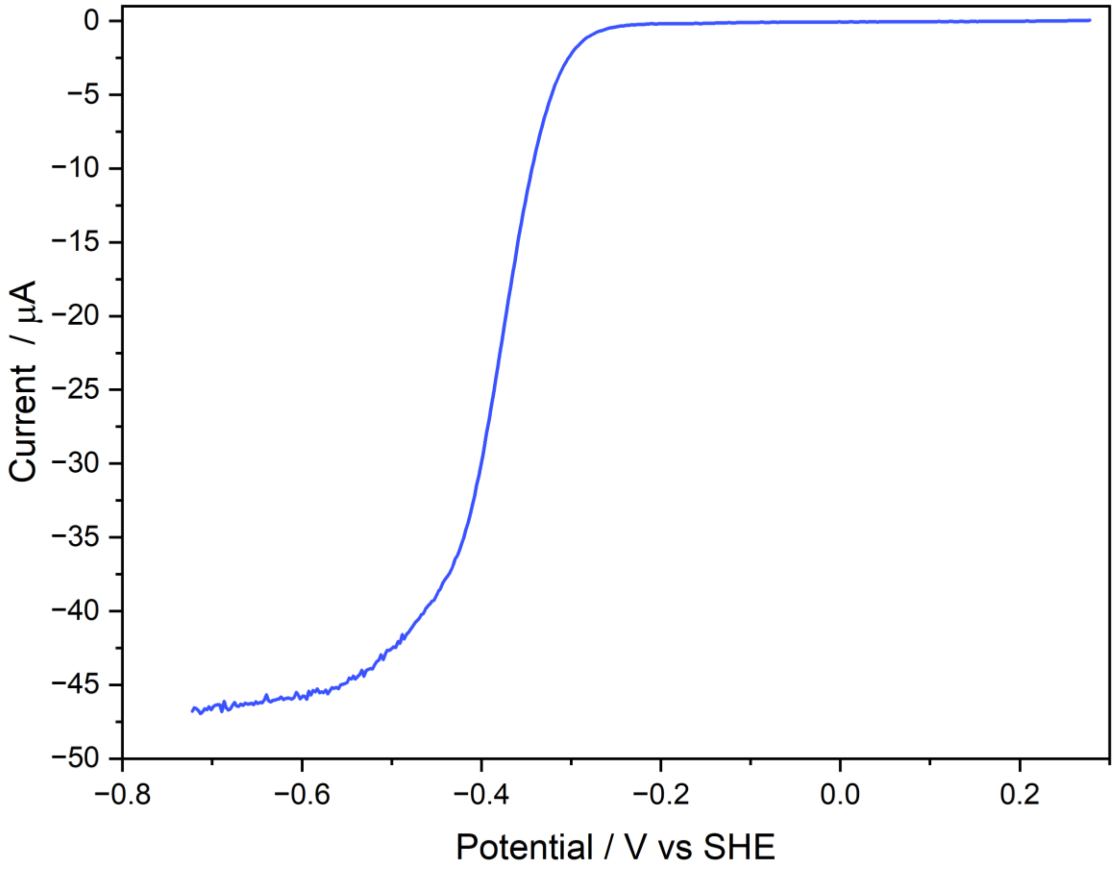

Supplementary Figure 50. Linear sweep voltammogram (0.01 V/s, 0.0024 V step) of substrate **2** in aqueous PB (50mM, pH 6.0) at 3000 rpm.

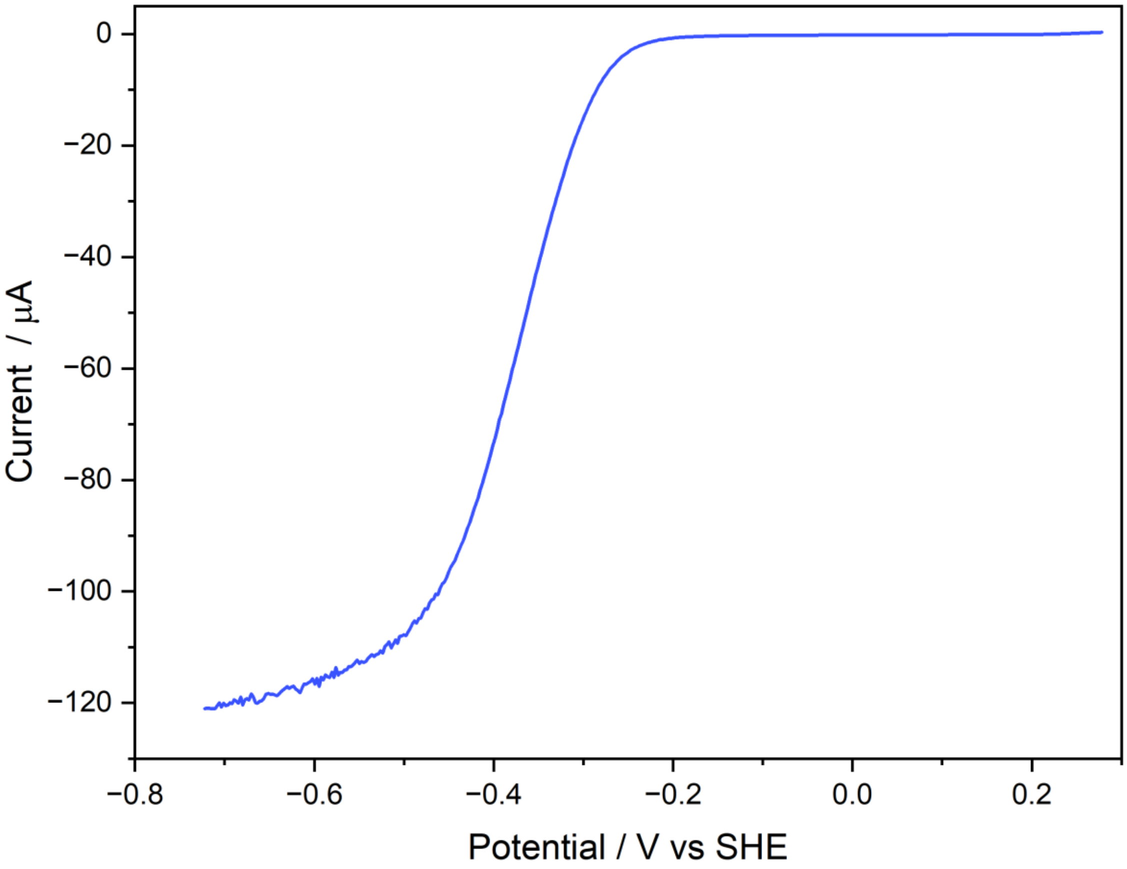

Supplementary Figure 51. Linear sweep voltammogram (0.01 V/s, 0.0024 V step) of substrate **3** in aqueous PB (50mM, pH 6.0) at 3000 rpm.

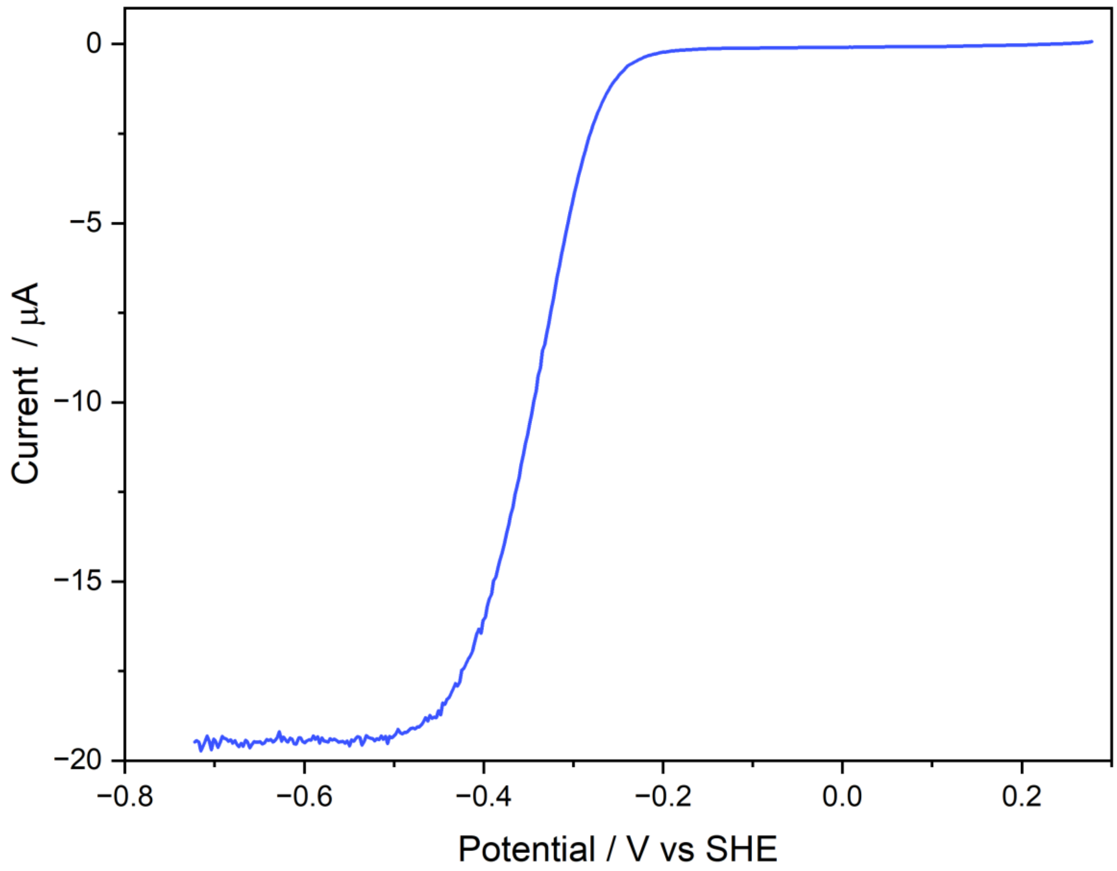

Supplementary Figure 52. Linear sweep voltammogram (0.01 V/s, 0.0024 V step) of substrate **4** in aqueous PB (50mM, pH 6.0) at 3000 rpm.

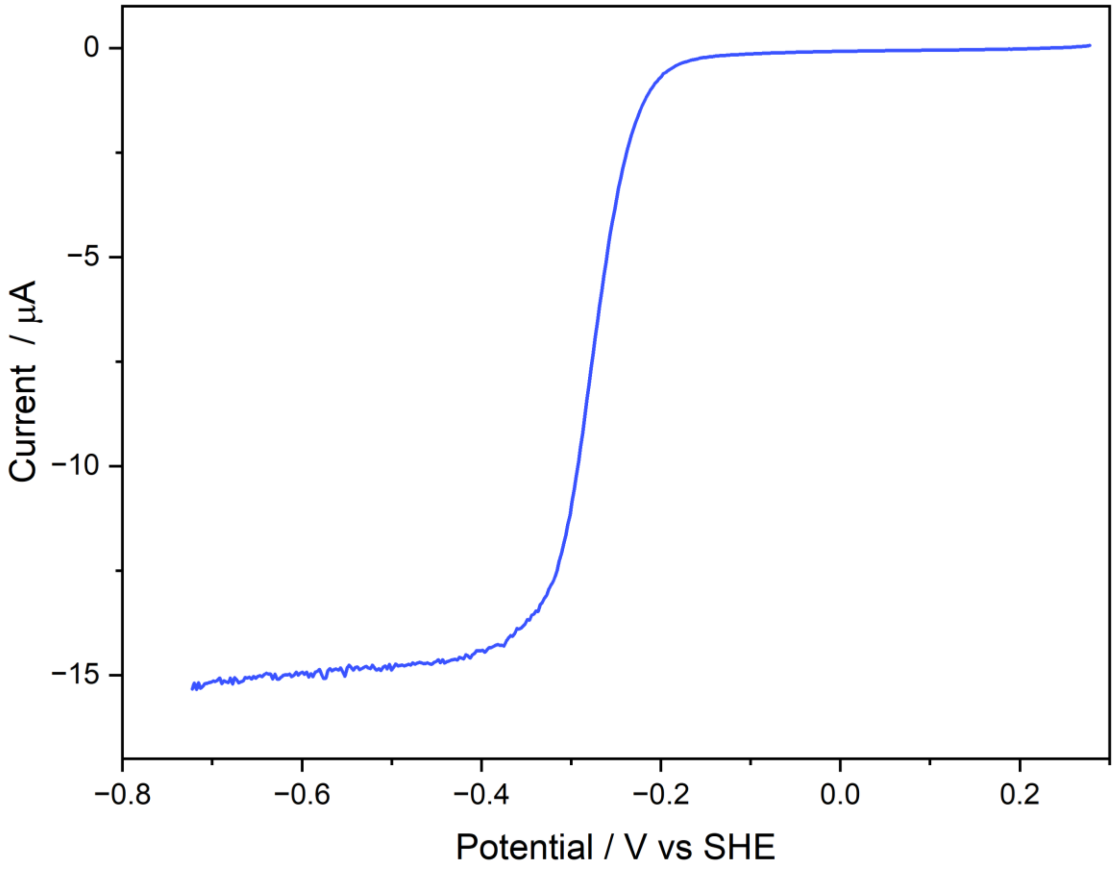

Supplementary Figure 53. Linear sweep voltammogram (0.01 V/s, 0.0024 V step) of substrate **5** in aqueous PB (50mM, pH 6.0) at 3000 rpm.

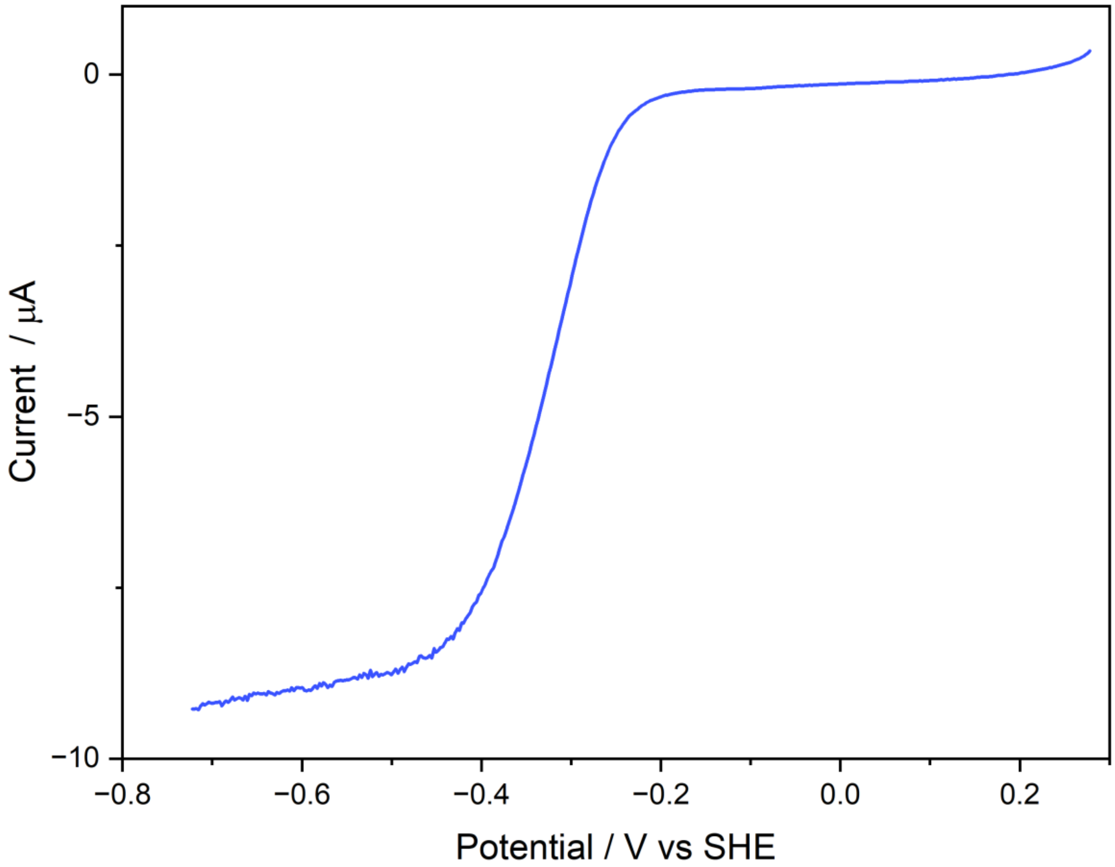

Supplementary Figure 54. Linear sweep voltammogram (0.01 V/s, 0.0024 V step) of substrate **6** in aqueous PB (50mM, pH 6.0) at 3000 rpm.

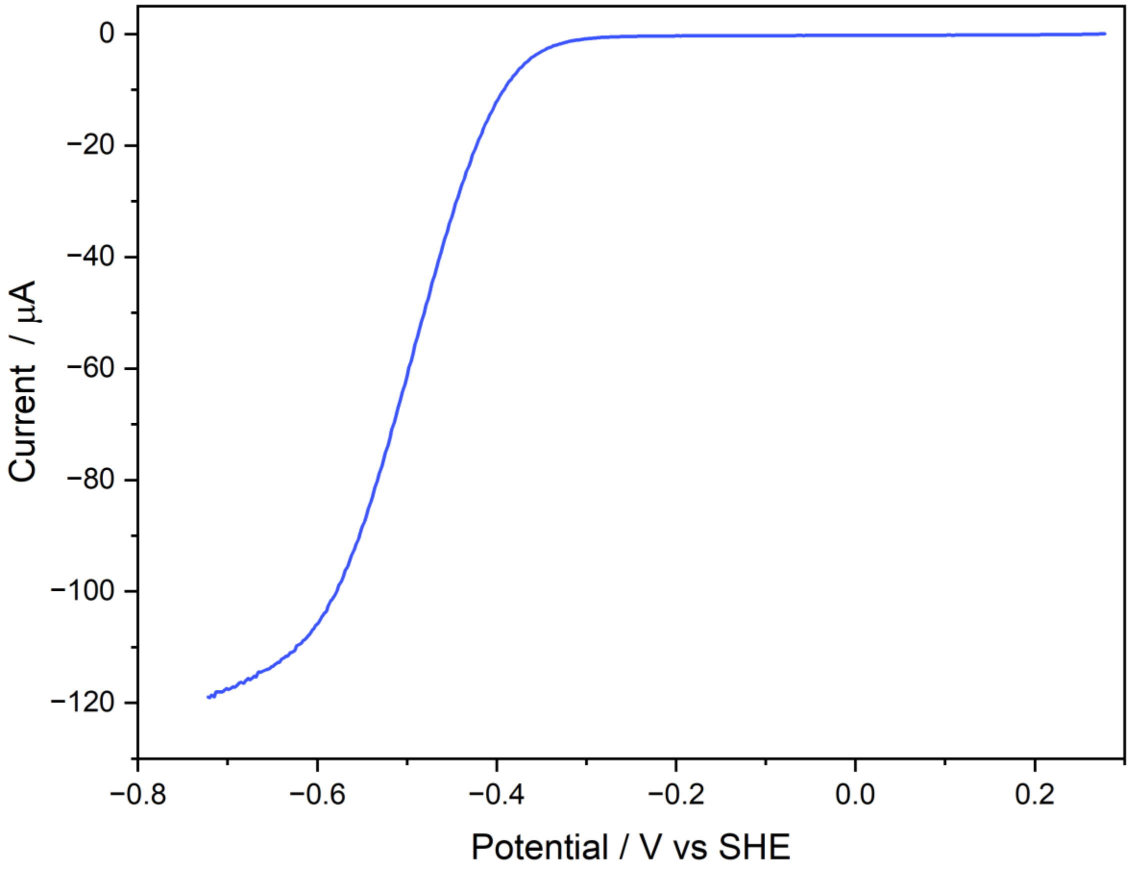

Supplementary Figure 55. Linear sweep voltammogram (0.01 V/s, 0.0024 V step) of substrate **7** in aqueous PB (50mM, pH 6.0) at 3000 rpm.

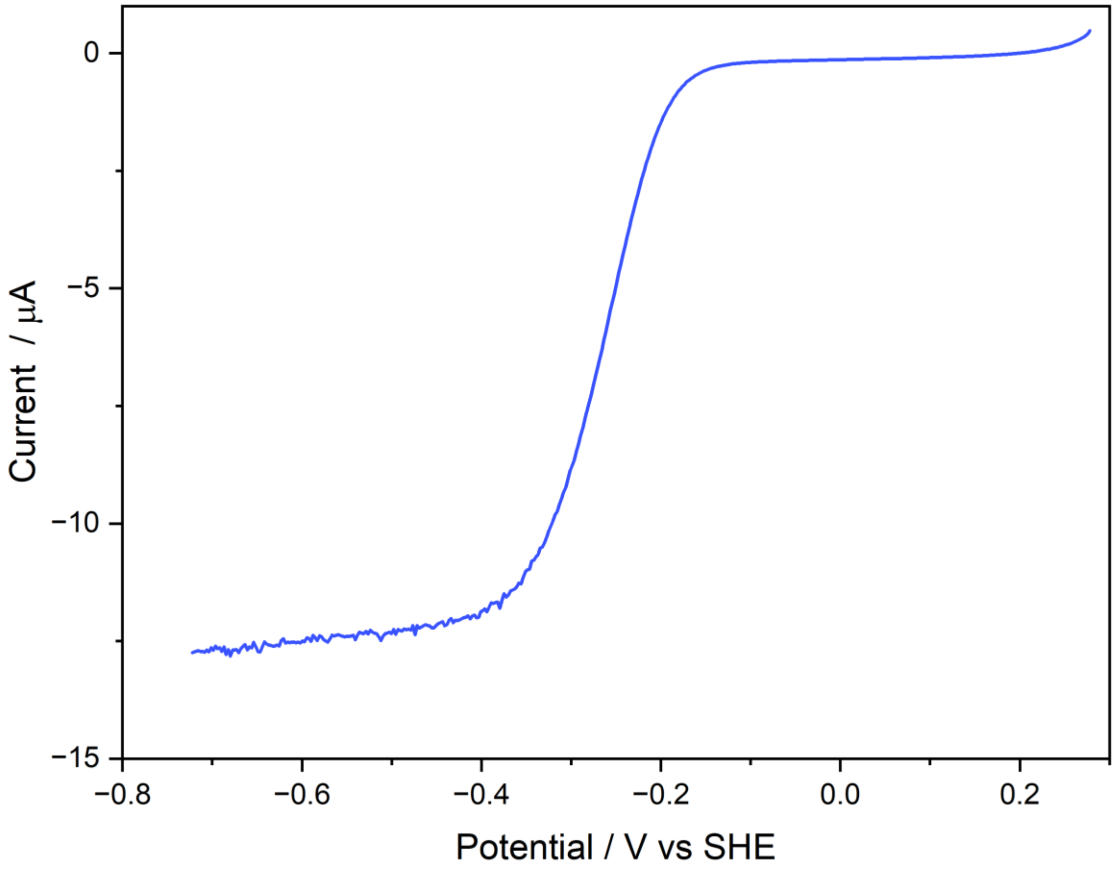

Supplementary Figure 56. Linear sweep voltammogram (0.01 V/s, 0.0024 V step) of substrate **10** in aqueous PB (50mM, pH 6.0) at 4000 rpm.

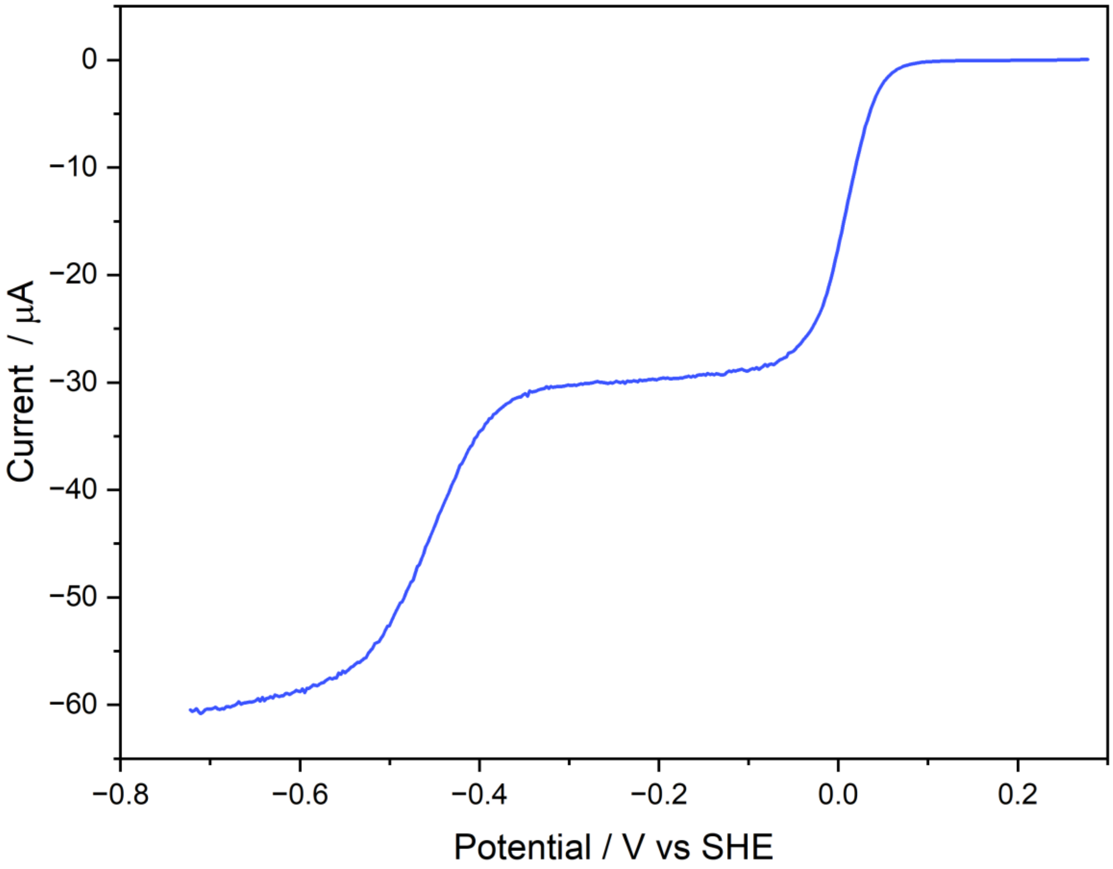

Supplementary Figure 57. Linear sweep voltammogram (0.01 V/s, 0.0024 V step) of substrate **13** in aqueous PB (50mM, pH 6.0) at 3000 rpm. Second reduction close to the thermodynamic onset.

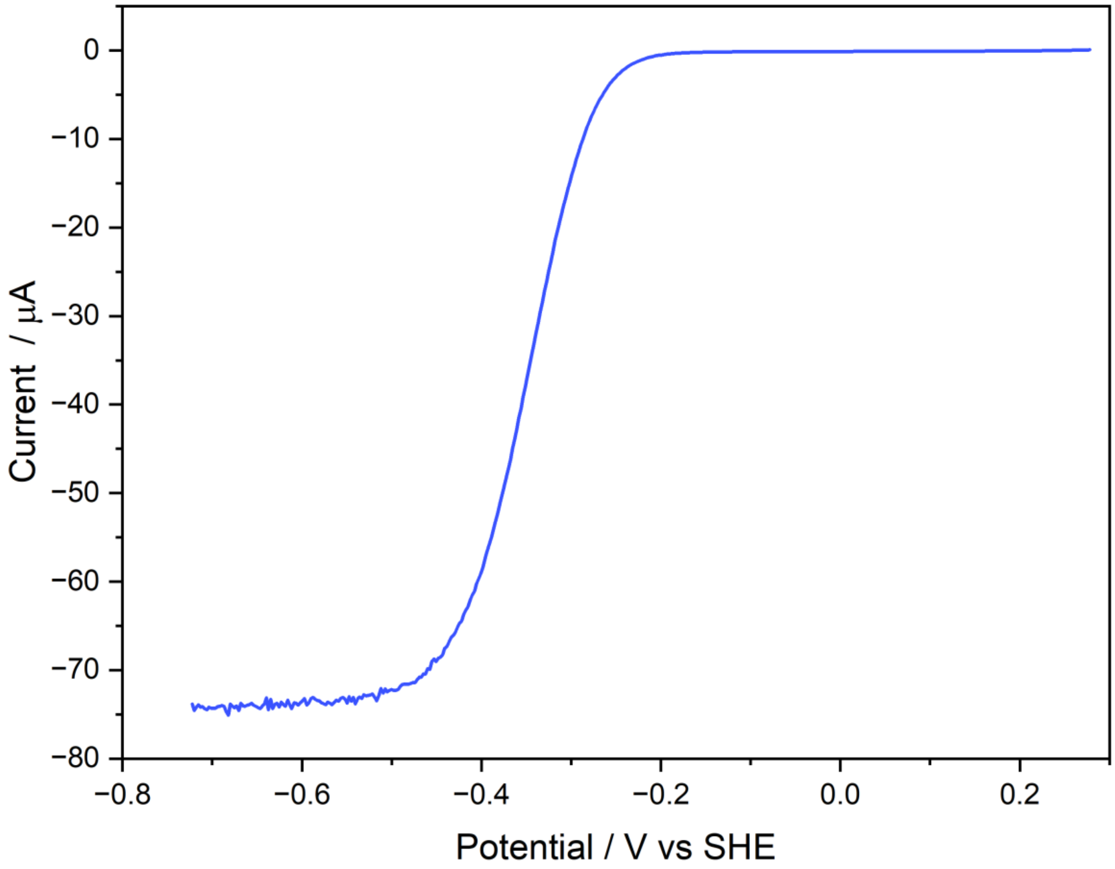

Supplementary Figure 58. Linear sweep voltammogram (0.01 V/s, 0.0024 V step) of substrate **14** in aqueous PB (50mM, pH 6.0) at 3000 rpm.

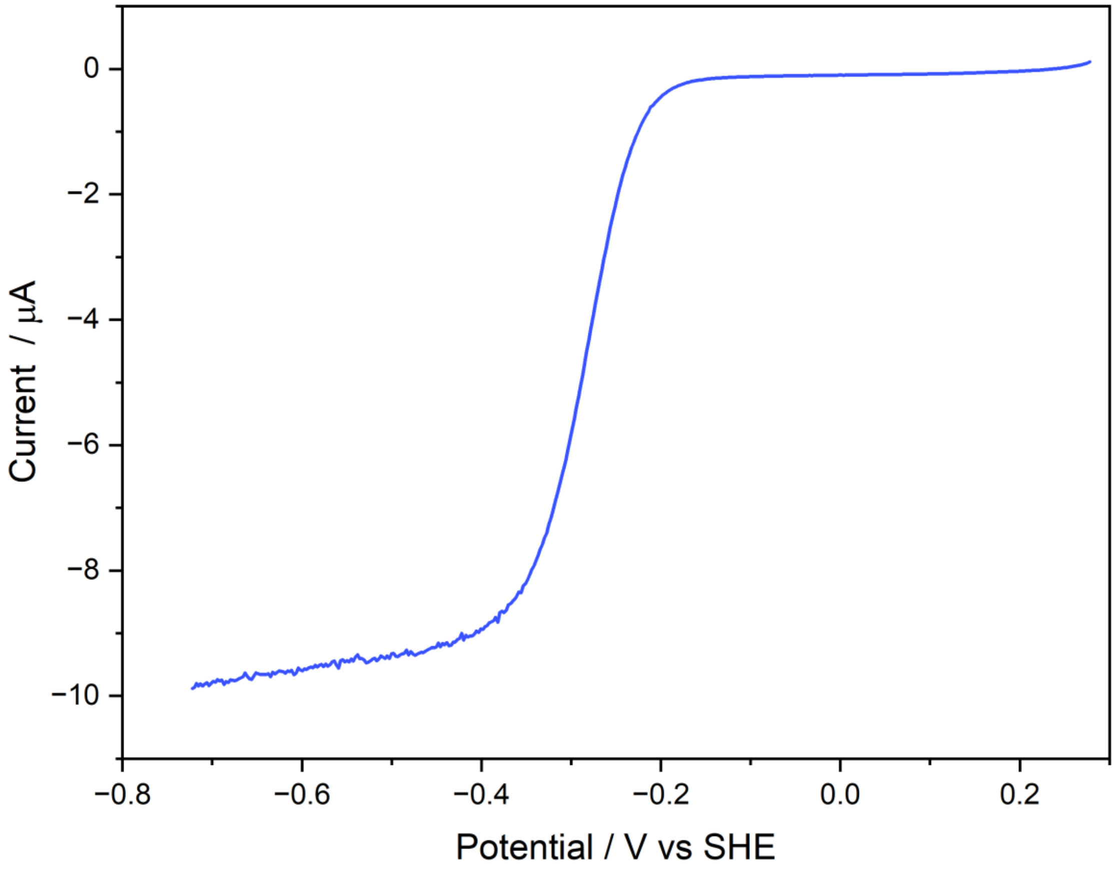

Supplementary Figure 59. Linear sweep voltammogram (0.01 V/s, 0.0024 V step) of substrate **15** in aqueous PB (50mM, pH 6.0) at 3000 rpm.

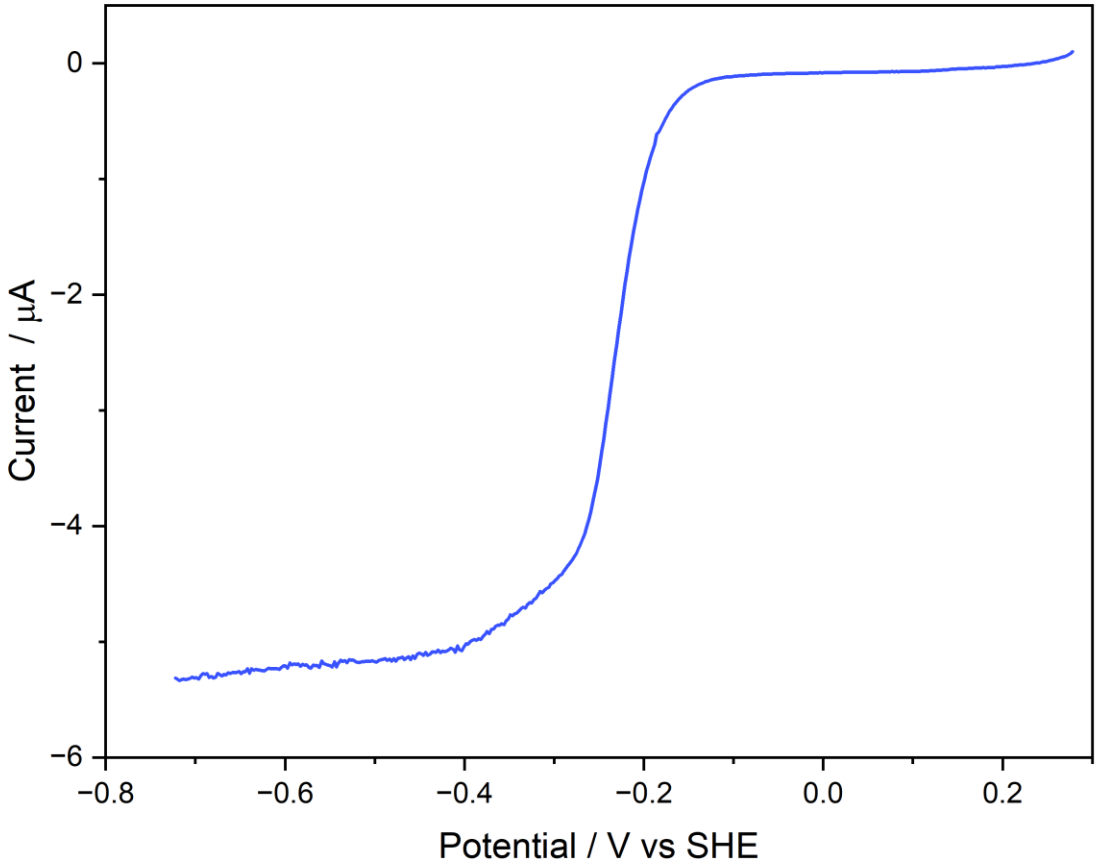

Supplementary Figure 60. Linear sweep voltammogram (0.01 V/s, 0.0024 V step) of substrate **16** in aqueous PB (50mM, pH 6.0) at 3000 rpm.

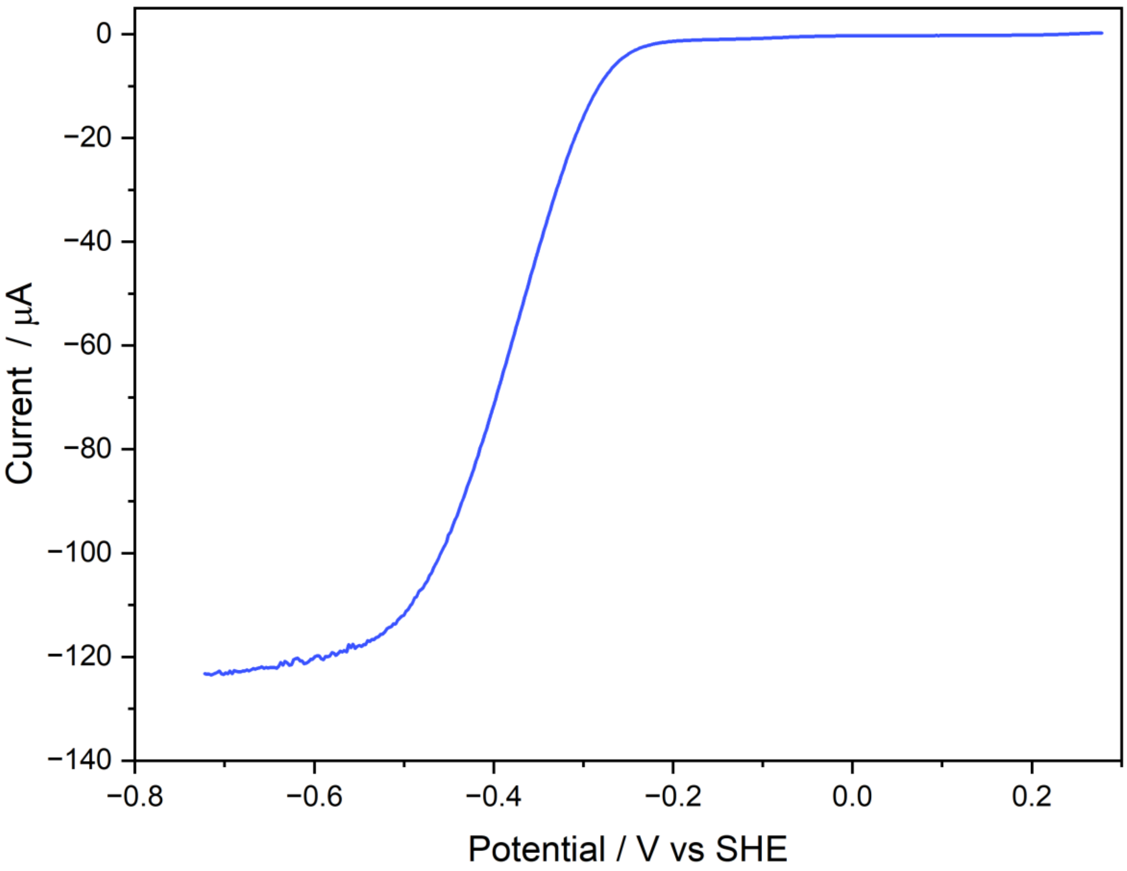

Supplementary Figure 61. Linear sweep voltammogram (0.01 V/s, 0.0024 V step) of substrate **17** in aqueous PB (50mM, pH 6.0) at 3000 rpm.

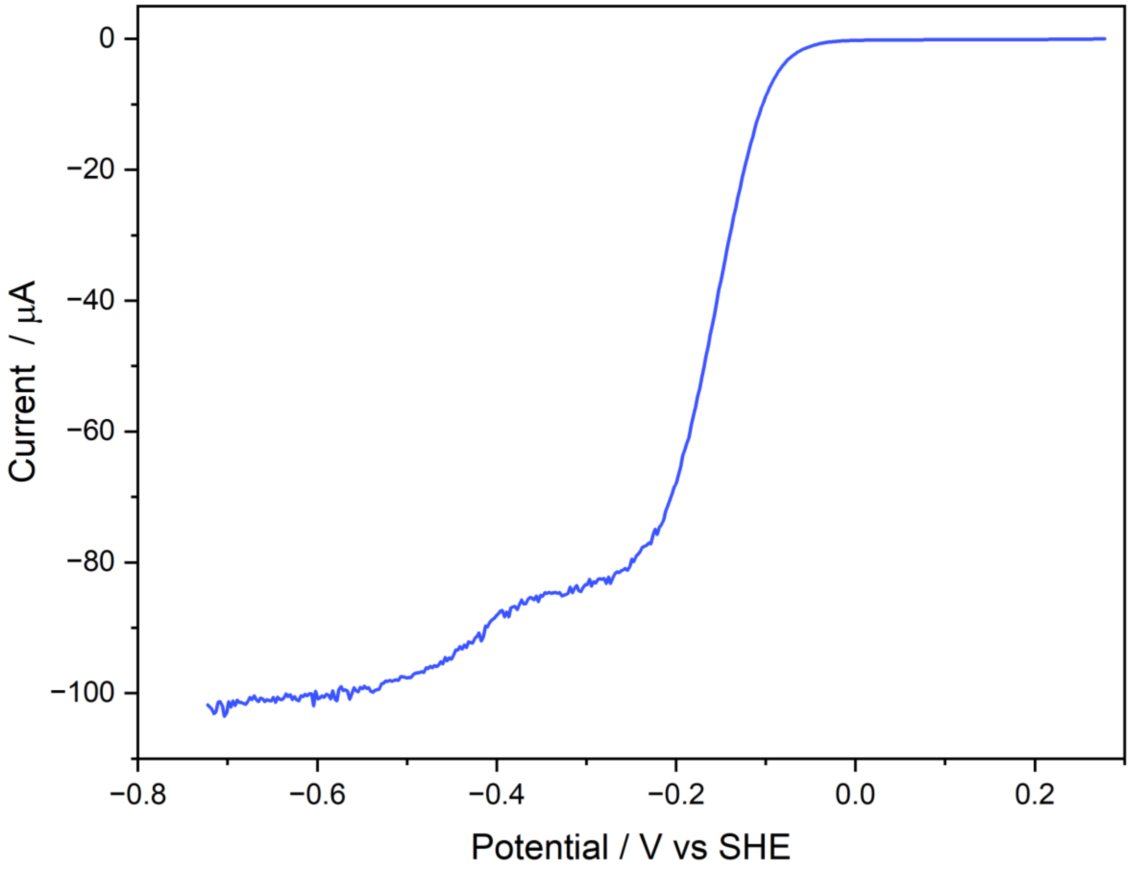

Supplementary Figure 62. Linear sweep voltammogram (0.01 V/s, 0.0024 V step) of substrate **18** in aqueous PB (50mM, pH 6.0) at 3000 rpm. The second smaller reduction visible here below -0.4 V may be caused by to the aldehyde functional group present on the substrate condensing with the amine product formed at the electrode and then consequently this condensation product may also be reduced forming a second wave.

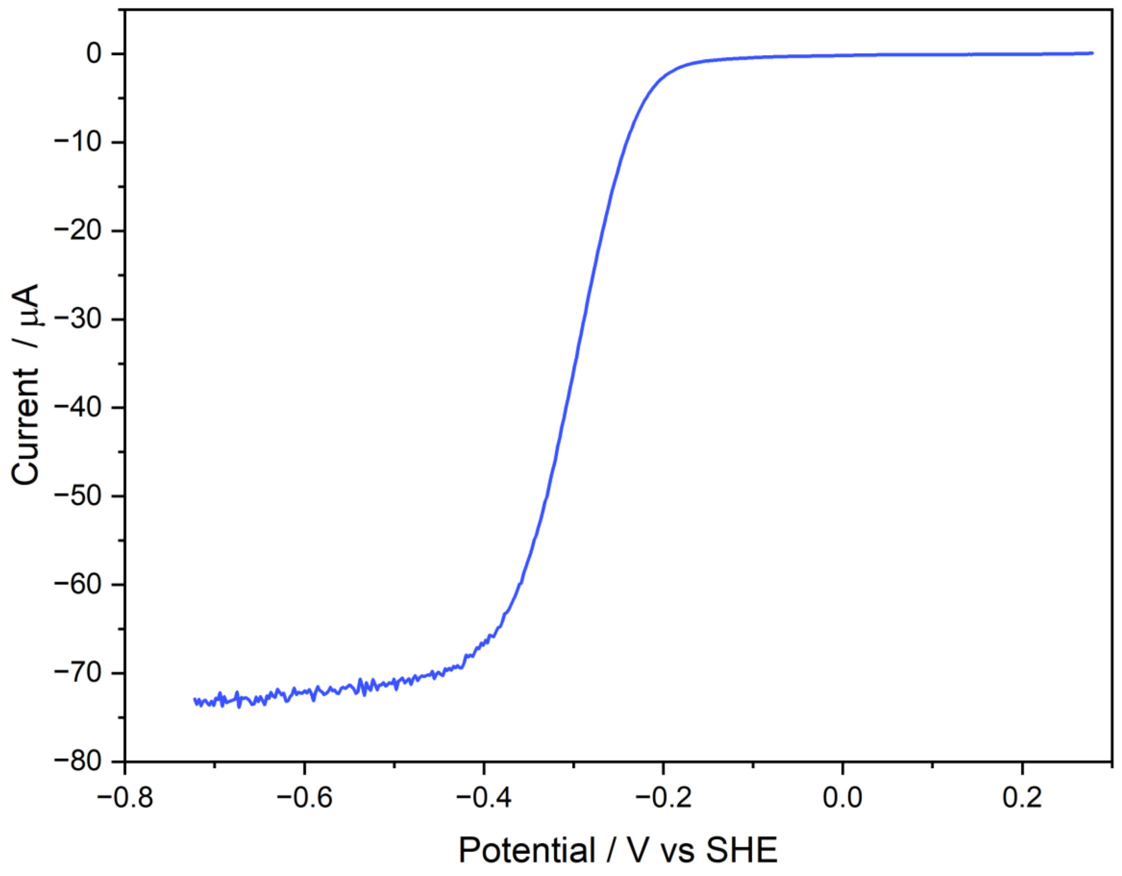

Supplementary Figure 63. Linear sweep voltammogram (0.01 V/s, 0.0024 V step) of substrate **19** in aqueous PB (50mM, pH 6.0) at 3000 rpm.

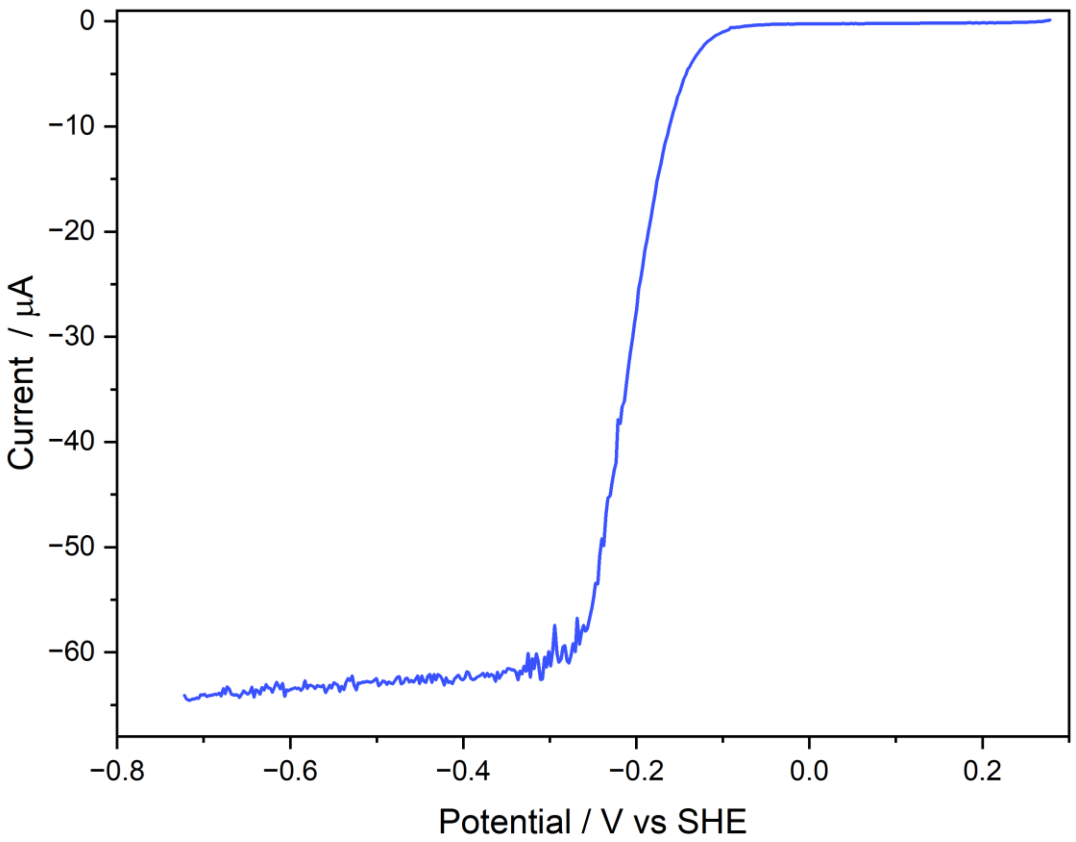

Supplementary Figure 64. Linear sweep voltammogram (0.01 V/s, 0.0024 V step) of substrate **20** in aqueous PB (50mM, pH 6.0) at 7000 rpm.

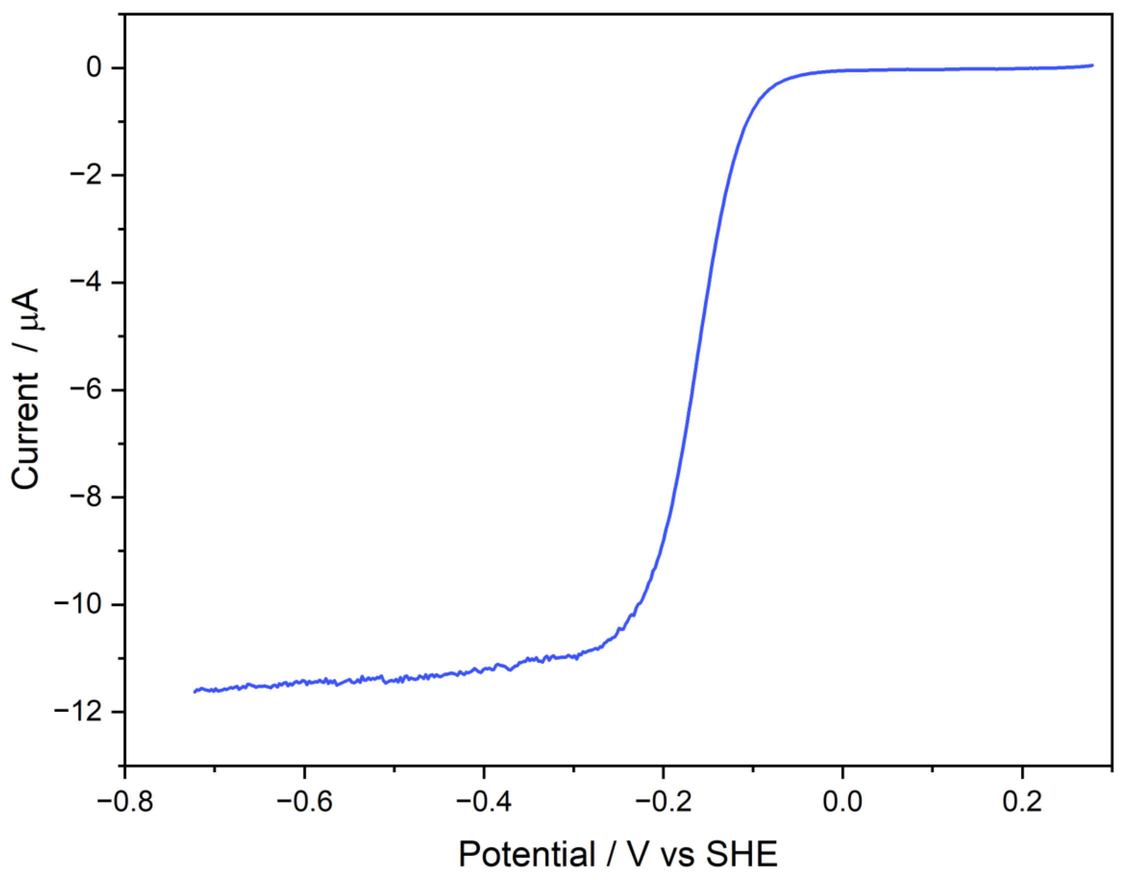

Supplementary Figure 65. Linear sweep voltammogram (0.01 V/s, 0.0024 V step) of substrate **21** in aqueous PB (50mM, pH 6.0) at 3000 rpm.

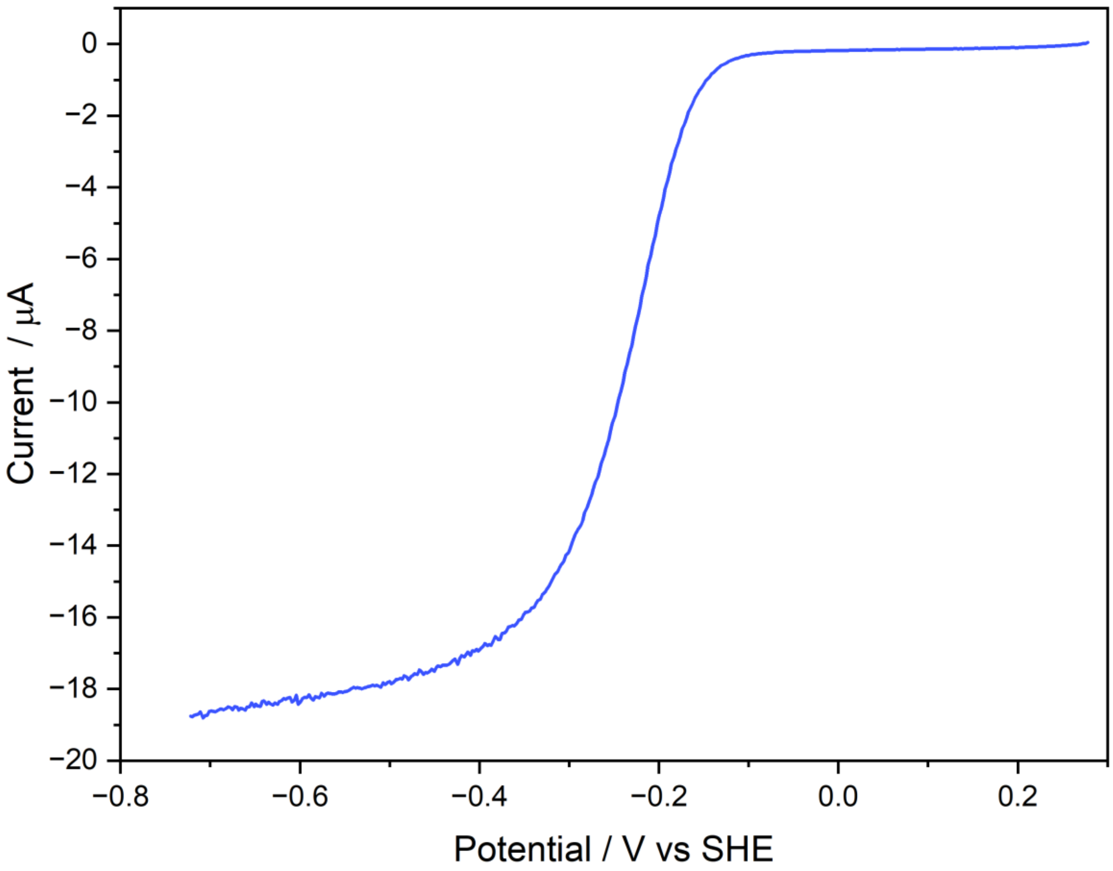

Supplementary Figure 66. Linear sweep voltammogram vs SHE (0.01 V/s, 0.0024 V step) of substrate **22** in aqueous PB (50mM, pH 6.0) at 3000 rpm.

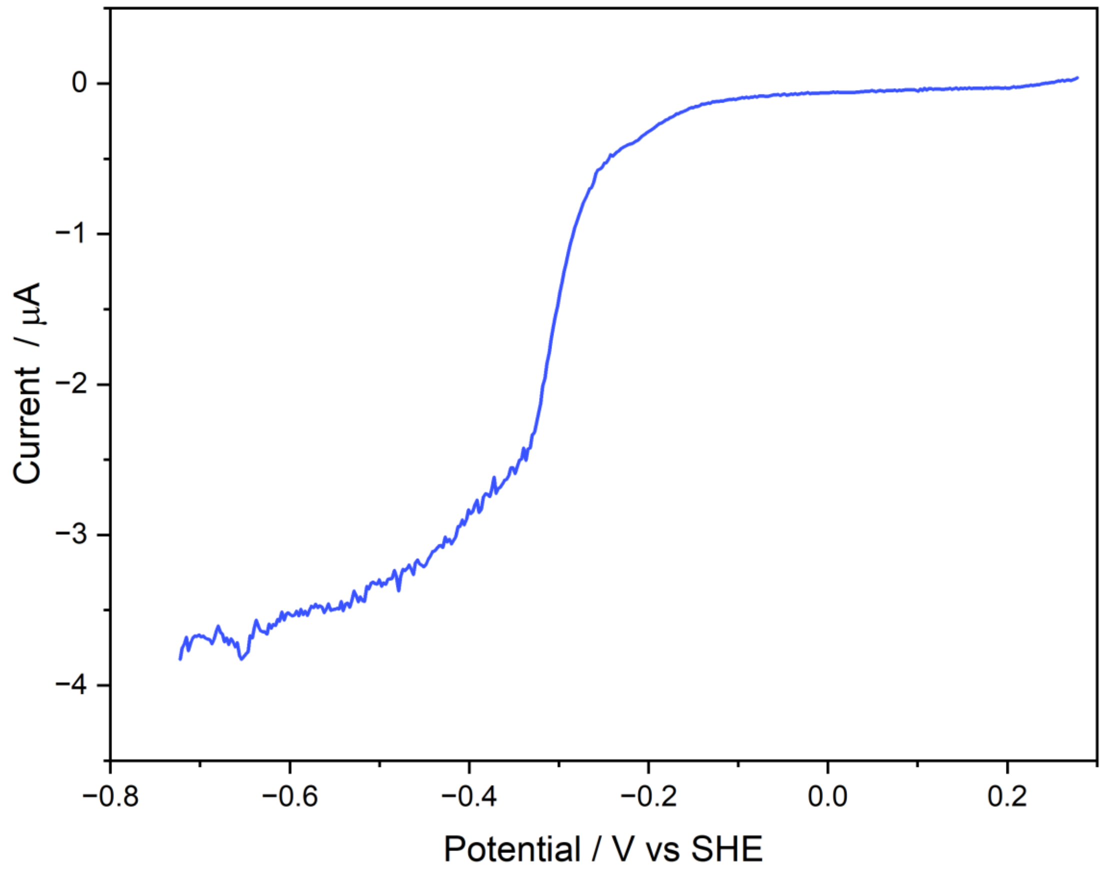

Supplementary Figure 67. Linear sweep voltammogram vs SHE (0.01 V/s, 0.0024 V step) of substrate **24** in aqueous PB (50mM, pH 6.0) at 5000 rpm. The increased speed here was required to overcome mass transport effects caused by the hydrophobicity of the *t*-Bu group which causes it to be present in high concentrations at the electrode surface at lower rotation rates.

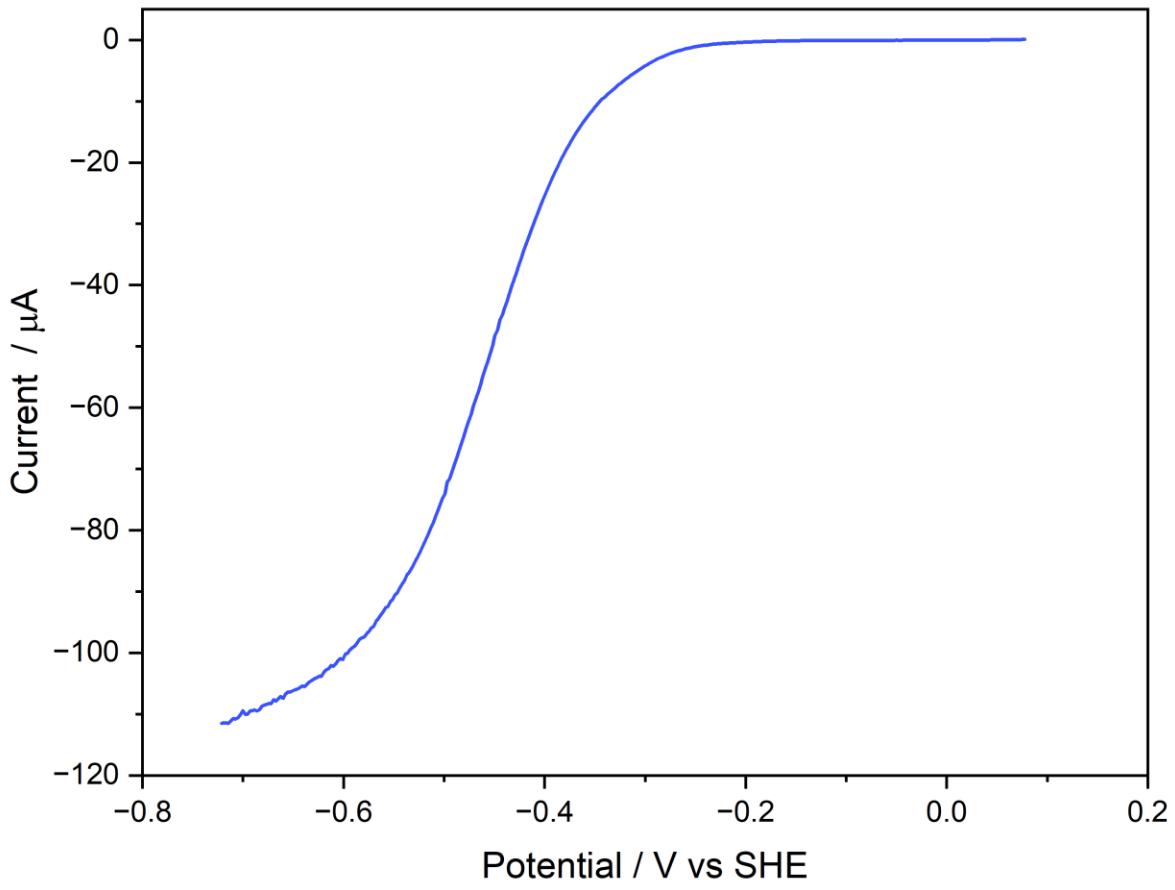

Supplementary Figure 68. Linear sweep voltammogram vs SHE (0.01 V/s, 0.0024 V step) of substrate **25** in aqueous PB (50mM, pH 6.0) at 4000 rpm.

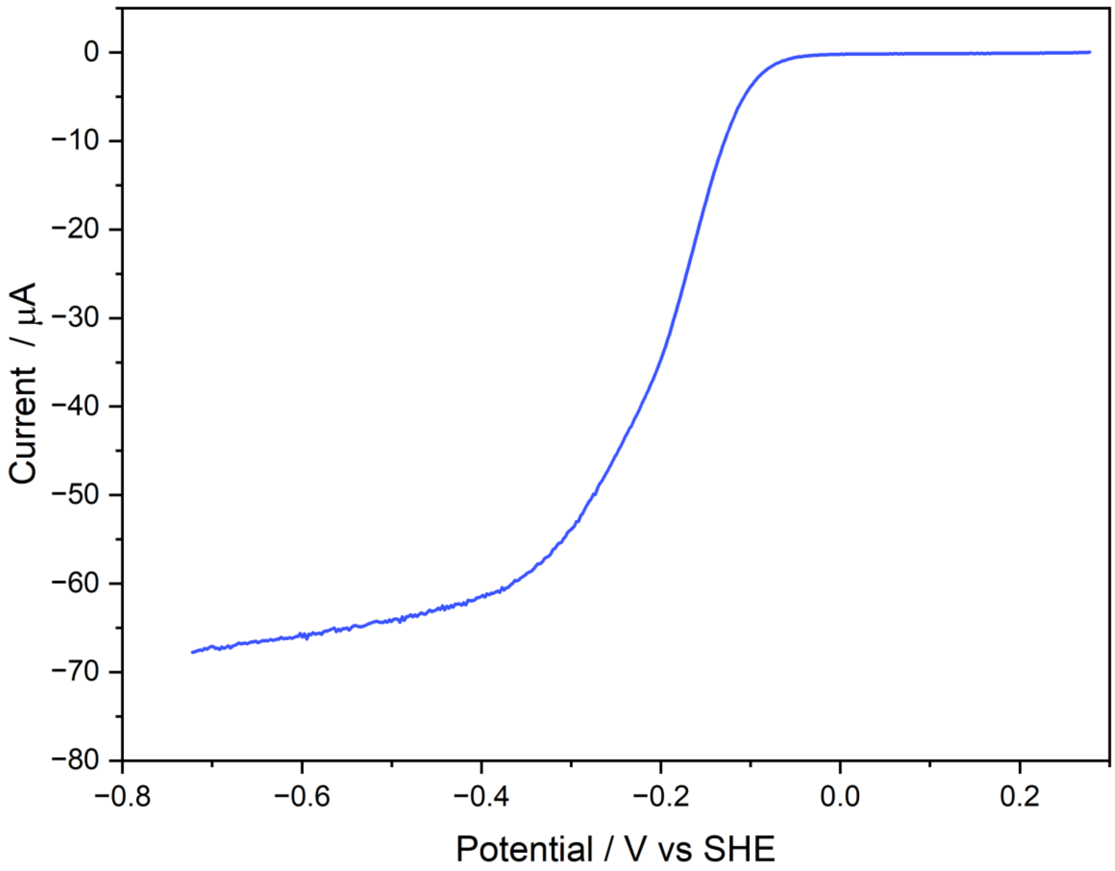

Supplementary Figure 69. Linear sweep voltammogram vs SHE (0.01 V/s, 0.0024 V step) of substrate **26** in aqueous PB (50mM, pH 6.0) at 3000 rpm.

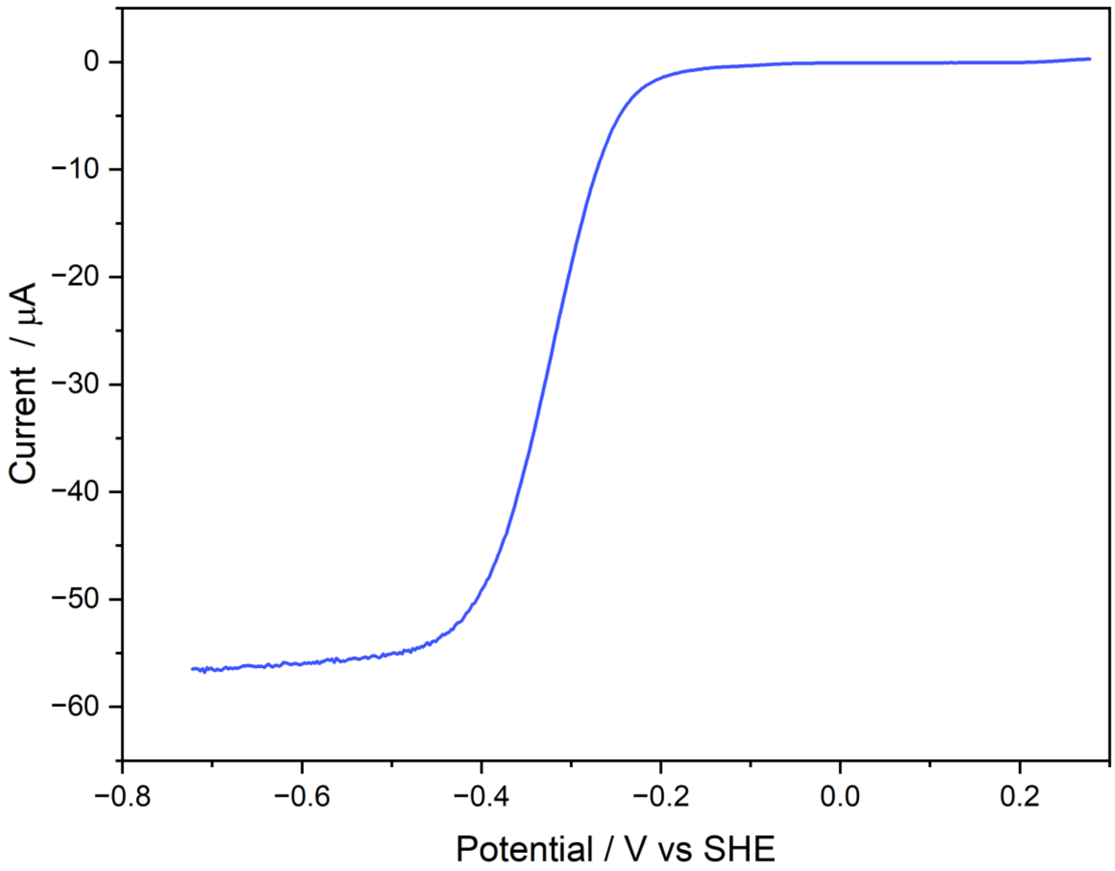

Supplementary Figure 70. Linear sweep voltammogram vs SHE (0.01 V/s, 0.0024 V step) of substrate **27** in aqueous PB (50mM, pH 6.0) at 3000 rpm.

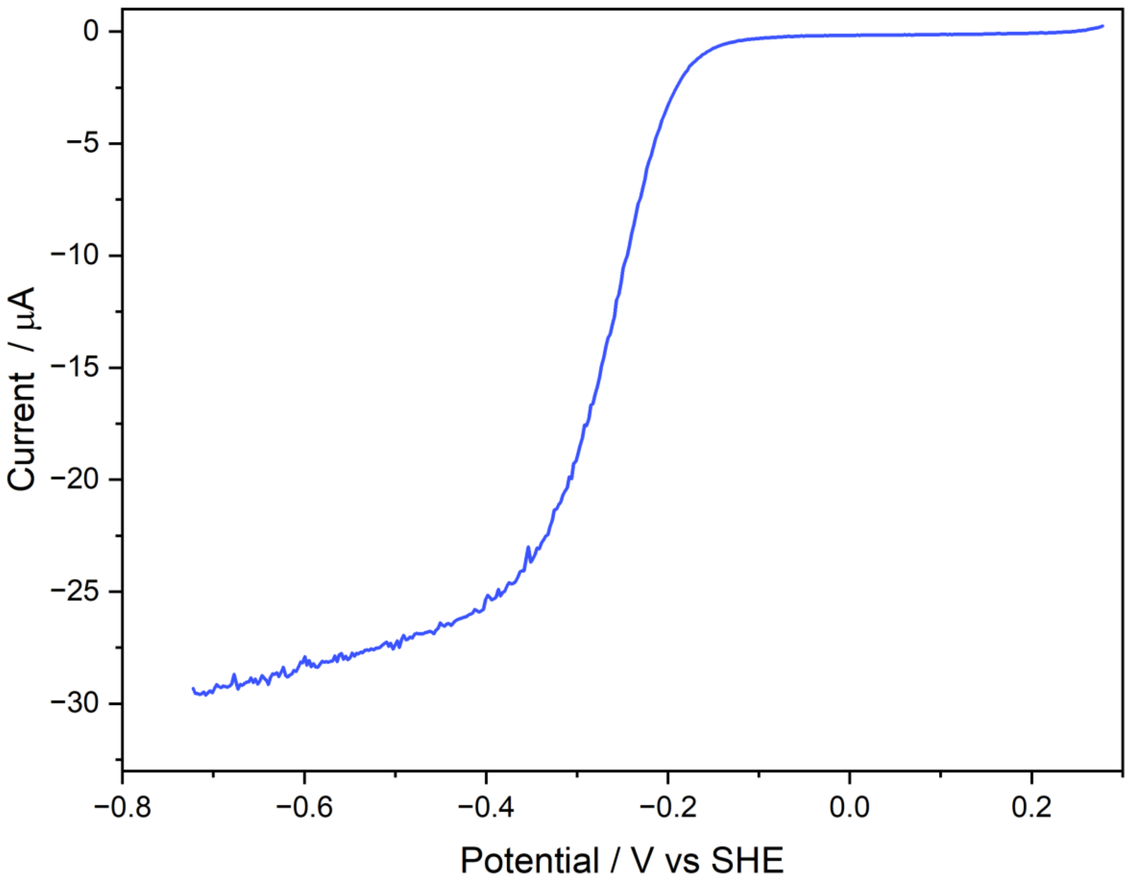

Supplementary Figure 71. Linear sweep voltammogram vs SHE (0.01 V/s, 0.0024 V step) of substrate **28** in aqueous PB (50mM, pH 6.0) at 6000 rpm.

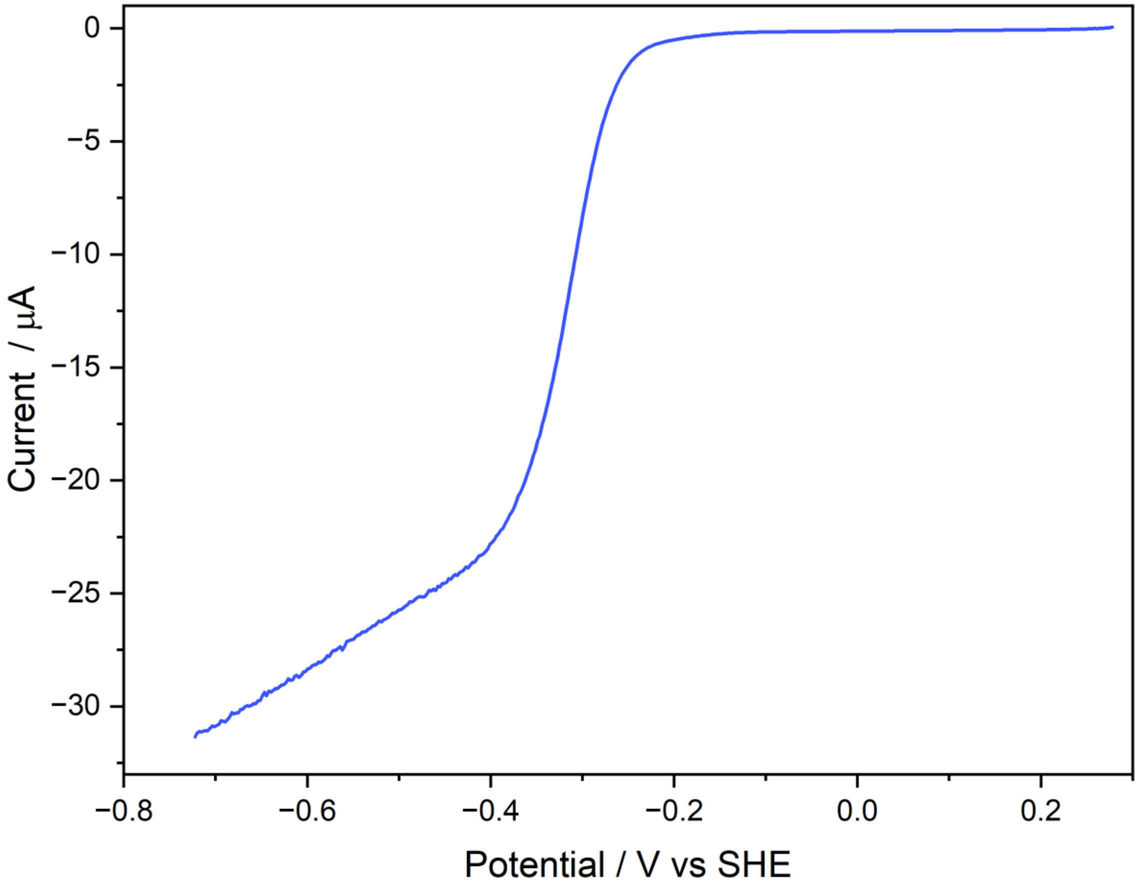

Supplementary Figure 72. Linear sweep voltammogram vs SHE (0.01 V/s, 0.0024 V step) of substrate **28** in aqueous PB (50mM, pH 8) at 6000 rpm.

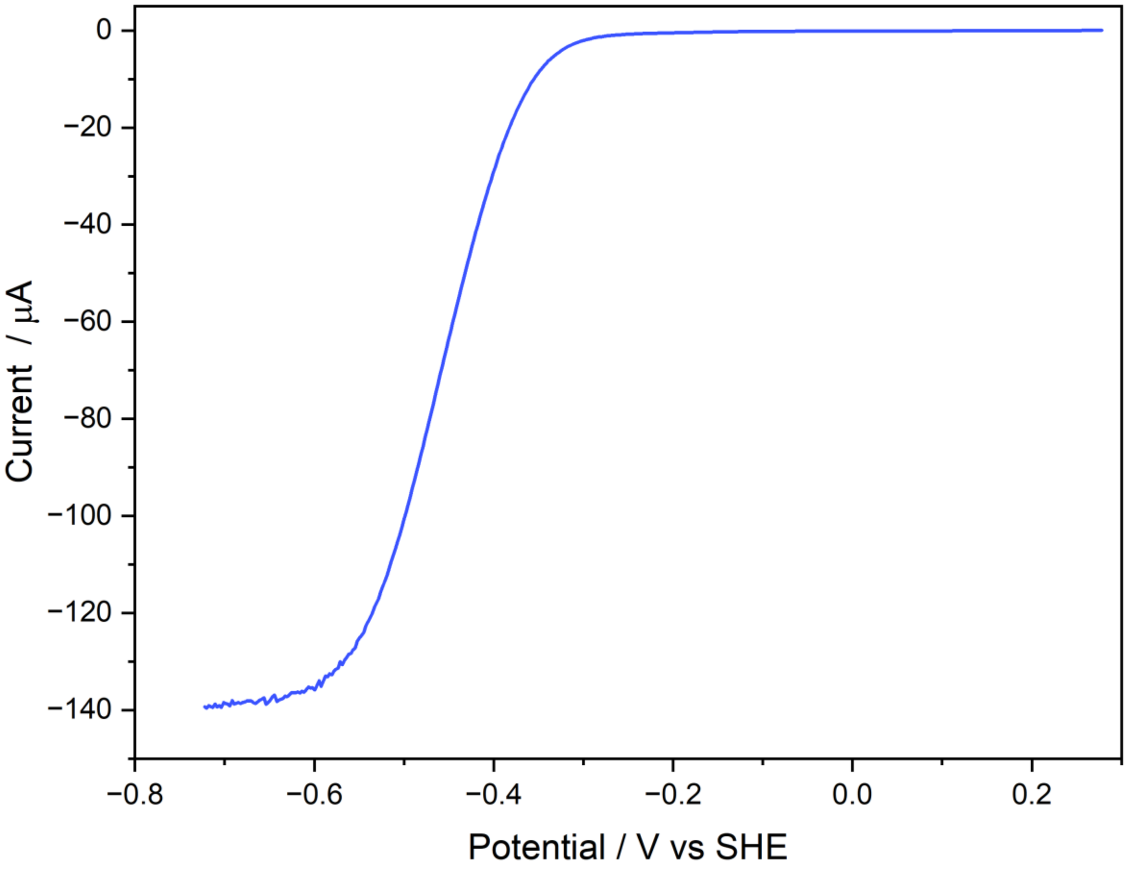

Supplementary Figure 73. Linear sweep voltammogram vs SHE (0.01 V/s, 0.0024 V step) of substrate **30** in aqueous PB (50mM, pH 6.0) at 3000 rpm.

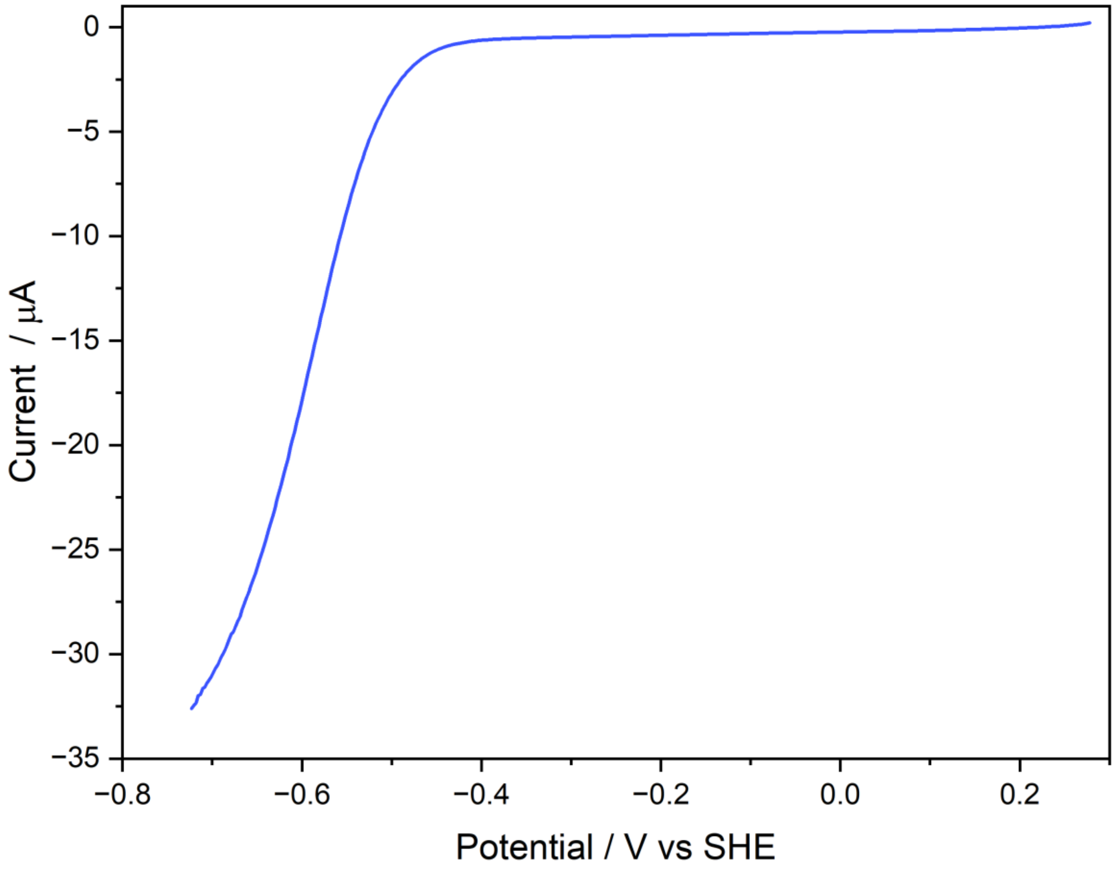

Supplementary Figure 74. Linear sweep voltammogram vs SHE (0.01 V/s, 0.0024 V step) of substrate **32** in aqueous PB (50mM, pH 6.0) at 3000 rpm.

X. Extension of Small-Scale Hydrogenation to Aliphatic Substrate

Catalyst preparation was carried out in a glove box (GLOVE BOX TECHNOLOGY LTD.) under a protective N_2 atmosphere (O_2 < 3 ppm). A 20 mg/mL carbon black (BP2000, CABOT) suspension in PB (50 mM, pH 6.0) was sonicated for 1 hour. For the preparation of the catalyst loading for one 1 mL scale reaction with 10 mM concentration of substrate, 7.5 µL of this suspension was transferred to an EPPENDORF tube, 2.13 µL of Hyd-2 solution (6.3 mg/mL) was added (C:Hyd-2 = 5.6:1 mass ratio), the mixture was gently mixed and left on ice for 1 hour. After that, the suspension of the catalyst was centrifuged (3 min, 14100 × g), the supernatant was removed, and the catalyst was resuspended in 50 µL of PB (50 mM, pH 6.0). The catalyst was then directly added to the reaction vial. Reactions were run on a 0.5 mL scale with 10 mM of substrate in PB (50 mM, pH 6.0) in a pressure vessel reactor, charged to 2 bar H_2 and placed on a rocker to facilitate mixing. For ^{1}H-NMR analysis, the reaction mixture was centrifuged to collect the catalyst particles, 450 µL of supernatant and 50 µL of D_2O were added to the NMR tube. ^{1}H-NMR spectra were recorded with water signal suppression method.

Supplementary Table 7. Comparison of C:Hyd-2 ratios for the catalyst preparation for hydrogenation of **1**. The optimal catalyst with C:Hyd-2 = 5.6:1 mass ratio is highlighted in blue, this corresponds to **0.3 mg Hyd-2 immobilised on carbon per 1 mmol substrate**. Conversions and ratios of products were determined by integration of ^{1}H-NMR spectra. All reactions were run for 18 hours at 1 bar H_2 in a pressure vessel with stirring at 10 mM concentration of **1** in PB (50 mM, pH 6.0) using 0.08 mg of carbon and varied quantities of Hyd-2.

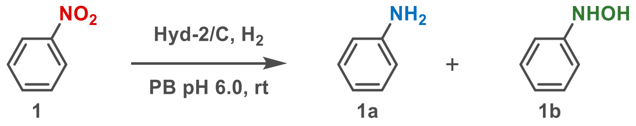

Entry	C:Hyd-2 ratio	1b:1a
1	2:1	34:66
2	3:1	19:81
3	4:1	2:98
4	5:1	4:96
5	5.6:1	0:100
6	6:1	28:72

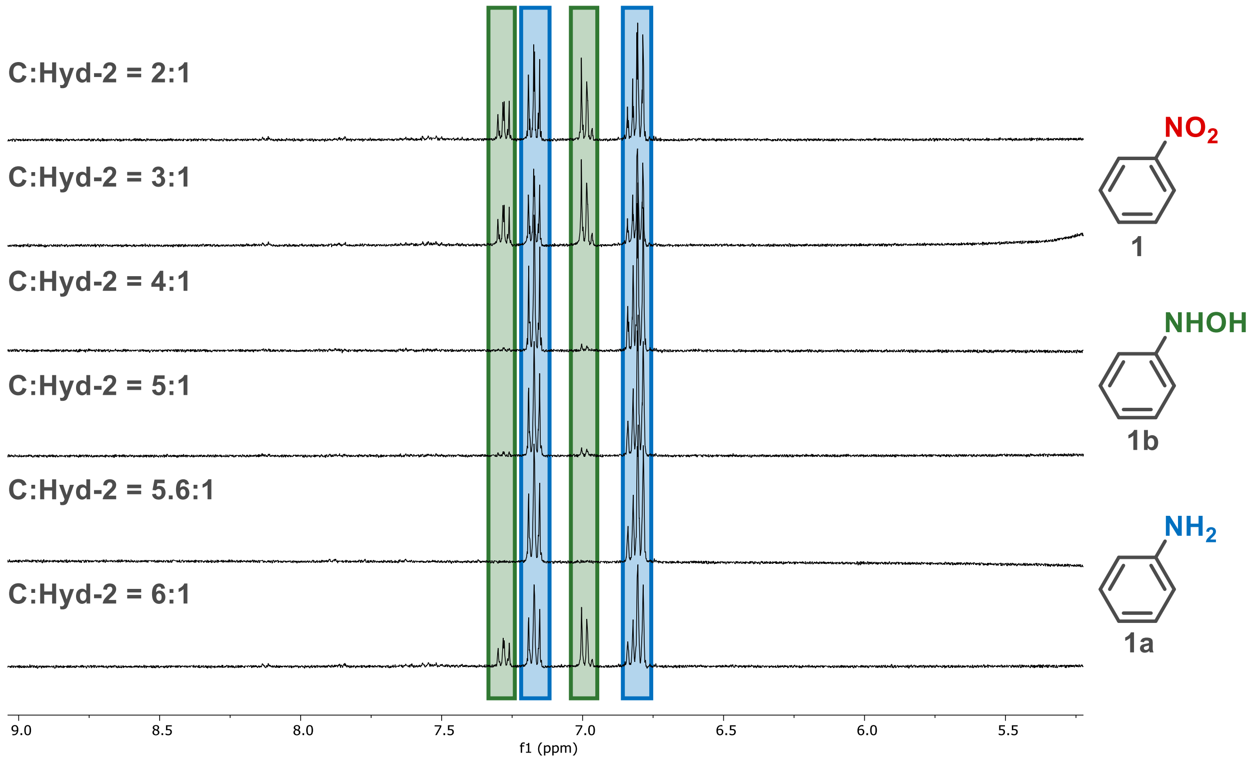

Supplementary Figure 75. ^{1}H-NMR spectra (400 MHz, 298 K, 10% D_2O in PB, 50 mM, pH 6.0) of reactions of Hyd-2/C with 10 mM nitrobenzene (**1**) in PB (50 mM, pH 6.0) after 18 hours at using 0.08 mg of carbon per 0.5 mL of reaction mixture, with Hyd-2 immobilised with variation of C:Hyd-2 ratio for the catalyst preparation (indicated on the left side of each spectrum). Catalyst ratio was optimised at 5.6:1 after which no *N*-phenylhydroxylamine (**1b**) is detected by ^{1}H-NMR. *N*-Phenylhydroxylamine (**1b**) is labelled with green markers and aniline (**1a**) is labelled with blue markers.

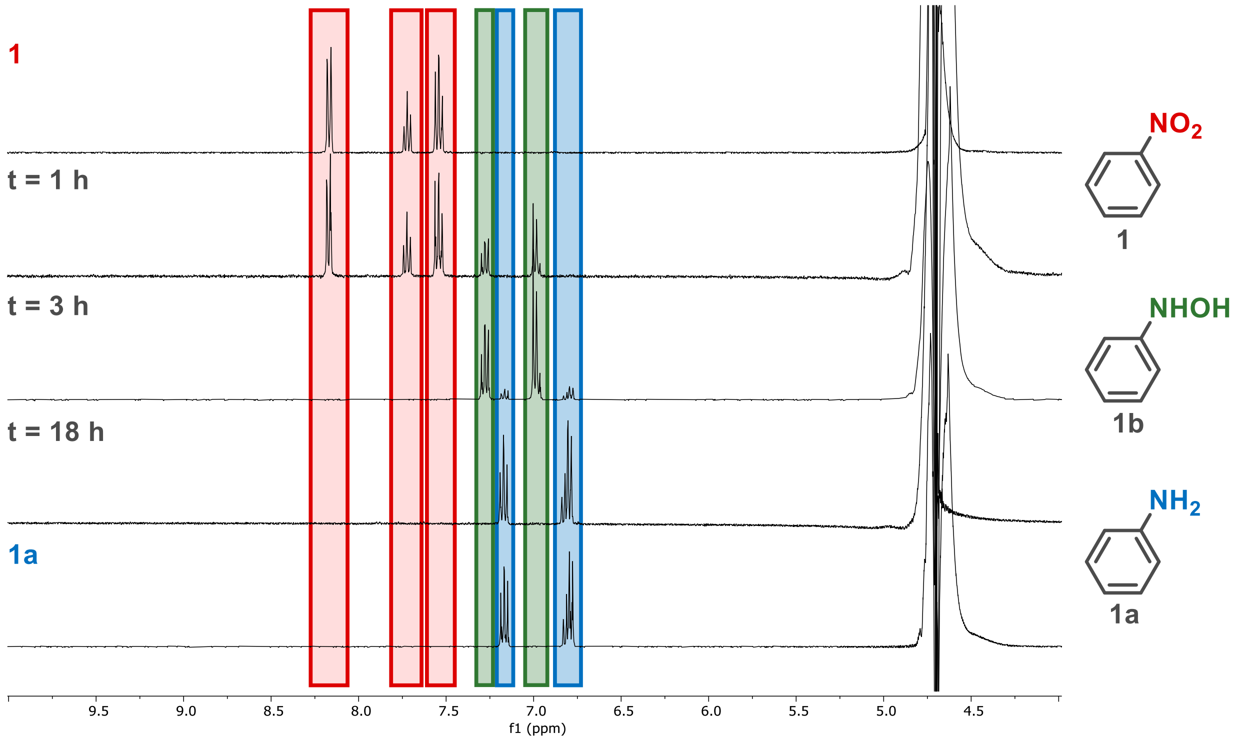

Supplementary Figure 76. ^{1}H-NMR spectra (400 MHz, 298 K, 10% D_2O in PB, 50 mM, pH 6.0) showing reaction progression of Hyd-2/C catalyst and nitrobenzene (**1**, red markers). Reaction pathway is observed to go *via* *N*-phenylhydroxylamine (**1b**, green markers) to aniline (**1a**, blue markers), synonymous with the Hyd-1/C pathway shown in Figure 4B of the Manuscript.

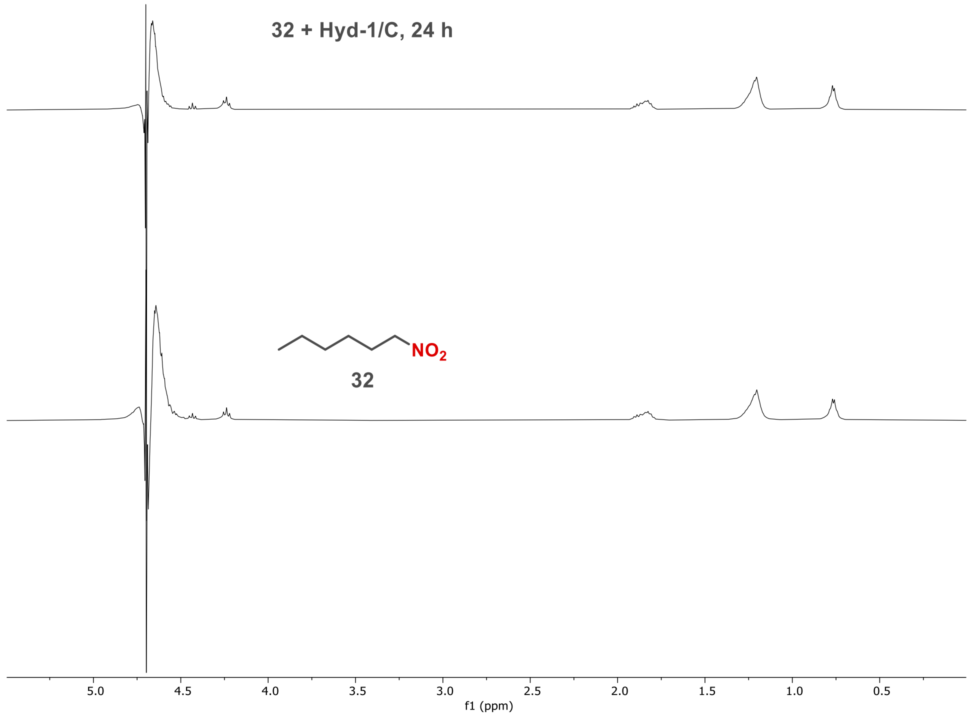

Supplementary Figure 77. ^{1}H-NMR spectra (400 MHz, 298 K, 10% D_2O in PB, 50 mM, pH 6.0) of substrate **32** and reaction mixture of its hydrogenation using Hyd-1/C catalyst after 24 hours. No conversion of **32** observed.

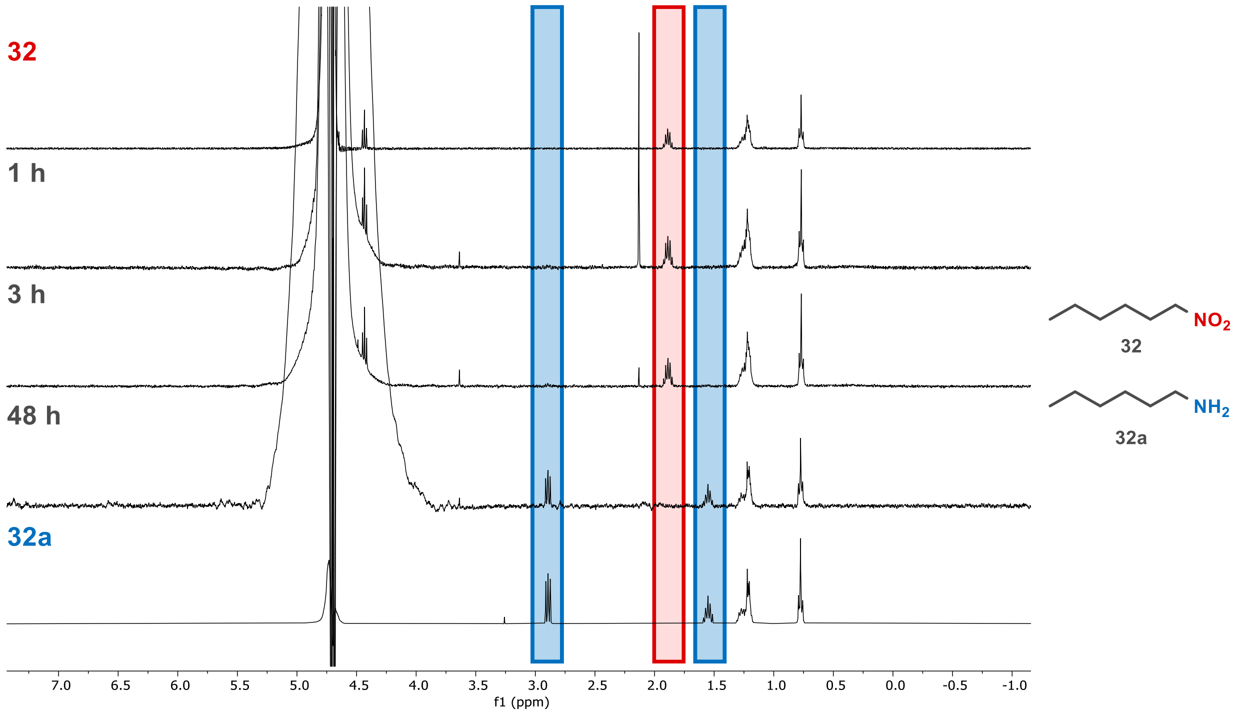

Supplementary Figure 78. ^{1}H-NMR spectra (400 MHz, 298 K, 10% D_2O in PB, 50 mM, pH 6.0) showing reaction progression of conversion of **32** to **32a** using Hyd-2/C catalyst. Characteristic peaks of 1-nitrohexane (**32**) are labelled with red markers) and of 1-aminohexane (**32a**) is labelled with blue markers.

B. Supplementary References

1. Ramirez, M. A. *et al.* H_2-Driven Reduction of Flavin by Hydrogenase Enables Cleaner Operation of Nitroreductases for Nitro-Group to Amine Reductions. *Front. Catal.* **2**, 906694 (2022).

2. Beaton, S. E. *et al.* The structure of hydrogenase-2 from Escherichia coli: implications for H_2-driven proton pumping. *Biochem. J.* **475**, 1353–1370 (2018).

3. Shirai, T. *et al.* Decarbonylation through Aldehydic C–H Bond Cleavage by a Cationic Iridium Catalyst. *Synlett* **30**, 972–976 (2019).

4. Li, H. *et al.* Controllable redox reaction cycle enabled by multifunctional Ru-containing polyoxometalate-based catalysts. *J. Mater. Chem. A* **11**, 10813–10822 (2023).

5. Robles-Henríquez, R. *et al.* Selective reduction of nitroarenes using Ru/C and CaH_2. *Org. Biomol. Chem.* **21**, 187–194 (2022).

6. Goyal, V. *et al.* Methanol as a Potential Hydrogen Source for Reduction Reactions Enabled by a Commercial Pt/C Catalyst. *J. Org. Chem.* **88**, 2245–2259 (2023).

7. Song, D. *et al.* A recyclable ciprofloxacin polymer ligand for copper-catalyzed coupling of (hetero)aryl halide aminations. *New J. Chem.* **46**, 19100–19103 (2022).

8. Panda, S., Nanda, A., Behera, R. R., Ghosh, R. & Bagh, B. Cobalt catalyzed chemoselective reduction of nitroarenes: hydrosilylation under thermal and photochemical reaction conditions. *Chem. Commun* **59**, 4527 (2023).

9. Gawande, M. B. *et al.* Regio- and Chemoselective Reduction of Nitroarenes and Carbonyl Compounds over Recyclable Magnetic Ferrite−Nickel Nanoparticles (Fe_3O_4−Ni) by Using Glycerol as a Hydrogen Source. *Chem. – A Eur. J.* **18**, 12628–12632 (2012).

10. Liu, Y. *et al.* Rhodium-terpyridine Catalyzed Transfer Hydrogenation of Aromatic Nitro Compounds in Water. *Chem. – An Asian J.* **16**, 1725–1729 (2021).

11. Behera, R. R., Panda, S., Ghosh, R., Kumar, A. A. & Bagh, B. Manganese-Catalyzed Chemoselective Hydrosilylation of Nitroarenes: Sustainable Route to Aromatic Amines. *Org. Lett.* **24**, 9179–9183 (2022).

12. Papadas, I. T., Fountoulaki, S., Lykakis, I. N. & Armatas, G. S. Controllable Synthesis of Mesoporous Iron Oxide Nanoparticle Assemblies for Chemoselective Catalytic Reduction of Nitroarenes. *Chem. – A Eur. J.* **22**, 4600–4607 (2016).

13. Sharma, S., Anjani, N., Kouser, M. & Gupta, M. Palladium nanoparticles loaded over sheet-like N-doped graphene oxide: investigation of its catalytic potential in Suzuki coupling, in reduction of nitroarenes and in photodegradation of methyl orange. *New J. Chem.* **47**, 16030–16042 (2023).

14. Widegren, M. B., Harkness, G. J., Slawin, A. M. Z., Cordes, D. B. & Clarke, M. L. A Highly

Active Manganese Catalyst for Enantioselective Ketone and Ester Hydrogenation. *Angew. Chemie - Int. Ed.* **56**, 5825–5828 (2017).

15. Tao, C. *et al.* Room-Temperature Copper-Catalyzed Synthesis of Primary Arylamines from Aryl Halides and Aqueous Ammonia. *Synlett* **9**, 1355–1358 (2010).

16. Goldschmid, S. L. *et al.* Overcoming Photochemical Limitations in Metallaphotoredox Catalysis: Red-Light-Driven C-N Cross-Coupling. *J. Am. Chem. Soc.* **144**, 22409–22415 (2022).

17. Zhao, H. & Leonori, D. Minimization of Back-Electron Transfer Enables the Elusive sp3 C−H Functionalization of Secondary Anilines. *Angew. Chemie Int. Ed.* **60**, 7669–7674 (2021).

18. Qi, H. L., Chen, D. S., Ye, J. S. & Huang, J. M. Electrochemical technique and copper-promoted transformations: Selective hydroxylation and amination of arylboronic acids. *J. Org. Chem.* **78**, 7482–7487 (2013).

19. Zhang, J. *et al.* Design, synthesis, and structure-activity relationship of 7-propanamide benzoxaboroles as potent anticancer agents. *J. Med. Chem.* **62**, 6765–6784 (2019).

20. Price, J. D., Piccariello, T., Oberlender, R. A. & Palmer, S. B. Bismuth-containing compounds, coordination polymers, methods for modulating pharmacokinetic properties of biologically active agents, and methods for treating patients (2018).

21. Jiang, C. S. *et al.* Discovery of 4-benzoylamino-N-(prop-2-yn-1-yl)benzamides as novel microRNA-21 inhibitors. *Bioorg. Med. Chem.* **23**, 6510–6519 (2015).

22. Boyle, M. *et al.* Amide Bond Formation via the Rearrangement of Nitrile Imines Derived from N-2-Nitrophenyl Hydrazonyl Bromides. *Org. Lett.* **24**, 334–338 (2022).

23. Tamura, M., Murase, D. & Komura, K. Direct Amide Synthesis from Equimolar Amounts of Carboxylic Acid and Amine Catalyzed by Mesoporous Silica SBA-15. *Synthesis* **47**, 769–776 (2015).

24. Lukey, M. J. *et al.* How Escherichia coli is equipped to oxidize hydrogen under different redox conditions. *J. Biol. Chem.* **285**, 20421 (2010).

25. Fortunato, G. V. *et al.* Analysing the relationship between the fields of thermo- and electrocatalysis taking hydrogen peroxide as a case study. *Nat. Commun.* **13**, 1–7 (2022).
